# Synthesis of Sorafenib−Ruthenium Complexes, Investigation of Biological Activities and Applications in Drug Delivery Systems as an Anticancer Agent

**DOI:** 10.1021/acs.jmedchem.3c01115

**Published:** 2024-03-12

**Authors:** Belma Zengin Kurt, Dilek Öztürk Civelek, Elmas Begüm Çakmak, Yakup Kolcuoğlu, Halil Şenol, Begüm Nurpelin Sağlık Özkan, Aydan Dag, Kadriye Benkli

**Affiliations:** † Faculty of Pharmacy, Department of Pharmaceutical Chemistry, Bezmialem Vakif University, 34093 Istanbul, Türkiye; ‡ Faculty of Pharmacy, Department of Pharmacology, Bezmialem Vakif University, 34093 Istanbul, Türkiye; § Institute of Science, Sakarya University, 34000 Sakarya, Türkiye; ∥ Faculty of Science, Department of Chemistry, Karadeniz Technical University, 61080 Trabzon, Türkiye; ⊥ Faculty of Pharmacy, Department of Pharmaceutical Chemistry, Anadolu University, 26470 Eskişehir, Türkiye; ∇ Badakbas Pharmacy, Altintepe str. Koknarli 6/C, Maltepe, 34840 Istanbul, Türkiye

## Abstract

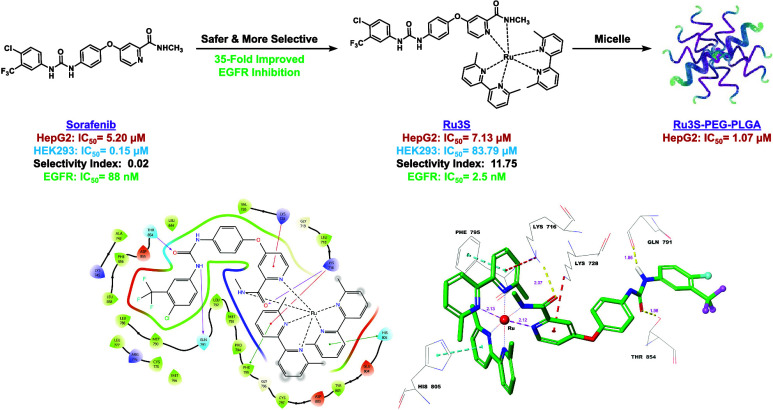

Sorafenib, a multiple kinase inhibitor, is widely used as a first-line treatment for hepatocellular carcinoma. However, there is a need for more effective alternatives when sorafenib proves insufficient. In this study, we aimed to design a structure that surpasses sorafenib’s efficacy, leading us to synthesize sorafenib–ruthenium complexes for the first time and investigate their properties. Our results indicate that the sorafenib–ruthenium complexes exhibit superior epidermal growth factor receptor (EGFR) inhibition compared to sorafenib alone. Interestingly, among these complexes, **Ru3S** demonstrated high activity against various cancer cell lines including sorafenib-resistant HepG2 cells while exhibiting significantly lower cytotoxicity than sorafenib in healthy cell lines. Further evaluation of cell cycle, cell apoptosis, and antiangiogenic effects, molecular docking, and molecular dynamics studies revealed that **Ru3S** holds great potential as a drug candidate. Additionally, when free **Ru3S** was encapsulated into polymeric micelles **M1**, enhanced cytotoxicity on HepG2 cells was observed. Collectively, these findings position **Ru3S** as a promising candidate for EGFR inhibition and warrant further exploration for drug development purposes.

## 1 Introduction

**Figure 1:**
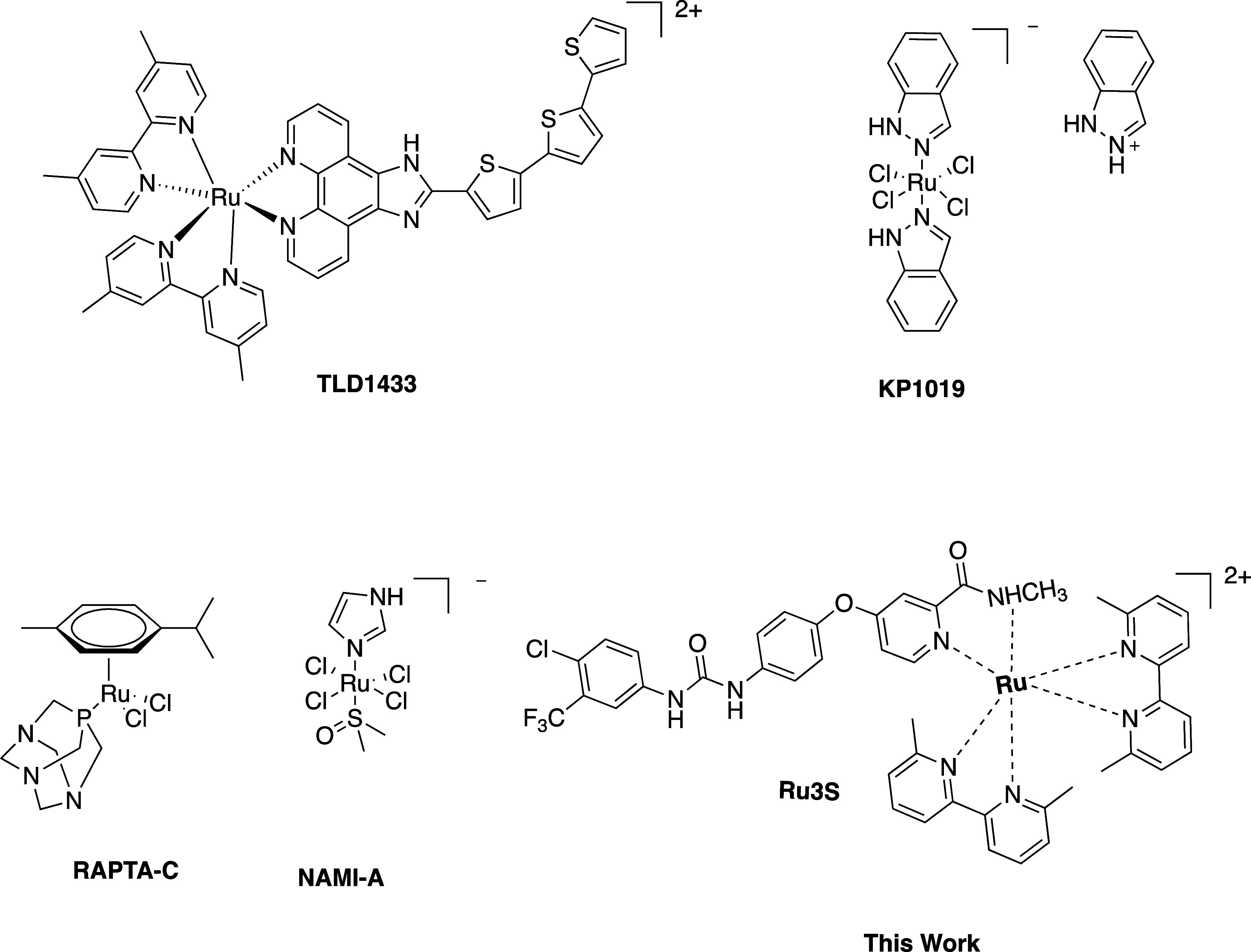
Ru complex structures in clinical trials and this work.

The transition metals exhibit the ability to bind to biomacromolecules with high specificity and enable the synthesis of complexes in different geometries thanks to their atomic properties.^1^ Coordination complexes have recently attracted attention in the scientific literature, with their unique geometries applicable to organic small-molecule-based drugs and exhibiting modifiable reactivity.^2^ Metallotherapeutics exert their effects on cancer cells through different mechanisms, such as inhibiting cancer cell division, inducing DNA damage, or disrupting the DNA repair process and triggering cancer cell apoptosis.^3^ Especially after the discovery and development of platinum complexes, their use against many tumor types has played an important role in the research of metallotherapeutics.^4,5^ Although cisplatin is one of the most progressive and widely used drugs in clinical practice, complex structures formed by metal ions other than platinum have become the focus of further research due to the serious side effects, increased drug resistance, and decreased effectiveness caused by platinum.^3,6−8^ For this purpose, the ruthenium ion is an alternative to platinum(II) because it is less toxic to healthy cells and has ligand exchange kinetics similar to Pt(II) complexes.^9−11^ In addition to these properties, ruthenium compounds are active against some cisplatin-resistant cell lines and ruthenium can mimic iron in binding to some biological molecules.^3,12,13^ Six-coordinated Ru (II/III) polypyridyl complexes exhibit good anticancer properties as they often contain a planar aromatic ring that can intercalate with DNA using DNA as a target.^14,15^ Some Ru (II/III) complexes with these structures (Figure 1), such as KP1019, NAMI-A, TLD1433, and RAPTA-C, are in clinical trials.^4,16,17^

Sorafenib is a multikinase inhibitor used against renal cell carcinoma, hepatocellular carcinoma, colorectal carcinoma, breast carcinoma, lung carcinoma, and thyroid cancer.^18^ Protein/tyrosine kinases are key enzymes that play a role in the regulation of various cellular processes that catalyze the transfer of a phosphate group from the ATP to the hydroxyl group of the target protein. Examples of tyrosine kinases (TKs) are epidermal growth factor receptor (EGFR), platelet-derived growth factor receptor (PDGFR), vascular endothelial growth factor receptor (VEGFR), fibroblast growth factor receptor (FGFR), and Src, Abl, Lck proto-oncogenes. TKs function as components of signal transduction pathways that play a central role in various biological processes such as cell growth control, metabolism, differentiation, and apoptosis. Therefore, protein kinases are important targets in anticancer drug development.^19^

Heffeter et al. conducted the first study using sorafenib and ruthenium complexes in 2013.^20^ In this study, the investigators explored the synergistic effect of KP1339 which is known to have anticancer properties and sorafenib. According to the results, when sorafenib and KP1339 were used together, they showed a cytotoxic effect that was 10-fold better than when they were used alone. In addition, this concomitant use increased apoptosis and further stopped the cell cycle in the G0/G1 phase. There are many other studies in the literature that show that ruthenium complexes can be protein kinase inhibitors.^21−25^ Also, Meggers and his group synthesized ruthenium complexes containing many different ligands and investigated the protein kinase inhibitory effects of these complexes. When the studies of the Heffeter and Meggers groups are evaluated together, it is expected that the formation of a direct Ruthenium complex, rather than a synergistic effect with sorafenib, will have a higher potential for kinase activity. In this respect, there is no study in the literature that has tested this feature.

In this study, sorafenib–ruthenium complexes were prepared for the first time using sorafenib and various ligands. Among these ligands, only the bipyridyl structure was synthesized by Yidan Lai et al. first time.^26^ In this study, the effectiveness of this complex on hepatocellular carcinoma through the photocatalytic mechanism was investigated. In our study, the effects of this and the original 7 complexes on hEGFR enzyme inhibition were investigated. We then investigated its cytotoxic effects on five different cancer cells. In order to comprehend the mechanism of enzyme inhibition, we calculated the binding energies using molecular modeling. We investigated apoptosis and the cell cycle to determine the mechanism of cell death. In addition, we examined how complexes influence the expression of tyrosine kinase pathway proteins and whether they have antiangiogenic properties. To increase the bioavailability of the obtained complexes, cytotoxic and apoptotic properties were investigated by forming micelles. With drug release studies, we demonstrated the efficacy of micelles as well. Thus, we evaluated the biological activity of our newly prepared sorafenib–ruthenium complexes, on the one hand, through tyrosine kinase enzyme inhibition, and on the other hand, we evaluated their drug release properties.

## 2 Result and Discussion

### 2.1 Chemistry

**Scheme 1:**
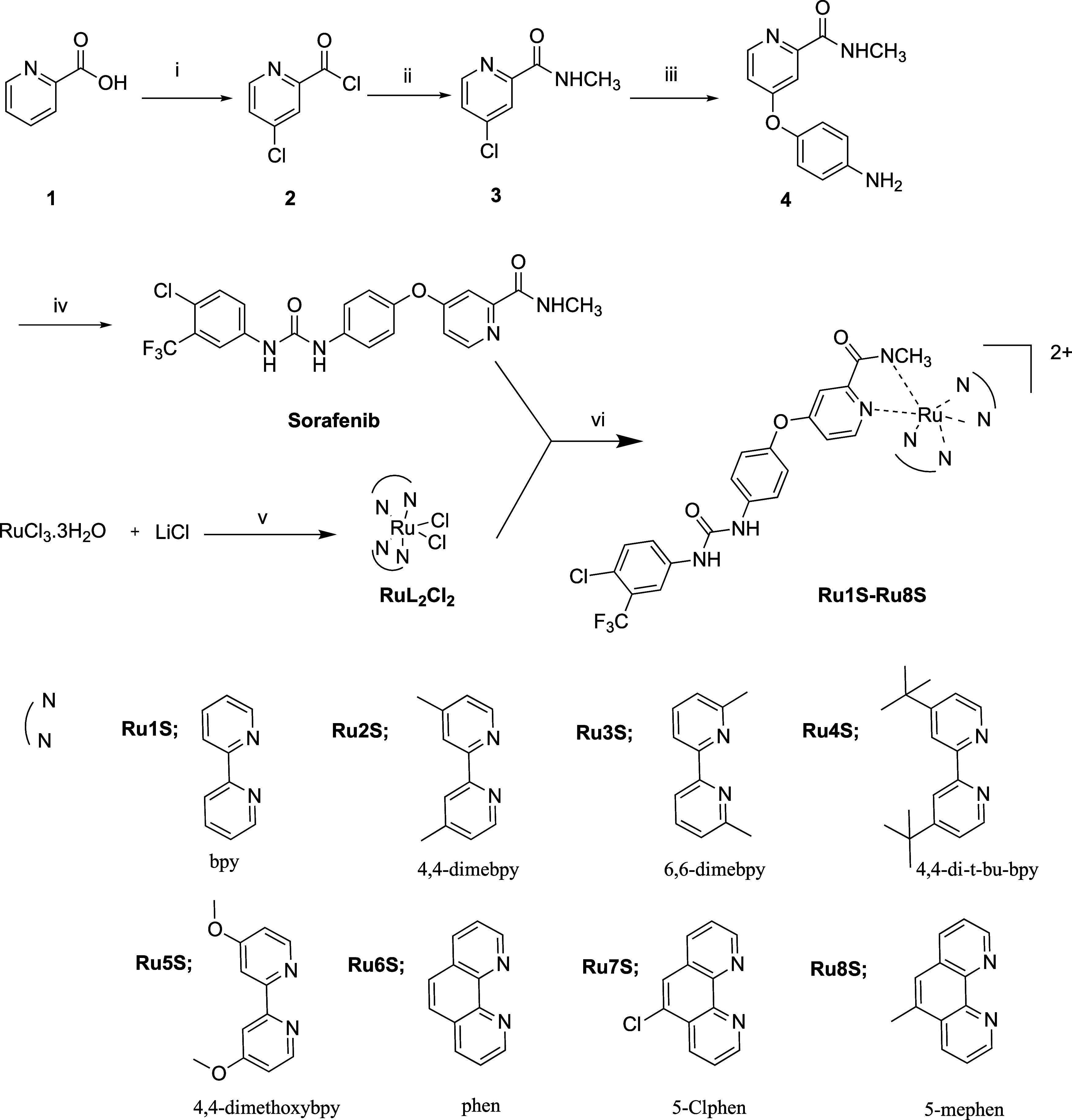
Synthesis of Sorafenib–Ruthenium Complexes (i) SOCl_2_, 72 °C, 16 h, (ii) CH_3_NH_2_, MeOH, 0 °C, (iii) *t*-BuOK, DMF, K_2_CO_3_, 80 °C, *p*-aminophenol, (iv) THF, Et_3_N, 60 °C, (v) Method A: ligand, DMF, 80 °C, N_2_, 18 h, Method B: ligand, ethylene glycol, glucose, ascorbic acid, 110 °C, 1 h, (vi) MeOH, 60 °C, 18 h.

Novel targeted ruthenium–sorafenib complexes were synthesized as shown in Scheme 1. Synthesis of sorafenib was carried out according to the method in the literature in four steps.^27−29^ The phenanthroline ring readily reacted with RuCl_3_ and LiCl in DMF^30^ to obtain ruthenium–ligand complexes (**RuL**_**2**_**Cl**_**2**_). Bipyridine and 4,4-dimethyl bipyridine structures were successfully synthesized using this method. But for substituents; when 6,6-dimethyl was changed to 4,4-dimethoxy and 4,4-di-*t*-butyl, the synthesis of these compounds by the same method was not possible. Here, an ethylene glycol–water mixture was used as a solvent, benefiting from the work of” Viala and Coudret.^31^ In this method, in which glucose and ascorbic acid are used as reducing agents, high concentrations of Cl ions were used by using an ethylene glycol–water mixture, which enabled the reaction to take place. In addition, removing the substance by heating it with dichloromethane for purification has been an effective method to remove the complex from many salts formed. At this stage, the yields of the complexes were quite high with 55–78%.

From the ^1^H NMR spectra of the obtained RuL_2_Cl_2_ complexes compared with the ^1^H NMR spectra of the ligands, it is seen that the aromatic ring signals shift to a lower area in the ^1^H NMR spectrum (Figures S1–S8). For example, the signal seen in the lowest area of the ^1^H NMR spectrum of the bipyridine (bpy) ligand is seen at 8.79 and 8.47 ppm. In the obtained Ru(bpy)_2_Cl_2_ complex, the signals in the lowest area are seen at 9.98 and 8.64 ppm. Similarly, the signal seen in the lowest area of the ^1^H NMR spectrum of the other phenanthroline (phen) ligand is at 9.77 and 8.46 ppm, while the peaks of the ruthenium complex of this ligand [Ru(phen)_2_Cl_2_] are seen at 10.28 and 8.72 ppm. In addition, in the ^1^H NMR spectra of RuL_2_Cl_2_, the signals for aromatic protons were observed between 6.92 and 10.35 ppm, while aliphatic proton signals were observed about at 1.33–4.11 ppm.

From the comparison of the ^13^C NMR spectra of the RuL_2_Cl_2_ complexes with the spectra of the ligands, it is seen that the aromatic ring carbons are similarly signaled in the lower area. In the ^13^C NMR spectrum of the bipyridine (bpy) ligand, the signals seen in the lowest area are 155.8, 149.7, and 137.7 ppm, while the ^13^C NMR spectrum of the Ru(bpy)_2_Cl_2_ complex is seen at 159.5, 157.5, 152.1, and 151.3 ppm. Similarly, in the ^13^C NMR spectrum of the phenanthroline (phen) ligand, these signals were seen at 150.4, 146.0, and 136.6 ppm, while for Ru(phen)_2_Cl_2_, these signals were seen at 154.7, 153.4, 151.2, and 149.7 ppm. In addition, the signals of the aliphatic and aromatic carbons were observed at 20–58 and 110–162 ppm, respectively (Figures S1–S8). HRMS was used to measure the mass spectra of the RuL_2_Cl_2_ complexes (Figures S9 and S10).

The results obtained were measured in accordance with the expected mass values. For example, while the mass value calculated for Ru(bpy)_2_Cl_2_ was 484.9874, the mass value found was 484.9806.

Finally, the synthesis of ruthenium–sorafenib (RuL_2_S) complexes as a result of the reaction of RuL_2_Cl_2_ and sorafenib molecule was carried out according to the method given in the literature.^32,33^ The complexes were synthesized with yields varying between 18 and 80%. Structures were characterized by ^1^H NMR and ^13^C NMR spectra. From the ^1^H NMR spectra of RuL_2_S (Figures S11–S30), the signals for aromatic protons were observed between 6.94 and 9.25 ppm, while aliphatic proton signals were observed at about 1.73–4.09 ppm. While NH protons belonging to the urea group and belonging to the amide group were not observed in the NMR measurements taken with CD_3_OD, NH protons were observed at 9.89, 10.23, and 10.92 ppm in the measurement using DMSO-*d*_6_. The specific NH–CH_3_ proton of sorafenib gives signals in the range of 2.80–2.95 ppm.

From the ^13^C NMR spectra, the signals of the aliphatic and aromatic carbons were observed at 19.8–55.9 and 113.3–172 ppm, respectively. The C=O group belonging to the amide group signals around 170 ppm, while the signals belonging to the NH–CH_3_ carbon are seen around 26.5 ppm (Figures S13 and S24). The mass spectra of these structures also gave signals in the expected range for the complexes obtained (Figures S31–S38). For example, while the mass value calculated for **Ru1S** (C_41_H_32_ClF_3_N_8_O_3_Ru [M – H]^−^) was 877.1203, the mass value found was 877.1192. In addition, the results of the elemental analysis confirm the structures of the complexes (Table S1).

It is seen that the proton integral values of **Ru7S** and **Ru8S** complexes within the RuL_2_Srf complexes are exactly twice as much as they should be in the ^1^H NMR spectra. Although flash chromatography was applied to these complexes many times, the complexes preserved the same ^1^H NMR spectra. When the elemental analysis results for these structures are examined, it is seen that all structures are compatible with the theoretical elemental analysis values. This shows that ruthenium is exactly twice as high in **Ru7S** and **Ru8S** complexes. According to these results, we can say that these two complexes contain two molecules in the same structure. In general, cis structures are expected to form in the synthesis of RuL_2_Cl_2_ complexes, but besides cis structures, trans structures can also occur. In the other complexes obtained, these trans structures were either not formed or could be removed by flash chromatography. However, it was not possible to remove the trans complex in **Ru7S** and **Ru8S** complexes. It was concluded that both cis and trans forms exist for the **Ru7S** and **Ru8S** complexes. We can say that the complexes we obtained contain a 1:1 ratio of cis and trans isomers according to ^1^H NMR integral values. When the elemental analysis results of the RuL_2_Srf complex structures are examined, it is seen that the structures that are compatible with the theoretically calculated results do not contain impurities. Based on all of these data, we can say that the complexes were successfully synthesized.

Additionally, absorption spectra were followed for 3 days to test the stability of sorafenib–ruthenium complexes (Figure S48). These results show that the sorafenib–ruthenium ligand complexes remain stable, and the results are consistent with the results found by Yidan Lai et al.^26^

### 2.2 Micelle Formation

5 mg of PEG–PLGA (50:50, MW = 5–10 kDa) and 10 mg of **Ru3S** were dissolved in 1 mL of DMSO and allowed to stir at rt. The resulting mixture was added to 4 mL ultrapure water at a rate of 0.2 mL/min with a syringe pump. It was left in the mixture overnight at 300 rpm. The next day, the unloaded drug and DMSO were removed by dialysis (MWCO: 3500) method.^34^ The mixture was stirred overnight at rt. A similar procedure was repeated to obtain **Ru4S**-loaded polymeric micelles. The size and polydispersity of micelles were determined by DLS analysis. The drug loading content (DLC) and entrapment efficiency (EE) were measured by LC-HRMS (Orbitrap Q-Exactive HRMS system (Thermo Fisher Scientific)) analysis.^35^ LC-HRMS analyses of calibration samples (**Ru3S** and **Ru4S**) were carried out as follows: A= water-1% formic acid, 0.1% ammonium formate; B= methanol-1% formic acid, 0.1% ammonium formate. The gradient elution program for **Ru3S** and **Ru4S** calibration samples was optimized and conducted as follows: 1 min. 50% B, 6 min. 100% B, 1 min. 50% B, followed by a 7 min conditioning period using a Troyasil C18 column (150 mm × 3.0 mm, 3.5 μm). The injection volume of the samples was 1 μL.

### 2.3 Biological Activity

#### 2.3.1 hEGFR Inhibition

The hEGFR enzyme inhibitions of the synthesized complexes were determined using the Takara Universal Tyrosine Kinase Assay Kit.^36^ The inhibition results obtained as a result of this experiment are given in Table 1 as the IC_50_ value, and the enzyme inhibition graphs are given in Figure S49.

**Table 1:** hEGFR Enzyme Inhibition Results of Ru–Sorafenib Complexes

			maximum inhibition	
complex	structure	IC_50_ (nM)	(%)	[I] (μM)
**Ru1S**	Ru(bpy)_2_Srf	3.9 ± 0.08	99.74	0.3
**Ru2S**	Ru(4,4-dimebpy)_2_Srf	8.1 ± 0.2	96.20	0.3
**Ru3S**	Ru(6,6-dimebpy)_2_Srf	2.5 ± 0.07	96.92	0.3
**Ru4S**	Ru(4,4-di-*t*-bu-bpy)_2_Srf	5.8 ± 0.15	96.30	0.3
**Ru5S**	Ru(4,4-dimethoxybpy)_2_Srf	1.2 ± 0.02	97.17	0.3
**Ru6S**	Ru(phen)_2_Srf	13 ± 0.1	85.41	0.3
**Ru7S**	Ru(5-Clphen)_2_Srf	0.8 ± 0.02	97.07	0.3
**Ru8S**	Ru(5-mephen)_2_Srf	1.4 ± 0.03	97.07	0.3
**Sorafenib**		88.7 ± 1.2	100	5

According to the EGFR enzyme inhibition results, all of the synthesized complexes showed higher inhibition than the sorafenib.

**Ru7S** showed the best inhibition with an IC_50_ value of 0.8 nM, and **Ru8S** and **Ru5S** complexes showed high inhibition with IC_50_ values of 1.2 nm and 1.4 nM close to this value. Other complexes also showed very high inhibition compared to the IC_50_ values of 88.7 nM concentration shown by sorafenib. Among the complexes, **Ru6S** showed the lowest inhibition with an IC_50_ value of 13 nM.

#### 2.3.2 Cell Viability against Normal and Cancer Cell Lines

The effects of the synthesized complexes on cell viability were tested by MTT assay in HepG2, Caco-2, HT-29, MCF-7, A549, and HEK293T cell lines. The IC_50_ values calculated after 48 h of substance incubation in each cell are given in Table 2. Concentration-% viability plots of each compound are presented in Figures S50–S55. According to these results, **Ru3S** was the most effective complex on HepG2 cells. Sorafenib showed a cytotoxic effect on HepG2 cells with a value of IC_50_ = 5.20 μM, while **Ru3S** exhibited a cytotoxic effect with an IC_50_ = 7.13 μM close to this value. According to the cell viability results of complexes in Caco-2 cells, the five complexes demonstrated higher cytotoxicity than sorafenib. Sorafenib showed cytotoxicity with IC_50_ = 13.80 μM, while the most effective complex **Ru4S** showed cytotoxicity with a value of IC_50_ = 2.47 μM. In addition, **Ru2S**, **Ru3S**, **Ru5S**, and **Ru6S** showed better cytotoxic effects than sorafenib in this cell line.

**Table 2:** IC_50_ (μM) Values of Synthesized Complexes Calculated from Percent (%) Viability Values in Different Cell Lines

Comp	HepG2	Caco-2	HT-29	MCF-7	A549	HEK293T
**Ru1S**	26.51 ± 10.43	18.61 ± 4.96	64.0 ± 22.62	17.18 ± 3.84	131.48 ± 22.83	169.48 ± 97.97
**Ru2S**	78.84 ± 42.81	10.98 ± 2.47	19.41 ± 5.58	17.96 ± 5.55	94.15 ± 14.76	144.43 ± 158.6
**Ru3S**	7.13 ± 2.12	9.57 ± 2.33	6.93 ± 1.76	12.09 ± 3.36	25.24 ± 2.87	83.79 ± 58.05
**Ru4S**	23.76 ± 4.23	2.47 ± 0.42	6.24 ± 1.36	5.35 ± 0.78	29.90 ± 4.48	33.15 ± 9.17
**Ru5S**	27.36 ± 5.62	11.44 ± 3.76	21.88 ± 7.19	9.47 ± 1.96	34.72 ± 4.78	87.34 ± 35.95
**Ru6S**	54.79 ± 28.65	12.42 ± 2.75	41.77 ± 11.88	19.33 ± 4.09	65.03 ± 12.40	66.15 ± 18.46
**Ru7S**	30.01 ± 6.51	17.01 ± 4.25	14.60 ± 3.46	10.11 ± 2.65	172.97 ± 57.41	764.61 ± 188
**Ru8S**	25.65 ± 4.43	20.45 ± 4.80	33.56 ± 10.17	33.07 ± 12.50	92.36 ± 19.80	16.55 ± 18.32
**Srf**	5.20 ± 1.66	13.80 ± 3.89	7.89 ± 2.53	10.55 ± 2.60	7.48 ± 0.86	0.15 ± 0.16

The **Ru4S** complex exhibited the highest cytotoxicity on the HT-29 cell line. Sorafenib showed activity with IC_50_ = 7.89 μM, while **Ru4S** was effective with IC_50_ = 6.24 μM. Furthermore, in this cell line, **Ru3S** exhibited very close cytotoxicity (IC_50_ = 6.93 μM) to that of **Ru4S (**IC_50_ = 6.24 μM). On the other hand, the **Ru4S** complex (IC_50_ = 5.35 μM) showed a higher cytotoxic effect than sorafenib (IC_50_ = 10.55 μM) in the MCF-7 cell line. In the A549 cell line, none of the complexes demonstrated higher cytotoxicity than sorafenib. However, when the complexes were compared within themselves, **Ru3S** and **Ru4S** showed the best cytotoxic properties.

When the effects of the complexes on the healthy cell line (HEK293T) were compared with sorafenib, the complexes showed low cytotoxicity on healthy cells. Sorafenib showed higher cytotoxicity on the healthy HEK293 cell line with IC_50_ = 0.15 μM, while **Ru3S** showed cytotoxicity with IC_50_ = 83.79 μM and **Ru4S** IC_50_ = 33.15 μM. The selectivity of Sorafenib and **Ru 3s** and **Ru4S** over cancer cells and healthy cells is a huge advantage for the potentials of the complexes. Since sorafenib is the first-line drug in hepatocellular carcinoma, we continued our studies with HepG2 cells.

**Figure 2:**
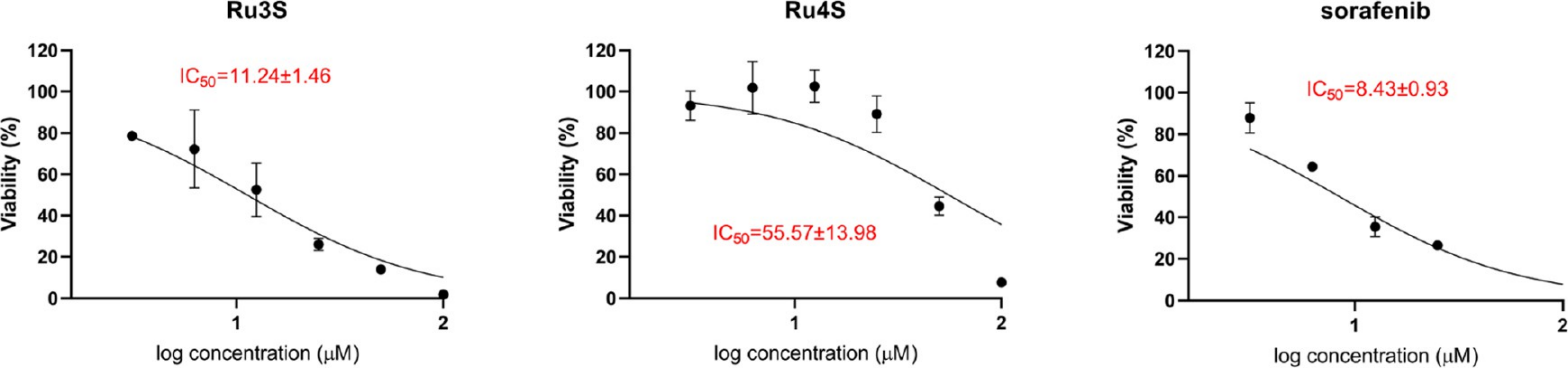
Effect of **Ru3S** and **Ru4S** on cell viability in sorafenib-resistant HepG2-SR cells.

Cytotoxic effects of **Ru3S** and **Ru4S** evaluated in sorafenib-resistant HepG2 cell line HepG2-SR. IC_50_ values of **Ru3S**, **Ru4S**, and sorafenib increased compared to the parental HepG2 cell line; however, the increment in the IC_50_ of **Ru3S** was lower compared to that of sorafenib (Figure 2).

#### 2.3.3 Cell Apoptosis and Cell Cycle

**Figure 3:**
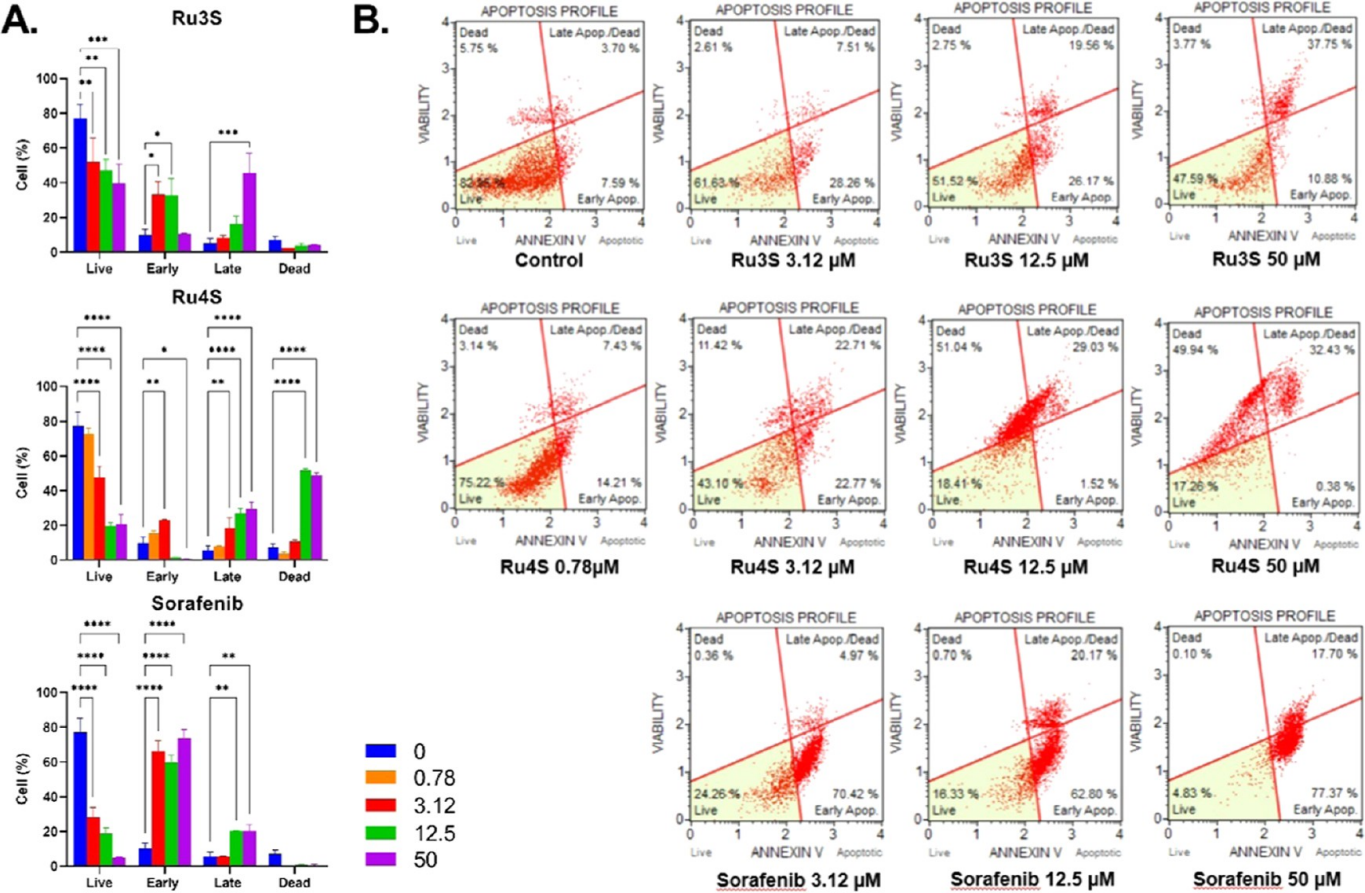
(A) Effect of concentration-dependent (0.78, 3.12, 12.5, and 50 μM) administration of **Ru3S**, **Ru4S**, and sorafenib on the percentages (%) of live, early apoptotic, late apoptotic, and dead HepG2 cells. (B) Apoptotic profiles of concentration-dependent administration of **Ru3S**, **Ru4S**, and sorafenib in HepG2 cells. The analysis of the difference between groups was conducted using a two-way analysis of variance (ANOVA) with a Dunnett post hoc test in GraphPad Prism 9.00. A significance level of *p* < 0.05 was deemed as statistically significant. *p < 0.05; **p < 0.01; ***p < 0.001; *p* < 0.0001.

The apoptotic and cell cycle properties of the **Ru3S** and **Ru4S** complexes were determined on the HepG2 cell line in the Muse Cell Analyzer. The selected **Ru3S** and **Ru4S** complexes were treated at 3 or 4 different concentrations for 48 h. At the end of this period, viable, early apoptotic, late apoptotic, and dead cell profiles for apoptosis were determined in duplicate for each compound and concentration. Cell profiles were plotted as % and statistical calculations were performed. The resulting graphs are given in Figure 3A, and the apoptotic profiles of the complexes are given in Figure 3B. Supplementary apoptotic profiles are presented in Figure S56.

According to the apoptosis results, **Ru3S** decreased the live cell percentage at all concentrations (*p* < 0.01) and increased the rate of early apoptotic cells at 3.12 and 12.5 μM concentrations (*p* < 0.05), the rate of late apoptotic cells at 50 μM concentration (*p* < 0.001). However, the rate of necrotic cells did not change depending on the concentration (*p* > 0.05). **Ru4S** showed a different profile than **Ru3S**. **Ru4S** decreased the live cell percentage at all concentrations after 0.78 μM (*p* < 0.05). The rate of early apoptotic cells increased at lower concentrations (0.78 and 3.12 μM (*p* < 0.0.5)), while at higher concentrations the proportion of late apoptotic cells (3.12, 12.5, and 50 μM) and dead cells (12.5 and 50 μM) increased compared to control (*p* < 0.0.5). Apoptotic profiles of sorafenib and **Ru3S** were similar, sorafenib decreased the live cell percentage at all concentrations (*p* < 0.0001). However, different from **Ru3S**, it increased the rate of early apoptotic cells at all concentrations (*p* < 0.0001) as well as that of late apoptotic cells at higher concentrations (12.5 and 50 μM) (*p* < 0.01). The necrotic cell percentage did not change (*p* > 0.05).

**Figure 4:**
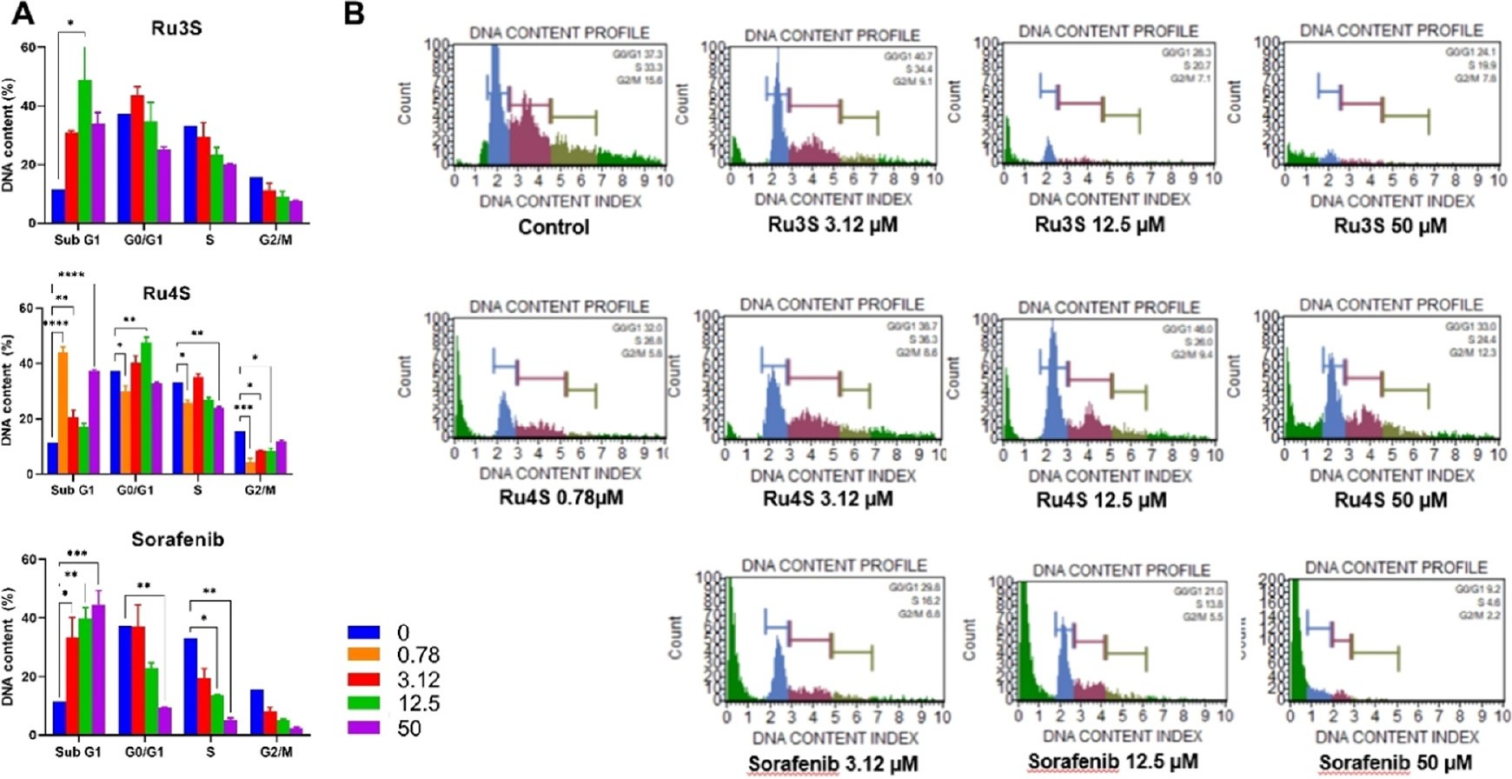
(A) Effect of concentration-dependent (0.78, 3.12, 12.5, and 50 μM) administered **Ru3S**, **Ru4S**, and sorafenib on cell cycle phases in HepG2 cells. (B) Cell cycle profiles of concentration-dependent administered **Ru3S**, **Ru4S**, and sorafenib in HepG2 cells. The analysis of the difference between groups was conducted using a two-way ANOVA with a Dunnett post hoc test in GraphPad Prism 9.00. A significance level of *p* < 0.05 was deemed as statistically significant. *p < 0.05; **p < 0.01; ***p < 0.001; *p* < 0.0001.

On the other hand, the percentage of cells in cell cycle phases for cell cycle was determined by measuring DNA content. Sub G1 (DNA content of cells in apoptotic phase), G0/G1 (Resting and protein synthesis), S (DNA synthesis), and G2/M (Protein synthesis and mitosis) phases were determined. DNA content was plotted as % and statistical calculation was made. The resulting graphs are given in Figure 4A, and the cell cycle profiles of the complexes are given in Figure 4B. Supplementary profiles are presented in Figure S57.

Dose-dependent **Ru3S** administration did not affect cell cycle phases much in HepG2 cells. It increased Sub G1 population at 12.5 μM. However, although there was a dose-dependent decrease in G0/G1, S, and G2/M, it was not statistically significant (*p* > 0.05). In contrast, **Ru4S** showed different effects on cell cycle profiles at different doses. At low concentration (0.78 μM), it increased Sub G1 (*p* < 0.0001) and decreased G0/G1, S, and G2/M percentages (*p* < 0.05). At higher concentrations (3.12 and 12.5 μM) Sub G1 was slightly increased (*p* < 0.01), G0/G1 was slightly decreased (*p* < 0.01), S phase was not changed, and G2/M was decreased (*p* < 0.05). At the highest 50 μM dose, **Ru4S** increased Sub G1(*p* < 0.0001) and decreased S (*p* < 0.01), and did not affect the G0/G1 and G2/M phases. On the other hand, sorafenib increased Sub G1 and decreased other phases similar to **Ru3S**. Sub G1 was decreased at all concentrations (*p* < 0.05), G0/G1 was decreased at 50 μM (*p* < 0.01), S phase was decreased at 12.5 and 50 μM (*p* < 0.05), and G2/M was decreased; however, it was not statistically significant (*p* > 0.05).

#### 2.3.4 Effect of Synthesized Complexes on Expression of Tyrosine Kinase Pathway Proteins

**Figure 5:**
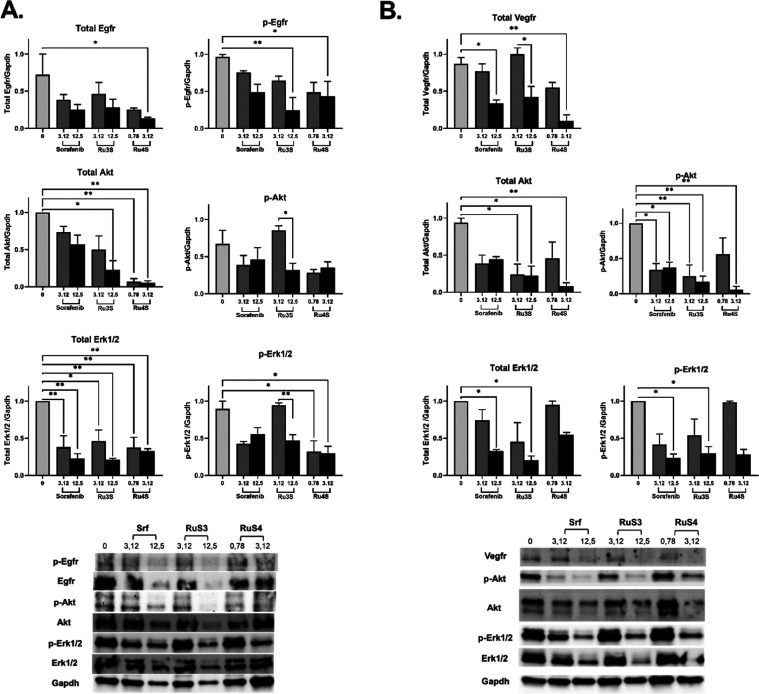
(A) Concentration (0.78 and/or 3.12 and/or 12.5 μM)-dependent effect of **Ru3S**, **Ru4S**, and sorafenib on expression of tyrosine kinase pathway proteins in HepG2 cells (B) Concentration (0.78 and/or 3.12 and/or 12.5 μM)-dependent antiangiogenic effect of **Ru3S**, **Ru4S**, and sorafenib on expression of tyrosine kinase pathway proteins in HUVEC cells. The analysis of the difference between groups was conducted using a one-way ANOVA with a Tukey post hoc test in GraphPad Prism 9.00. A significance level of *p* < 0.05 was deemed as statistically significant. *p < 0.05; **p < 0.01.*n* = 2–3.

The effects of synthesized complexes on the expression of proteins in the tyrosine kinase pathway (EGFR, p-EGFR, Akt, p-Akt, Erk1/2 (p44/42 MAPK), p-Erk1/2 (p-p44/42 MAPK)) determined by the immunoblotting method in HepG2 cells. The relative calculated densities of the band volumes for each sample normalized to the reference protein (GAPDH) and representative bands are given in Figure 5A. The difference was evaluated statistically compared to the control and between high and low concentrations.

Total EGFR decreased compared to control after **Ru4S** administration (*p* < 0.05), and p-EGFR decreased statistically in higher concentrations compared to control after **Ru3S** (*p* < 0.01) and **Ru4S** (*p* < 0.05) administration. **Ru3S** (12.5 μM; *p* < 0.05) and **Ru4S** (3.12 and 0.78; *p* < 0.01) decreased total Akt compared to control. No compound has changed the phosphorylated Akt compared to control; however, phosphorylated Akt decreased at higher concentrations of **Ru3S** compared to lower concentrations (*p* < 0.05). Total Erk1/2 decreased at lower and higher concentrations of **Ru3S**, **Ru4S**, and sorafenib compared to control (*p* < 0.05), while p-Erk1/2 decreased at higher concentrations compared to lower concentrations in **Ru3S** (*p* < 0.01) and at all concentrations of **Ru4S** compared to control (*p* < 0.05).

#### 2.3.5 Antiangiogenic Effect

The antiangiogenic effects of synthesized complexes in HUVEC (Human umbilical vein endothelial cell) cells were determined by evaluating protein expressions in the angiogenesis pathway (VEGFR, Akt, p-Akt, Erk1/2 (p44/42 MAPK), p-Erk1/2 (p-p44/42 MAPK)) by Western blot method. The relative calculated densities of the band volumes for each sample normalized to the reference protein (GAPDH) and representative bands are given in Figure 5B. The differences were evaluated statistically compared to the control and between high and low concentrations.

Total VEGFR decreased compared to control after higher concentrations of **Ru4S** and sorafenib administration (*p* < 0.05) and decreased in higher concentrations compared to lower after **Ru3S** (*p* < 0.05). Total Akt decreased after high and low concentrations of **Ru3S** and high concentrations of **Ru4S** compared to control (*p* < 0.05). However, p-Akt decreased after high and low concentrations of **Ru3S** and sorafenib, and high concentration of **Ru4S** compared to control (*p* < 0.05). Total Erk1/2 and p-Erk1/2 decreased after higher concentrations of **Ru3S** and sorafenib compared to controls (*p* < 0.05).

Upon combining the outcomes of enzyme inhibition, cell cytotoxicity, and expression of tyrosine kinase pathway proteins, it was observed that sorafenib–ruthenium ligand complexes increased enzyme inhibition by at least 6-fold compared to sorafenib alone. In addition, we have shown in this study that while sorafenib has a very high cytotoxic effect on healthy cells, sorafenib–ruthenium ligand complexes reduce this effect by at least 100-fold. We also demonstrated that ruthenium complexes down-regulate both phospho- and total Erk1/2 (MAPK1/2) in HepG2 and HUVEC cells, where Erk is up-regulated in resistant cell lines. Additionally, ruthenium complexes inhibited VEGFR expression in HUVEC cells.

A study conducted by Morgillo et al. provided evidence of a positive interaction between sorafenib and anti-EGFR drugs.^37,38^ Accordingly, they provided evidence that the combined use of sorafenib and an EGFR inhibitor in EGFR and/or VEGFR inhibitor-resistant human cancer cells is active in inhibiting tumor cell growth *in vitro* and *in vivo*. Here, the effect of sorafenib was found to be linked to its ability to block RAF signaling via the RAS/RAF/MEK/MAPK pathway. These results are consistent with our study findings. When the outcomes of our study and the study conducted by Morgillo et al. are compared, it becomes evident that our study indirectly validates the notion that the synergistic effect is amplified when the inhibition effect is employed concurrently. Because the new sorafenib–ruthenium ligand complexes we made in this study are such good at blocking EGFR, they may have helped a mechanism develop that can do this job without the need for other inhibitors. Although establishing a definitive mechanism is challenging, additional ruthenium and ligand moieties may have the effect of blocking different pathways. These mechanisms may become apparent through subsequent comprehensive investigations in the field.

#### 2.3.6 Effect of Micelles Obtained from Synthesized Complexes on Cell Viability

When the enzyme inhibition and cytotoxicity results are evaluated together, **Ru3S** and **Ru4S** showed potential as active drug substances. Therefore, the effects of these complexes were investigated on apoptosis, cell cycle, antiangiogenic effects, and proteins in the tyrosine kinase pathway. In order to increase the bioavailability of the obtained complexes we prepared micelle forms and investigated their drug release, cytotoxic, and apoptotic properties.

**Figure 6:**
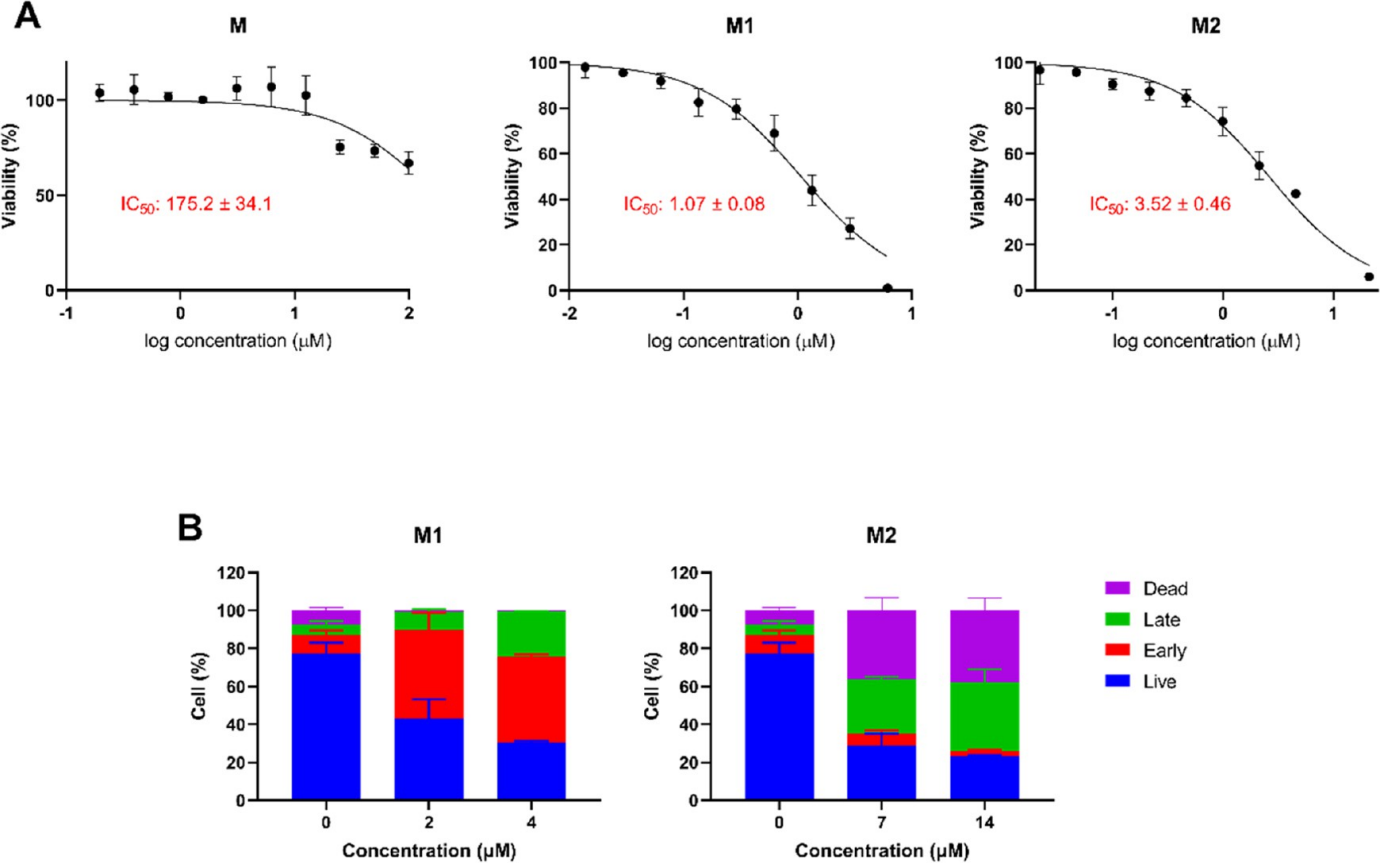
(A) Concentration–viability (%) graphics of noncomplex **M**, **Ru3S**-loaded **M1**, and **Ru4S**-loaded **M2** micelles in HepG2 cell line. (B) Concentration-dependent apoptotic cell levels of **M1** and **M2** in HepG2 cells.

The effects of **M1** and **M2** and noncomplex **M** on cell viability, respectively, obtained using **Ru3S** and **Ru4S** substances were tested by MTT assay in HepG2 cells. IC_50_ values were calculated from the percent viability values calculated according to the negative controls with the GraphPad Prism program. IC_50_ values calculated after 48 h of substance incubation are presented graphically in Figure 6A. According to the cytotoxicity results of the prepared **Ru3S**- and **Ru4S**-loaded micelles on the HepG2 cell line, noncomplex micelle **M** showed the value of IC_50_ = 175.2 μM, the **Ru3S**-loaded **M1** micelles showed IC_50_ = 1.07 μM, and the **Ru4S**-loaded **M2** micelles showed IC_50_ = 3.52 μM. These values showed that the micelle-loaded forms exhibited higher cytotoxicity when compared to the results of the complexes without loading the micelle (**Ru3S**; IC_50_ = 7.13 μM; **Ru4S**, IC_50_ = 23.76 μM).

The apoptotic properties of **M1** and **M2** were determined on the HepG2 cell line in the Muse Cell Analyzer. Viable, early apoptotic, late apoptotic, and dead cell profiles were determined for each compound at 2 concentrations and 2 replicates. The resulting graphs are presented in Figure 6B, and the profiles of the apoptotic properties of the compounds are presented in Figure S19.

According to these results, it was determined that **M1** prepared from **Ru3S** decreased the percentage of viable cells more than free **Ru3S**, despite the lower concentration. Also, **M1** increased the early apoptotic cells compared to control and free **Ru3S**. Even though **M2** decreased live cells with increasing concentrations, it increased late apoptotic and dead cells instead of early apoptotic cells as we have seen in free **Ru4S**. Both cytotoxicity and apoptosis results show that micelles prepared from the complexes increase the cytotoxic effect and the most prominent compound was **Ru3S** as well as its micelle **M1**.

### 2.4 Molecular Docking Studies

In this study, to explain the inhibition mechanisms of sorafenib–ruthenium complexes molecular docking studies of both sorafenib and its ruthenium complexes were performed and the results were compared. Molecular docking studies were performed with the induced fit docking (IFD) technique, and MM-GBSA (molecular mechanics with generalized born and surface area) Δ*G* binding free energies and glide emodel scores of the compounds against the target protein EGFR were also determined.

The induced fit docking XP (extra precision) glide scores, MM-GBSA Δ*G* binding free energies, and glide emodel scores of the ruthenium complexes as well as sorafenib against EGFR protein are given in Table 3.

**Table 3:** Molecular Docking IFD Scores and MM-GBSA Δ*G* Binding Free Energies of Ruthenium Complexes and Sorafenib

Compounds	IFD XP Gscore (kcal/mol)	MM-GBSA Δ*G* bind (kcal/mol)	glide emodel (kcal/mol)
**Ru1S**	–8.583	–66.17	–103.735
**Ru2S**	–10.554	–64.54	–100.927
**Ru3S**	–10.771	–78.51	–117.996
**Ru4S**	–7.524	–60.41	–85.799
**Ru5S**	–6.277	–69.27	–59.830
**Ru6S**	–10.163	–65.67	–85.368
**Ru7S**	–5.354	–67.30	–27.392
**Ru8S**	–9.150	–67.00	–24.458
**Sorafenib**	–9.812	–60.12	–90.643

According to the molecular docking studies, **Ru3S** exhibited the best IFD XP Gscore with −10.771 kcal/mol, indicating strong binding interaction, best MM-GBSA Δ*G* binding free energy at −78.51 kcal/mol, implying a stable binding affinity, and best Glide emodel score (−117.996 kcal/mol), suggesting a potentially strong binding mode. All scores of **Ru3S** are better than sorafenib. The IFD score, MM-GBSA Δ*G* binding free energy, and glide emodel of sorafenib are −9.812, −60.12, and −90.643 kcal/mol, respectively. In addition to **Ru3S**, the IFD scores of **Ru2S** and **Ru6S** are −10.554 and −10.163 kcal/mol, respectively, and also they are better than sorafenib. Other ruthenium complexes showed moderate and comparable docking scores, and they varied from −5.354 to −9.150 kcal/mol.

According to *in vitro* test results, the IC_50_ values of all of the ruthenium complexes against hEGFR enzyme inhibitions are at the nanomolar level. According to the results obtained from anticancer studies, the compound with the highest selectivity against healthy and cancer cells is the **Ru3S** complex. In the *in vitro* anticancer activity studies against five different cancer cell lines, the inhibitions of sorafenib and **Ru3S** are very close to each other, but the fact that the toxicity of **Ru3S** in healthy cells is much lower than sorafenib. Since **Ru3S** has better scores than all other ruthenium complexes and sorafenib in molecular docking studies, detailed molecular docking analyses of sorafenib and **Ru3S** were evaluated and the inhibition mechanisms were discussed and compared with each other.

**Figure 7:**
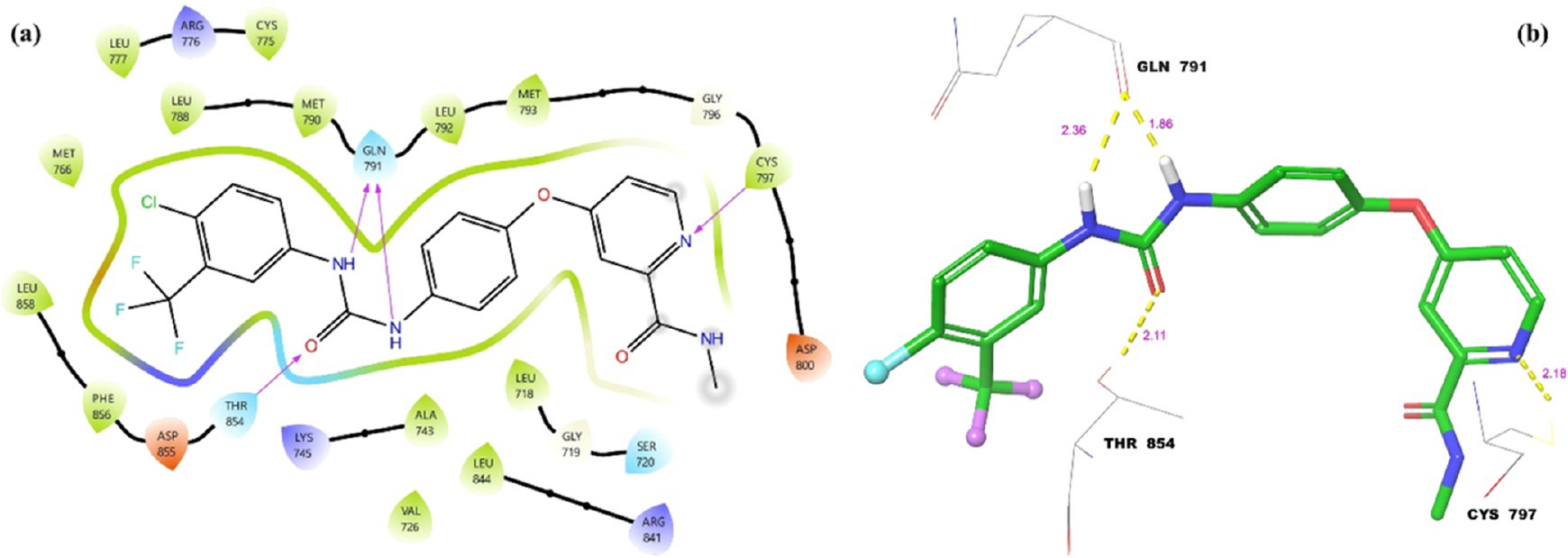
Molecular docking ligand–protein interactions between sorafenib and the active site of EGFR (PDB: 5X2A): (a) 2D ligand–protein interactions and (b) 3D ligand–protein interactions.

Molecular docking two-dimensional (2D) and three-dimensional (3D) ligand–protein interactions of sorafenib–EGFR ligand–protein complex are given in Figure 7.

As seen in Figure 7a, the oxygen of urea carbonyl formed a hydrogen-bond interaction with Thr-854. Both nitrogen of urea group formed an additional hydrogen-bond interaction with Gln-791. Finally, the nitrogen atom of the pyridyl ring also formed a hydrogen-bond interaction with Cys-797. In Figure 7b, the yellow dashes show hydrogen-bond interactions. Hydrogen-bond lengths vary from 1.86 to 2.36 Å. Key amino acids in the **Srf-EGFR** ligand–protein complex stand out as Gln-791, Thr-854, and Cys-797. Four different hydrogen bonds occurring in the active site of the enzyme indicate the stability of the **Srf-EGFR** ligand–protein complex and its inhibition ability.

**Figure 8:**
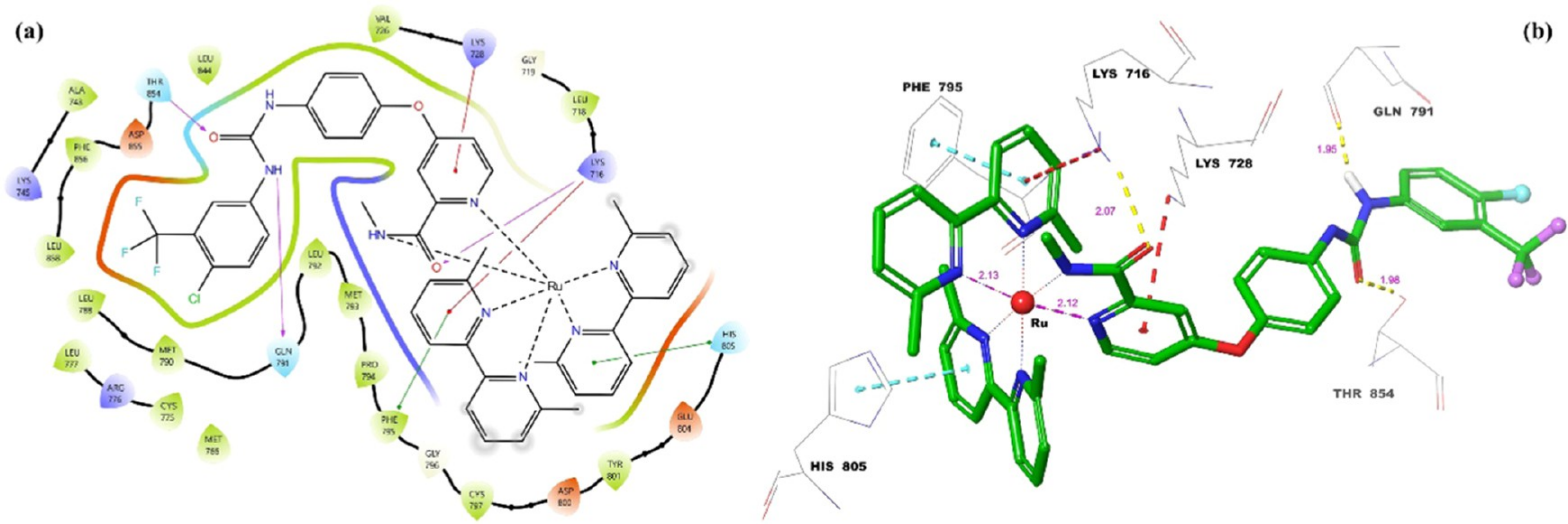
Molecular docking ligand–protein interactions between **Ru3S** and the active site of **EGFR** (PDB: 5X2A): (a) 2D ligand–protein interactions, (b) 3D ligand–protein interactions.

Molecular docking two-dimensional (2D) and three-dimensional (3D) ligand–protein interactions of **Ru3S-EGFR** ligand–protein complex are given in Figure 8. As can be seen from Figure 8a, there are three different hydrogen-bond interactions, two different π–π stacking interactions, and two different π–cationic interactions between **Ru3S** and the amino acid residues in the active site of EGFR.

When we look at the ligand–protein interactions of **Ru3S**-**EGFR** complex, it is seen that the oxygen of the urea carbonyl interacts with Thr-854, and the urea nitrogen interacts with Gln-791, as in the **Srf-EGFR** complex. Since the nitrogen atom in the pyridine ring of sorafenib is coordinated with Ru, the interaction of this nitrogen atom with Cys-797 in sorafenib is not seen in the **Ru3S-EGFR** complex. Instead, the pyridine ring of sorafenib makes a π–cationic interaction with Lys-728, while the oxygen of the amide carbonyl makes a hydrogen-bond interaction (purple) with Lys-716. Crucially, the bipyridyl rings attached to Ru engage in π–π stacking (green) and π–cationic (red) interactions with Phe-795, His-805, and Lys-716 amino acid residues in the EGFR active site. These interactions likely contribute to stabilizing the **Ru3S-EGFR** complex and disrupting the normal function of EGFR.

Figure 8b depicts the 3D ligand–protein interactions of the **Ru3S-EGFR** complex. The yellow dashes represent the hydrogen-bond interactions, the turquoise dashes represent the π–π stacking interactions, and the red dashes represent the π–cationic interactions. On the other hand, ruthenium (Ru) forms six coordination bonds with six different nitrogen atoms provided by sorafenib and the bipyridyl rings. In the **Ru3S-EGFR** complex, the hydrogen-bond lengths range from 1.95 to 2.07 Å, which closely resemble the hydrogen-bond lengths in the **Srf-EGFR** complex. Shorter hydrogen bonds signify stronger binding and a more stable ligand–protein complex. Additionally, the lengths of the Ru–N coordination bonds correlate with normal bond lengths, measured at 2.1 Å. As depicted in Figure 8b, the bipyridyl rings are positioned perpendicular to each other and to sorafenib. These ring orientations contribute to the π–cationic and π–π stacking interactions observed in the ligand–protein complex, enhancing both stability and inhibition.

When the interactions of sorafenib and **Ru3S** with EGFR are compared, they share some common features such as hydrogen-bond interactions with Thr-854 and Gln-791. However, **Ru3S** distinguishes itself by contributing unique additional interactions, specifically π–cationic and π–π stacking interactions. These distinctive features contribute to the inhibitory potential of **Ru3S** and highlight its prominence in ligand–protein stability. The additional π–cationic and π–π stacking interactions of **Ru3S** likely play a significant role in enhancing its inhibitory capacity compared to sorafenib.

Molecular docking two-dimensional (2D) and three-dimensional (3D) ligand–protein interactions of the rest of the ruthenium complex are given in the Supporting Information (Figures S62–S75).

### 2.5 Molecular Dynamics Simulations

In this study, 200 ns MD simulation analyses were carried out for **Srf-EGFR** and **Ru3S-EGFR** complexes and compared the obtained results. The findings obtained from the MD simulation of the **Srf-EGFR** complex were compared with those derived from the **Ru3S-EGFR** complex. This comparative analysis aimed to highlight the differences between the inhibitory effects demonstrated by sorafenib alone and the effects exhibited by **Ru3S**. The objective was to elucidate and outline the distinctions in the inhibition mechanisms of sorafenib and **Ru3S** when interacting with the EGFR complex.

**Figure 9:**
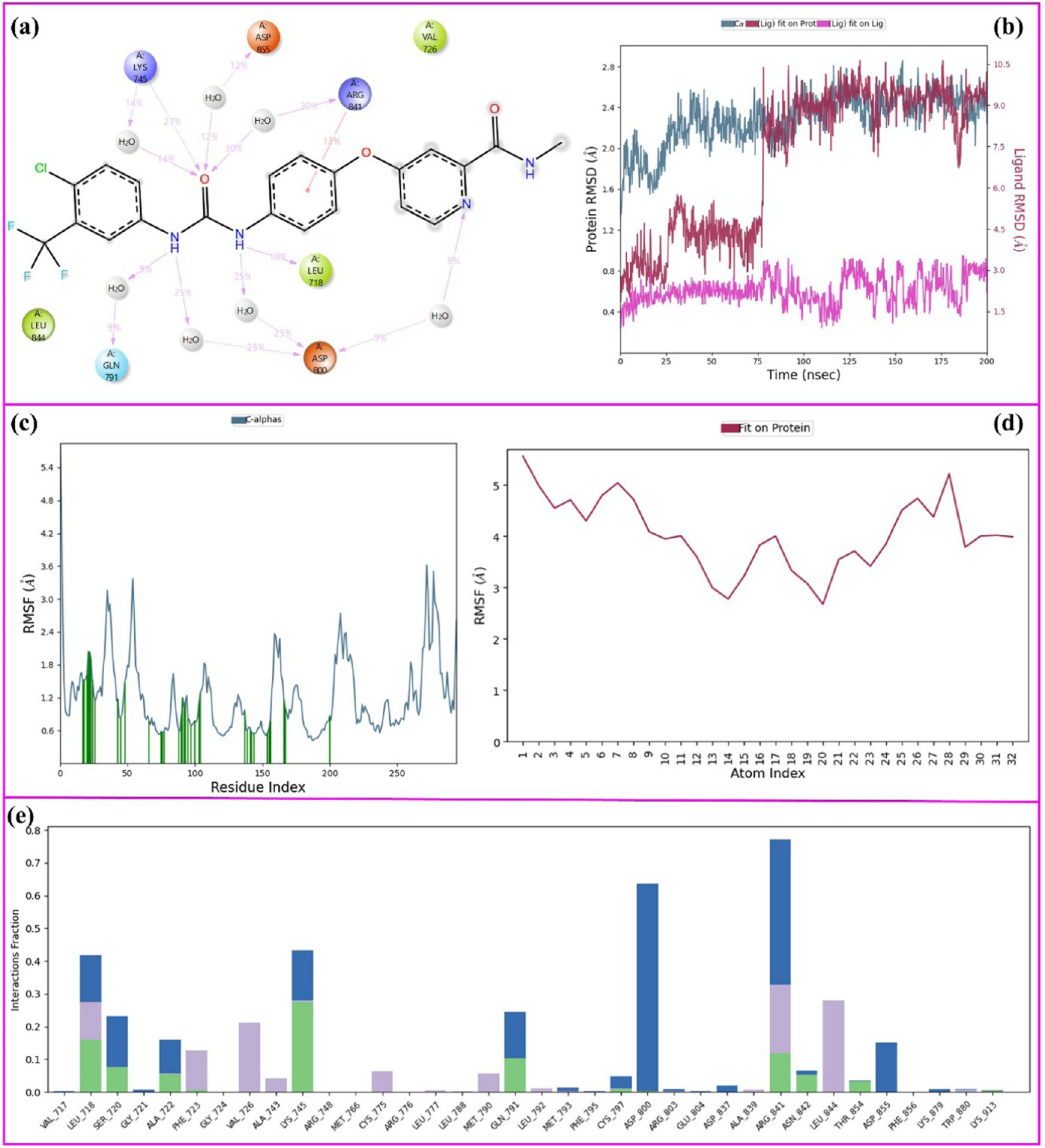
200 ns MD simulation analysis of **Sorafenib-EGFR** complex. (a) 2D key ligand–protein interactions, (b) RMSD graphics of protein and ligand atoms, (c) RMSF graphic of protein Cα atoms, (d) RMSF graphic of ligand atoms, and (e) fractional interaction histogram.

The 200 ns MD simulation analysis of the **Srf-EGFR** complex is given in Figure 9. Figure 9a represents the 2D key ligand–protein interactions with the percentage simulation time of **Srf-EGFR** MD simulations.

As seen in Figure 9a, the urea carbonyl formed a water-bridged hydrogen-bond interaction with Arg-841 (30% of the simulation time). The urea carbonyl also formed a direct hydrogen-bond interaction with Lys-745 (27% of the sim.). In addition, the urea carbonyl also formed additional two water-bridged hydrogen-bond interactions with Lys-745 (14% of the sim.) and Asp-885 (12% of the sim.). In addition to these interactions, there are several hydrogen bonds and water-bridged hydrogen-bond interactions with different percentages of the simulation time. The other most important interactions for the **Srf-EGFR** complex are the interactions occurring between urea nitrogens and Gln-791 (9% of the sim.) and Asp-800 (25% of the sim.). Although Gln-791 stood out as the key amino acid for sorafenib in molecular docking studies, its interaction in MD simulations continued for only 9% of the simulation time. In addition, the amino acid residue Asp-800 showed multiple water-bridged hydrogen-bond interactions throughout the simulation. Finally, Arg-841 shoved a π–cationic interaction with the *p*-substituted phenyl ring during 13% of the simulation time.

According to Figure 9b, the average RMSD of the protein Cα was calculated as 2.7 Å, the average RMSD of ligand atoms was calculated as 7.50 Å, and the average ligand deviation from its original position was 2.4 Å. While the RMSD of the ligand atoms at 4.5 Å is almost half of the simulation, it stabilized at 9 Å levels in the second half of the simulation. Since 9 Å is a high value for the ligand RMSD, the stability of the ligand–protein complex is thought to be low. Figure 9c,d represents the RMSF values of the protein Cα atoms and ligand atoms, respectively. As seen in Figure 9c,d, the average RMSF of the protein Cα and ligand atoms were calculated as 1.8 and 4 Å, respectively. In Figure 9c, the vertical green bars represent the ligand-amino acid contacts during the simulation time.

Finally, Figure 9e represents the fractional interaction histograms of the **Srf-EGFR** complex. Throughout the simulation, interactions like hydrogen bonds and hydrophobic, ionic, and water bridges were continuously monitored and categorized. The stacked bar charts in Figure 9e efficiently show how often these interactions occur, with values like 0.8 indicating an 80% presence. Values over 1.0 suggest instances where specific protein residues engage with the ligand multiple times in the same category. The most abundant fractional interactions were observed with Arg-841, Asp-800, Leu-718, Lys-845, and Gln-791. These amino acid residues are the key amino acids that showed multiple interactions during the simulation.

**Figure 10:**
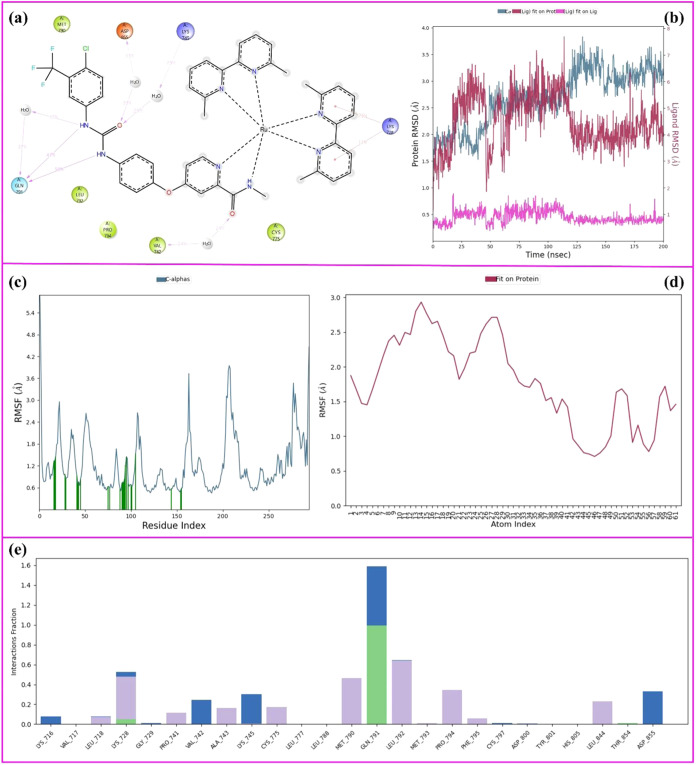
200 ns MD simulation analysis of **Ru3S-EGFR** complex. (a) 2D key ligand–protein interactions, (b) RMSD graphics of protein and ligand atoms, (c) RMSF graphic of protein Cα atoms, (d) RMSF graphic of ligand atoms, and (e) fractional interaction histogram.

The 200 ns MD simulation analysis of the **Ru3S-EGFR** complex is given in Figure 10. Figure 10a represents the 2D key ligand–protein interactions with the percentage simulation time of **Ru3S-EGFR** MD simulations.

As seen in Figure 10a, the urea nitrogens formed two hydrogen-bond interactions with Gln-791 (47 and 50% of the sim), and a water-bridged hydrogen-bond interaction with Gln-791 (37% of the sim.). On the other hand, the urea carbonyl formed two different water-bridged hydrogen-bond interactions with Asp-855 (33% of the sim.) and Lys-745 (29% of the sim.). In addition, the amid carbonyl formed an additional water-bridged hydrogen-bond interaction with Val-742 (24% of the sim.) and the bipyridyl ring of the Ru complex formed two π–cationic bond interactions with Lys-728. For the Gln-791 key amino acid residue, which stands out in molecular docking studies for both ligands, the interaction time in the **Srf-EGFR** complex is 9% of the simulation, while this time is 50% in the **Ru3S-EGFR** complex. This result indicates that the **Ru3S** forms a stable complex throughout the simulation because binding to a specific amino acid residue and remaining bound for a long time is better than short-term interactions with more than one amino acid residue. Moreover, π–cationic interactions of bipyridyl rings attached to the ruthenium complex contribute to the stability of the ligand–protein complex.

Figure 10b represents the RMSD values of the **Ru3S-EGFR** complex. According to Figure 10b, the average RMSD of the protein Cα atoms was calculated as 2.8 Å (pale blue), the average RMSD of the ligand atoms was calculated as 4.5 Å, and the average deviation of the ligand from its original position is 1 Å. Comparing the RMSD values, the **Ru3S-EGFR** complex appears more stable than the **Srf-EGFR** complex. In **Ru3S-EGFR**, the ligand shows a lower average RMSD (4.5 Å) and a smaller deviation from its original position (1 Å) compared to **Srf-EGFR** (ligand RMSD of 7.50 Å and a deviation of 2.4 Å). These results suggest that **Ru3S** maintains a more consistent position within the binding site, indicating greater stability during the simulations.

Figure 10c,d represents the RMSF plots of the protein Cα atoms and ligand atoms of **Ru3S-EGRF** complex, respectively. The average RMSF of the protein Cα was calculated as 1.8 Å, and this value is the same as the value in the **Srf-EGFR** complex. The protein backbones have the same stability and fluctuation in both complexes. On the other hand, the RMSF of the ligand atoms was calculated to be 1.5 Å on average, and this value is much lower than that of the **Srf-EGFR** complex. The ruthenium complex created a more stable ligand–protein complex by restricting the fluctuation of the molecule throughout the simulation. Finally, Figure 10e represents the fractional interaction histogram of the **Ru3S-EGFR** complex during the 200 ns simulation time. The most abundant fractional histograms were observed with Gln-791, Leu-792, Met-790, and Lys-728. Actually, the abundance of the Gln-791 fractions is so high and Gln-791 stands out as the key amino acid residue for the inhibition.

In conclusion, the 200 ns MD simulations of **Srf-EGFR** and **Ru3S-EGFR** complexes revealed distinct dynamic behaviors. In **Srf-EGFR**, diverse interactions were observed with varying durations, resulting in ligand fluctuations (average RMSD of 7.50 Å). Conversely, **Ru3S-EGFR** exhibited enhanced stability, demonstrated by sustained hydrogen-bond interactions, lower ligand RMSD (4.5 Å), and a smaller deviation (1 Å). The π–cationic interactions of the bipyridyl ring in **Ru3S** contributed to its stability. Both complexes highlighted Gln-791 as a key amino acid, but **Ru3S** exhibited more abundant and consistent interactions, making it a potentially more stable and promising candidate for drug development. The RMSF analysis indicated a more rigid binding in **Ru3S-EGFR**, emphasizing the advantages of **Ru3S** in forming a stable ligand–protein complex.

### 2.6 Micelle Formation and Drug Release Studies

The size of **Ru3S**- and **Ru4S**-loaded PEG–PLGA polymeric micelles was performed with DLS analysis. **M1** and **M2** refer to **Ru3S**- and **Ru4S**-loaded polymeric micelles, respectively. The sizes of micelles were 99.7 ± 0.9 nm with a PDI value of 0.22 for **M1** and 67.7 ± 0.7 nm with a PDI value of 0.23 for **M2**. The amount of **Ru3S** and **Ru4S** loaded into the micelles was determined by LC-HRMS analysis. A certain amount of freeze-dried **M1** and **M2** micelles was dissolved in DMF, and the LC-HRMS was carried out. The drug loading content (DLC) and entrapment efficiency (EE) were determined according to the equations in Figure S60. According to these equations, %DLC of 7.81% and %EE of 15.6% were determined for **Ru3S**-loaded micelles (M1). For **Ru4S**-loaded micelles (M2), %DLC and %EE were determined as 31.25 and 62.5%, respectively.

To investigate the controlled release properties of polymeric micelles, the prepared **M2** nanocarrier was dispersed in two buffer solutions at different pH values (7.4 and 5.6) at 37 °C.

The release profile of **Ru4S** from **M2** was determined by LC-HRMS analysis. As expected, only a very small amount (less than 25%) of the **Ru4S** was released from **M2** in a neutral medium (pH = 7.4) even after 24 h. However, in a weakly acidic environment (pH = 5.5), the release was 80% as shown in Figure S61C. At pH values of 7.4 and 5.5, release efficiencies of 34 and 93% were achieved after 96 h, respectively (Figure S61).

## 3 Conclusions

Sorafenib is a well-known tyrosine kinase inhibitor, but the need for the discovery of new molecules still remains. This study demonstrates that these new sorafenib–ruthenium complexes inhibit the cellular proliferation of hepatocellular carcinoma cells and show EGFR enzyme inhibition. Eight new sorafenib–ruthenium complexes were synthesized and EGFR enzyme inhibition was investigated. All complexes showed better nanomolar activity than sorafenib. **Ru6S** demonstrated weaker inhibition compared to other complexes with IC_50_ values of 13 nM, whereas **Ru7S** showed the highest inhibitory effect against hEGFR with IC_50_ values of 0.8 nM. When compared to sorafenib (IC_50_ = 88 nM), all complexes displayed extremely strong inhibition.

HepG2, Caco-2, HT-29, MCF-7, and A549 are five distinct cancer cell lines used to test the cytotoxicity effects of the complexes. According to the cytotoxicity results of the complexes, it was found that they exhibit different cytotoxicity for each cell. Considering its selective effects on healthy and cancer lines, further research on HepG2 cells was continued. Among all of the complexes, **Ru3S** demonstrated a higher antiproliferative effect on the HepG2 cell line as well as sorafenib-resistant HepG2-SR cells. Additionally, **Ru3S** showed lower cytotoxicity on the healthy HEK293T cell line compared to sorafenib, which is a desirable feature for a drug candidate.

The effects of **Ru3S** and **Ru4S** complexes on the apoptotic and cell cycle properties of the HepG2 cell line were distinct. **Ru3S** and sorafenib increased Sub G1 and decreased other phases of the cell cycle, whereas various doses of **Ru4S** had varying effects on the cell cycle profiles. In HepG2 cells, immunoblotting revealed that the complexes decreased the expression of total EGFR, p-EGFR, total Akt, p-Akt, total Erk1/2, and p-Erk1/2 relative to the control, with higher concentrations having a greater effect than lower concentrations. The synthesized complexes had antiangiogenic effects on HUVEC cells, as indicated by a decrease in angiogenesis pathway protein expression. VEGFR, Akt, p-Akt, Erk1/2, and p-Erk1/2 all decreased following administration of **Ru3S**, **Ru4S**, and sorafenib compared to the control and lower concentrations. These findings indicate that the **Ru3S** and **Ru4S** complexes have the potential to be used as anticancer agents.

The encapsulation of free drugs (**Ru3S** and **Ru4S**) into polymeric micelles (**M1** and **M2**) resulted in an increase in cytotoxicity of the drug on the HepG2 cells. Thus, polymeric micelles have the potential to be carriers of the hydrophobic drugs of **Ru3S** and **Ru4S**.

In conclusion, the cytotoxicity and apoptosis experiments revealed that the micelles prepared from the complexes increased the cytotoxic effect, with **Ru3S** and its micelle **M1** being the most prominent compound.

Finally, to determine ligand–protein interactions and complex stability as well as to evaluate the inhibition mechanism of the ruthenium complexes, molecular docking and dynamics studies were carried out and compared with sorafenib. Docking studies revealed that **Ru3S** shows the best docking score, MM-GBSA Δ*G* binding free energy, and glide emodel scores. In addition, MD simulations showed that the **Ru3S-EGFR** complex is more stable than the **Srf-EGFR** complex. Molecular docking and dynamics studies confirmed **Ru3S** as a promising candidate, revealing favorable binding interactions and enhanced stability in comparison to sorafenib against EGFR.

## 4 Experimental Section

### 4.1 Materials

The chemicals and solvents were bought from Fluka Chemie, Merck, Alfa Aesar, and Sigma-Aldrich. Melting points were determined on a STUART SMP40. IR spectra were measured on an Alfa Bruker spectrometer. ^1^H and ^13^C NMR spectra were acquired on a Bruker spectrometer at 500 and 125 Hz, respectively. Mass spectra were obtained using a Thermo Fisher Scientific LC-HRMS spectrometer. UV measurements were determined with a HITACHI U-2900 brand spectrometer. Spectrophotometric analyses were performed by a BioTek Power Wave XS (BioTek). The cell line was purchased from American Type Culture Collection (ATCC). Dulbecco’s modified Eagle’s medium-F12, RPMI medium, fetal calf serum, and PBS were bought from GIBCO BRL, Invitrogen (Carlsbad, CA). All compounds used for biological assays were >95% pure, as determined by HPLC analysis (Figures S39–S47).

### 4.2 Methods

#### 4.2.1 General Procedures and Spectral Data

##### 4.2.1.1 Synthesis of Sorafenib

4-Chloropicolinoyl chloride (**2**): 0.08 mol of thionyl chloride and 1 mL of DMF were mixed under nitrogen at 40 °C for 10 min. 0.4 mol of picolinic acid (**1**) was added in half an hour piece by piece. When the addition was complete, the temperature was increased to 72 °C and stirred at this temperature for 16 h. A yellow precipitate formed at the end of the reaction. The resulting solid was purified with the help of toluene and ether to obtain 4-chloropicolinoyl chloride (**2**) in 60% yield.^27^^1^H NMR (DMSO-*d*_6_, 300 MHz) δ/ppm: 8.62 (1H, d, *J* = 7.4 Hz), 8.10 (1H, s), 7.46 (1H, d, *J* = 7.4 Hz).

4-Chloro-*N*-methylpicolinamide (**3**): 4-Chloropicolinoyl chloride (**2**) and methyl amine were mixed in cold methanol. The solid part formed at the end of the reaction was dried by filtration through the gooch crucible. 4-Chloro-*N*-methylpicolinamide (**3**) obtained in 20% yield was used for the next step.^27^^1^H NMR (DMSO-*d*_6_, 500 MHz) δ/ppm: 8.72 (1H, d, *J* = 7.3 Hz), 8.14 (1H, s), 7.52 (1H, d, *J* = 7.3 Hz), 2.59 (3H, d, *J =* 4.5 Hz).

4-(4-Aminophenoxy)-*N*-methylpicolinamide (**4**): First, *p*-aminophenol and *t*-BuOK were mixed in DMF for 2 h under nitrogen, then compound **3** and K_2_CO_3_ were added and mixed at 80 °C for 16 h. At the end of the reaction, DMF was evaporated in the evaporator. 4-(4-Aminophenoxy)-*N*-methylpicolinamide (**4**) was obtained by column chromatography with hexane-ethyl acetate.^28^^1^H NMR (DMSO-*d*_6_, 500 MHz) δ/ppm: 2.79 (3H, d, *J =* 4.8 Hz), 5.22 (2H, s, NH_2_), 6.65 (2H, d, *J =* 8.7 Hz), 6.87 (2H, d, *J =* 8.7 Hz), 7.08 (1H, dd, *J =* 5.6, 2.6 Hz), 7.35 (1H, d, *J =* 2.5 Hz), 8.46 (1H, d, *J =* 5.62 Hz), 8.78 (1H, q, *J =* 4.5 Hz); ^13^C NMR (DMSO-*d*_6_, 125 MHz) δ/ppm: 26.4, 108.7, 114.1, 115.3, 122.0, 143.2, 147.3, 150.6, 152.7, 164.3, 167.2.

4-(4-(3-(4-Chloro-3-(trifluoromethyl)phenyl)ureido)phenoxy)-*N*-methylpicolinamide (**Sorafenib**): 1 mmol of 4-(4-aminophenoxy)-*N*-methylpicolinamide (**4**), 1.1 mmol of 4-chloro-3-trifluoromethyl phenyl isocyanate, and 1.1 mmol of Et_3_N were dissolved in THF together and stirred overnight at 60 °C. At the end of the reaction, THF was evaporated in the evaporator. The solid was purified by washing with ether.^29^^1^H NMR (DMSO-*d*_6_, 500 MHz) δ/ppm 2.81 (3H, d, *J =* 4.8 Hz), 7.29–7.06 (3H m), 7.41 (1H, d, *J =* 2.5 Hz), 7.78–7.56 (4H, m), 8.15 (1H, d, *J =* 2.5 Hz), 8.52 (1H, d, *J =* 5.6 Hz), 8.82 (1H, q, *J =* 4.7, 4.6 Hz), 9.04 (1H, s), 9.26 (1H, s); ^13^C NMR (DMSO-*d*_6_, 125 MHz) δ/ppm: 26.4, 109.0, 114.4, 117.2, 120.9, 121.9, 122.8, 123.5, 124.3, 127.0, 132.4, 137.5, 139.7, 148.2, 150.8, 152.8, 152.9, 164.2, 166.4.

##### 4.2.1.2 Synthesis of RuL_2_Cl_2_ Complexes

Method A: The mixture of RuCl_3_·(H_2_O)*n* (1 mmol), ligand (L) (2 mmol), LiCl (6 mmol), and DMF (2 mL) was stirred under nitrogen at 80 °C under reflux for 18 h. At the end of the reaction, acetone was added after cooling and the mixture was kept in a refrigerator for 24 h. The precipitate formed was filtered, washed with cold water and diethyl ether, and then dried. The resulting complex was purified by column chromatography.^30^ The following complexes were synthesized according to this method.

Ru(bpy)_2_Cl_2_: Dark purple powder, 60% yield, 360 °C (decomposition) mp. ^1^H NMR (DMSO-*d*_6_, 500 MHz) δ/ppm; 9.98 (d, *J =* 5.4 Hz, 1H), 8.64 (d, *J =* 8.0 Hz, 1H), 8.48 (d, *J =* 8.0 Hz, 1H), 8.12–8.01 (m, 1H), 7.77 (t, *J =* 6.4 Hz, 1H), 7.74–7.63 (m, 1H), 7.51 (d, *J =* 5.5 Hz, 1H), 7.10 (dd, *J =* 9.6, 3.4 Hz, 1H); ^13^C NMR (DMSO-*d*_6_, 125 MHz) δ/ppm; 159.5, 157.5, 152.5, 151.3, 133.9, 132.6, 124.7, 124.6, 122.2, 121.8. HRMS; calcd for C_20_H_16_Cl_2_N_4_Ru [M + H]^+^; 484.9874, found; 484.9806.

Ru(4,4-dimebpy)_2_Cl_2_: Dark purple powder, 46% yield, 360 °C (decomposition) mp. ^1^H NMR (DMSO-*d*_6_, 500 MHz) δ/ppm; 9.77 (d, *J =* 5.7 Hz, 1H), 8.46 (s, 1H), 8.31 (s, 1H), 7.58 (d, *J =* 5.0 Hz, 1H), 7.31 (d, *J =* 5.9 Hz, 1H), 6.92 (dd, *J =* 5.9, 1.1 Hz, 1H), 2.62 (s, 3H), 2.34 (s, 3H). ^13^C NMR (DMSO-*d*_6_, 125 MHz) δ/ppm; 160.2, 158.4, 152.9, 151.6, 145.9, 144.7, 126.5, 126.5, 123.8, 123.4, 21.1, 20.7. HRMS; calcd for C_24_H_24_Cl_2_N_4_Ru [M + H]^+^; 541.0500, found; 541.1046.

Ru(phen)_2_Cl_2_: Dark purple powder, 56% yield, 370 °C (decomposition) mp. ^1^H NMR (DMSO-*d*_6_, 500 MHz) δ/ppm; 10.28 (dd, *J =* 5.2, 1.2 Hz, 1H), 8.72 (dd, *J =* 8.1, 1.2 Hz, 1H), 8.29 (d, *J =* 8.8 Hz, 1H), 8.26–8.20 (m, 2H), 8.14 (d, *J =* 8.8 Hz, 1H), 7.75 (dd, *J =* 5.4, 1.0 Hz, 1H), 7.33 (dd, *J =* 8.0, 5.4 Hz, 1H); ^13^C NMR (DMSO-*d*_6_, 125 MHz) δ/ppm; 154.7, 153.4, 151.2, 149.7, 134.0, 132.5, 130.0, 127.9, 127.5, 125.2, 124.9, 123.9. HRMS; calcd for C_24_H_16_Cl_2_N_4_Ru [M + H]^+^; 532.9874, found; 532.1806.

Ru(5-Clphen)_2_Cl_2_: Dark purple powder, 29% yield, 375 °C mp. ^1^H NMR (DMSO-*d*_6_, 500 MHz) δ/ppm; 10.35 (dt, *J =* 5.2, 1.1 Hz, 1H), 10.26 (td, *J =* 5.2, 1.2 Hz, 1H), 8.83 (dd, *J =* 8.4, 2.5 Hz, 1H), 8.68 (dt, *J =* 7.6, 1.3 Hz, 1H), 8.62 (d, *J =* 2.01 Hz, 1H), 8.47 (s, 1H), 8.40–8.28 (m, 2H), 8.28–8.20 (m, 2H), 7.98–7.88 (m, 1H), 7.85 (dd, *J =* 5.4, 1.0 Hz, 1H), 7.47–7.43 (m, 1H), 7.38–7.34 (m, 1H); ^13^C NMR (DMSO-*d*_6_, 125 MHz) δ/ppm; 161.7, 154.5, 154.0, 153.7, 150.6, 149.2, 149.2, 147.9, 132.5, 131.1, 129.9, 128.9, 128.4, 127.1, 126.0, 125.7, 125.0, 124.8, 124.6, 124.4. HRMS; calcd for C_24_H_14_Cl_4_N_4_Ru [M + H]^+^; 600.9094, found; 600.9550.

Ru(5-mephen)_2_Cl_2_: Dark purple powder, 55% yield, 373 °C mp. ^1^H NMR (DMSO-*d*_6_, 500 MHz) δ/ppm; 10.29 (d, *J =* 4.0 Hz, 1H), 10.20 (s, 1H), 8.74 (d, *J =* 8.2 Hz, 1H), 8.58 (d, *J =* 7.9 Hz, 1H), 8.24–8.20 (m, 2H), 8.17–8.13 (m, 1H), 8.09 (d, *J =* 4.4 Hz, 2H), 7.95 (d, *J =* 6.7 Hz, 1H), 7.76–7.68 (m, 1H), 7.67–7.60 (m, 1H), 7.35 (dd, *J =* 8.1, 5.4 Hz, 1H), 7.28 (dd, *J =* 7.8, 5.4 Hz, 1H), 2.76 (s, 3H), 2.89 (s, 3H); ^13^C NMR (DMSO-*d*_6_, 125 MHz) δ/ppm; 171.4, 153.1, 152.5, 149.6, 148.8, 147.9, 138.2, 134.4, 134.1, 132.0, 130.8, 130.1, 129.1, 129.0, 128.6, 128.5, 125.4, 125.1, 124.2, 123.9, 123.7, 123.5, 114.0, 21.4, 20.4. HRMS; calcd for C_26_H_20_Cl_2_N_4_Ru [M + H]^+^; 561.0187, found; 561.0657.

Method B: RuCl_3_·(H_2_O)*n* (1 mmol) and LiCl (40 mmol) were mixed in ethylene glycol:water mixture at 110 °C for 15 min. After 15 min, ligand (2.2 mmol) was added and mixing was continued. Then, glucose (2 mmol) was added and mixed for another 15 min at 110 °C. Ascorbic acid was added to it and mixing was continued for 30 min. After 30 min, 10 mL of saturated NaCl solution was added and stirred at 0 °C for 1 h. The precipitate was then filtered off and purified by column chromatography.^31,39^ The following complexes were synthesized according to this method.

Ru(6,6-dimebpy)_2_Cl_2_: Dark purple powder, 78% yield, 370 °C (decomposition) mp. ^1^H NMR (DMSO-*d*_6_, 500 MHz) δ/ppm; 8.58 (d, *J =* 7.8 Hz, 1H), 8.44 (d, *J =* 7.7 Hz, 2H), 8.36 (d, *J =* 7.7 Hz, 1H), 8.06 (t, *J =* 7.5 Hz, 1H), 7.95 (t, *J =* 7.6 Hz, 1H), 7.85 (t, *J =* 7.6 Hz, 1H), 7.78 (d, *J =* 8.4 Hz, 1H), 7.55 (d, *J =* 7.4 Hz, 1H), 7.48 (d, *J =* 7.4 Hz, 1H), 7.28–7.23 (m, 2H), 2.91 (s, 3H), 2.53 (s, 3H), 1.86 (s, 3H), 1.65 (s, 3H); ^13^C NMR (DMSO-*d*_6_, 125 MHz) δ/ppm; 167.9, 166.8, 166.5, 164.9, 163.9, 161.9, 161.1, 160.5, 137.1, 136.5, 135.8, 135.2, 124.6, 124.6, 123.9, 123.675, 121.6, 121.0, 120.7, 120.6, 27.2, 24.7, 24.6, 23.7. HRMS; calcd for C_24_H_24_Cl_2_N_4_Ru [M + H]^+^; 541.0500, found; 541.1334.

Ru(4,4-dimethoxybpy)_2_Cl_2_: Dark purple powder, 50% yield, 370 °C mp. ^1^H NMR (DMSO-*d*_6_, 500 MHz) δ/ppm; 9.83 (d, *J =* 6.6 Hz, 1H), 9.20 (d, *J =* 6.5 Hz, 1H), 8.46 (d, *J =* 2.7 Hz, 1H), 8.36 (d, *J =* 2.6 Hz, 1H), 8.30 (dd, *J =* 8.2, 2.6 Hz, 2H), 7.59 (dd, *J =* 6.6, 2.6 Hz, 1H), 7.51 (d, *J =* 6.6 Hz, 1H), 7.47 (dd, *J =* 6.6, 2.7 Hz, 1H), 7.07 (dd, *J =* 6.5, 2.5 Hz, 1H), 7.03–7.00 (m, 2H), 4.11 (s, 3H), 4.08 (s, 3H), 3.94 (s, 3H), 3.92 (s, 3H); ^13^C NMR (DMSO-*d*_6_, 125 MHz) δ/ppm; 167.5, 167.3, 166.9, 166.7, 159.2, 159.0, 158.8, 157.5, 156.4, 154.0, 153.4, 150.1, 114.6, 114.1, 113.5, 113.2, 112.0, 111.6, 111.2, 110.4, 57.4, 57.2, 57.2, 57.1. HRMS; calcd for C_24_H_24_Cl_2_N_4_O_4_Ru [M + H]^+^; 605.0296, found; 605.0755.

Ru(4,4-di-*t*-bu-bpy)_2_Cl_2_: Dark purple powder, 60% yield, 370 °C (decomposition) mp. ^1^H NMR (DMSO-*d*_6_, 500 MHz) δ/ppm; 8.55 (s, 4H), 7.59 (s, 4H), 7.26 (s, 4H), 1.50 (s, 18H), 1.33 (s, 18H); ^13^C NMR (DMSO-*d*_6_, 125 MHz) δ/ppm; 163.2, 162.7, 161.9, 161.8, 158.0, 157.5, 157.4, 156.3, 155.1, 152.6, 125.0, 124.6, 124.2, 124.1, 122.4, 122.1, 122.0, 121.5, 121.1, 120.1, 36.1, 35.8, 35.7, 30.7, 30.6, 30.5, 30.4. HRMS; calcd for C_36_H_48_Cl_2_N_4_Ru [M + H]^+^; 709.2378, found; 709.2895.

##### 4.2.1.3 Synthesis of RuL_2_S Complexes

Ru–ligand complex (RuL_2_Cl_2_) (1 mmol) and sorafenib (Srf) (1 mmol) were stirred in methanol overnight at 60 °C. At the end of the reaction, methanol was removed. The solid was dried and purified by flash chromatography.^32,33^

Ru(bpy)_2_Srf (Ru1S): Red powder, 30% yield, 196 °C mp. ^1^H NMR (CD_3_OD, 500 MHz) δ/ppm; 8.73 (d, *J =* 8.1 Hz, 1H), 8.70 (d, *J =* 7.6 Hz, 2H), 8.63 (d, *J =* 8.0 Hz, 1H), 8.60 (d, *J =* 7.9 Hz, 1H), 8.23–8.17 (m, 2H), 8.15–8.13 (m, 1H), 8.07 (d, *J =* 2.6 Hz, 1H), 8.03–7.99 (m, 2H), 7.95 (td, *J =* 8.0, 1.4 Hz, 1H), 7.81 (d, *J =* 5.7 Hz, 1H), 7.78–7.74 (m, 1H), 7.71 (d, *J =* 5.6, 1H), 7.68–7.65 (m, 1H), 7.70–7.50 (m, 4H), 7.48 (d, *J =* 8.7 Hz, 1H), 7.37 (td, *J =* 7.3, 1.2 Hz, 1H), 7.32 (td, *J =* 7.3, 1.3 Hz, 1H), 7.16–7.12 (m, 2H), 7.11 (dd, *J =* 6.4, 2.6 Hz, 1H), 2.95 (s, 3H); ^13^C NMR (CD_3_OD, 125 MHz) δ/ppm; 172.0, 166.8, 159.1, 158.2, 157.9, 157.4, 153.4, 153.1, 152.8, 151.9, 151.6, 151.4, 150.5, 148.0, 138.7, 137.5, 137.3, 137.0, 136.2, 131.6, 127.5, 127.4, 126.8, 126.5, 124.1, 123.8, 123.7, 123.5, 122.8, 121.2, 121.0, 117.3, 117.2, 114.1, 26.5. HRMS; calcd for C_41_H_32_ClF_3_N_8_O_3_Ru [M – H]^−^; 877.1203, found; 877.1192.

Ru(4,4-dimebpy)_2_Srf (Ru2S): Red powder, 52% yield, 215 °C mp. ^1^H NMR (DMSO-*d*_6_, 500 MHz) δ/ppm; 10.92 (s, 1H), 10.23 (s, 1H), 9.89 (s, 1H), 8.72 (d, *J =* 13.7 Hz, 2H), 8.64 (d, *J =* 15.9 Hz, 2H), 8.50 (s, 1H), 8.44 (d, *J =* 5.7 Hz, 1H), 8.11 (d, *J =* 1.6 Hz, 1H), 7.88 (d, *J =* 5.7 Hz, 1H), 7.64–7.61 (m, 2H), 7.61–7.57 (m, 3H), 7.52–7.50 (m, 2H), 7.48 (d, *J =* 6.4 Hz, 1H), 7.45 (d, *J =* 5.8 Hz, 1H), 7.25 (dd, *J =* 5.8, 1.0 Hz, 1H), 7.20–7.19 (m, 2H), 7.18–7.17 (m, 1H), 7.09 (dd, *J =* 6.4, 2.6 Hz, 1H), 2.84 (d, *J =* 4.6 Hz, 3H), 2.47 (s, 3H), 2.44 (s, 3H); ^13^C NMR (DMSO-*d*_6_, 125 MHz) δ/ppm; 171.3, 170.7, 164.8, 157.4, 156.6, 156.2, 155.8, 152.2, 152.0, 151.4, 150.6, 150.3, 150.2, 149.2, 148.4, 148.3, 148.1, 147.1, 146.4, 138.8, 137.1, 131.4, 127.8, 127.7, 127.3, 127.0, 124.4, 124.0, 123.8, 123.2, 121.9, 121.4, 121.1, 120.6, 119.2, 116.6, 115.5, 115.5, 114.1, 26.7, 20.2, 20.1, 19.9, 19.8. HRMS; calcd for C_45_H_40_ClF_3_N_8_O_3_Ru [M – H]^−^; 933.1829, found; 933.1818.

Ru(6,6-dimebpy)_2_Srf (Ru3S): Red powder, 25% yield, 220 °C mp. ^1^H NMR (CD_3_OD, 500 MHz) δ/ppm; 8.60–8.54 (m, 3H), 8.51 (d, *J =* 8.0 Hz, 1H), 8.07–7.99 (m, 4H), 7.90 (t, *J =* 7.8 Hz, 1H), 7.72 (d, *J =* 2.3 Hz, 1H), 7.68 (d, *J =* 6.5 Hz, 1H), 7.62 (dd, *J =* 8.7, 2.3 Hz, 1H), 7.57 (d, *J =* 8.8 Hz, 2H), 7.5–7.39 (m, 5H), 7.08 (dd, *J =* 6.5, 2.6 Hz, 1H), 7.04 (d, *J =* 8.8 Hz, 2H), 2.69 (s, 3H), 2.36 (s, 3H), 2.06 (s, 3H), 1.99 (s, 3H), 1.73 (s, 3H); ^13^C NMR (CD_3_OD, 125 MHz) δ/ppm; 170.7, 167.5, 167.0, 166.2, 165.0, 164.7, 160.6, 159.5, 159.2, 158.3, 153.3, 151.6, 147.7, 138.7, 137.7, 137.6, 137.6, 137.0, 136.9, 131.6, 126.4, 126.2, 126.1, 126.0, 124.0, 123.9, 122.8, 122.5, 122.0, 121.3, 121.2, 121.1, 120.9, 117.2, 117.2, 116.4, 113.4, 26.1, 24.5, 24.3, 23.1, 22.7. HRMS; calcd for C_45_H_40_ClF_3_N_8_O_3_Ru [M – H]^−^; 933.1829, found; 933.1814.

Ru(4,4-di-*t*-bu-bpy)_2_Srf (Ru4S): Red powder, 65% yield, 207 °C mp. ^1^H NMR (CD_3_OD, 500 MHz) δ/ppm; 8.73 (dd, *J =* 6.9, 1.7 Hz, 2H), 8.63 (dd, *J =* 9.9, 1.8 Hz, 2H), 8.57 (d, *J =* 5.9 Hz, 1H), 8.06 (d, *J =* 2.6 Hz, 1H), 8.05 (d, *J =* 2.5 Hz, 1H), 8.01 (d, *J =* 5.9 Hz, 1H), 7.80 (dd, *J =* 5.9, 1.6 Hz, 1H), 7.69 (dd, *J =* 5.9, 1.7 Hz, 1H), 7.66 (d, *J =* 6.1 Hz, 1H), 7.64–7.54 (m, 5H), 7.46 (d, *J =* 8.7 Hz, 1H), 7.41 (dd, *J =* 6.1, 1.9 Hz, 1H), 7.36 (dd, *J =* 6.1, 2.0 Hz, 1H), 7.14–7.10 (m, 3H), 2.96 (s, 3H), 1.52 (s, 9H), 1.51 (s, 9H), 1.41 (s, 9H), 1.39 (s, 9H); ^13^C NMR (CD_3_OD, 125 MHz) δ/ppm; 172.0, 166.5, 162.5, 162.3, 162.0, 161.2, 158.8, 158.0, 157.8, 157.2, 153.4, 152.7, 152.4, 151.6, 151.2, 150.9, 149.9, 148.0, 138.8, 137.5, 131.5, 128.0, 127.7, 124.6, 124.6, 124.2, 124.0, 123.9, 123.8, 122.8, 121.3, 121.1, 121.0, 120.9, 120.8, 120.6, 117.2, 115.2, 114.0, 113.3, 35.3, 35.2, 35.0, 35.0, 33.5, 31.6, 30.7, 29.3, 29.3, 29.2, 29.0, 28.8, 28.7, 26.5, 22.3. HRMS; calcd for C_57_H_64_ClF_3_N_8_O_3_Ru [M – H]^−^; 1101.3707, found; 1101.3700.

Ru(4,4-dimethoxybpy)_2_Srf (Ru5S): Red powder, 80% yield, 235 °C (decomposition) mp. ^1^H NMR (CD_3_OD, 500 MHz) δ/ppm; 8.39 (d, *J =* 6.4 Hz, 1H), 8.28 (d, *J =* 2.6 Hz, 1H), 8.25 (d, *J =* 2.6 Hz, 1H), 8.19 (dd, *J =* 5.2, 2.7 Hz, 2H), 8.03 (d, *J =* 2.5 Hz, 1H), 8.01 (d, *J =* 2.6 Hz, 1H), 7.85 (d, *J =* 6.4 Hz, 1H), 7.65 (d, *J =* 6.4 Hz, 1H), 7.61–7.52 (m, 5H), 7.48 (d, *J =* 8.7 Hz, 1H), 7.32 (dd, *J =* 6.4, 2.6 Hz, 1H), 7.22 (dd, *J =* 6.4, 2.6 Hz, 1H), 7.16–7.12 (m, 2H), 7.10 (dd, *J =* 6.4, 2.6 Hz, 1H), 6.98 (dd, *J =* 6.5, 2.7 Hz, 1H), 6.94 (dd, *J =* 6.6, 2.7 Hz, 1H), 4.09 (s, 3H), 4.08 (s, 3H), 3.99 (s, 3H), 3.98 (s, 3H), 2.95 (s, 3H); ^13^C NMR (CD_3_OD, 125 MHz) δ/ppm; 172.3, 167.2, 167.1, 166.9, 166.4, 166.1, 160.0, 159.3, 158.7, 153.4, 153.1, 152.3, 152.1, 151.9, 151.2, 148.1, 138.7, 137.5, 131.6, 122.8, 121.2, 120.9, 117.3, 117.2, 117.0, 113.9, 113.6, 113.5, 113.4, 112.7, 110.8, 110.4, 110.4, 110.2, 55.9, 55.8, 26.4. HRMS; calcd for C_45_H_40_ClF_3_N_8_O_7_Ru [M – H]^−^; 997.1626, found; 997.1596.

Ru(phen)_2_Srf (Ru6S): Red powder, 37% yield, 205 °C mp. ^1^H NMR (CD_3_OD, 500 MHz) δ/ppm; 9.18 (dd, *J =* 5.1, 1.1 Hz, 1H), 8.81 (dd, *J =* 8.3, 1.1 Hz, 2H), 8.61 (dd, *J =* 5.1, 1.1 Hz, 1H), 8.53 (dd, *J =* 8.2, 1.1 Hz, 1H), 8.48 (dd, *J =* 8.2, 1.0 Hz, 1H), 8.33 (dd, *J =* 8.9, 4.9 Hz, 2H), 8.24 (t, *J =* 8.7 Hz, 2H), 8.15 (dd, *J =* 8.2, 5.1 Hz, 1H), 8.10 (d, *J =* 2.6 Hz, 1H), 8.05 (dd, *J =* 8.2, 5.1 Hz, 1H), 8.03–7.98 (m, 2H), 7.91 (dd, *J =* 5.3, 1.1 Hz, 1H), 7.65–7.51 (m, 6H), 7.48 (d, *J =* 8.7 Hz, 1H), 7.15–7.09 (m, 2H), 7.01 (dd, *J =* 6.4, 2.6 Hz, 1H), 2.94 (s, 3H); ^13^C NMR (CD_3_OD, 125 MHz) δ/ppm; 172.3, 166.8, 154.1, 153.4, 153.2, 152.8, 151.8, 149.5, 148.7, 148.6, 148.0, 138.7, 137.5, 136.6, 136.4, 136.0, 135.2, 131.5, 131.0, 130.8, 130.8, 130.6, 127.8, 127.8, 127.7, 126.0, 125.4, 125.1, 122.8, 121.2, 120.9, 117.3, 117.2, 117.1, 114.1, 26.5. HRMS; calcd for C_45_H_32_ClF_3_N_8_O_3_Ru [M – H]^−^; 925.1203, found; 925.1192.

Ru(5-Clphen)_2_Srf (Ru7S): Red powder, 51% yield, 208 °C mp. ^1^H NMR (CD_3_OD, 500 MHz) δ/ppm; 9.25 (td, *J =* 3.5, 1.2 Hz, 1H), 9.18 (td, *J =* 3.5, 1.3 Hz, 1H), 9.07–9.01 (m, 2H), 8.79–8.73 (m, 3H), 8.7 (d, *J =* 8.7 Hz, 1H), 8.68 (t, *J =* 4.1 Hz, 1H), 8.60 (t, *J =* 5.1 Hz, 1H), 8.53 (d, *J =* 1.4 Hz, 2H), 8.49 (d, *J =* 8.2 Hz, 1H), 8.44–8.42 (m, 3H), 8.29–8.24 (m, 1H), 8.19–8.11 (m, 5H), 8.06–8.03 (m, 3H), 7.96–7.95 (m, 3H), 7.69–7.62 (m, 2H), 7.61–7.52 (m, 10H), 7.41 (d, *J =* 8.7 Hz, 2H), 7.09 (dd, *J =* 8.8, 1.3 Hz, 4H), 7.02–6.99 (m, 2H), 2.94 (d, *J =* 3.3 Hz, 6H); ^13^C NMR (CD_3_OD, 125 MHz) δ/ppm; 173.8, 172.3, 167.8, 166.9, 155.1, 154.6, 153.9, 153.7, 153.4, 153.3, 153.3, 153.2, 152.1, 151.7, 149.1, 148.6, 147.9, 147.2, 138.7, 137.6, 136.0, 135.8, 135.4, 133.7, 133.5, 132.3, 132.1, 131.7, 131.5, 131.5, 130.9, 130.2, 130.0, 129.3, 129.1, 128.4, 127.0, 126.9, 126.9, 126.6, 126.5, 126.0, 125.7, 124.0, 122.8, 122.7, 121.1, 120.9, 117.2, 117.1, 114.2, 113.3, 29.3, 28.7. HRMS; calcd for C_45_H_30_Cl_3_F_3_N_8_O_3_Ru [M + H]^+^; 995.0580, found; 995.0386.

Ru(5-mephen)_2_Srf (Ru8S): Red powder, 18% yield, 195 °C mp. ^1^H NMR (CD_3_OD, 500 MHz) δ/ppm; 9.19 (t, *J =* 5.0 Hz,1H), 9.12 (t, *J =* 5.0 Hz, 1H), 8.95–8.91 (m, 2H), 8.73–8.69 (m, 2H), 8.66–8.61 (m, 2H), 8.58 (d, *J =* 8.3 Hz, 1H), 8.54 (t, *J =* 5.2 Hz, 1H), 8.42 (d, *J =* 8.1 Hz, 1H), 8.37 (d, *J =* 8.1 Hz, 1H), 8.23–8.18 (m, 1H), 8.18–8.14 (m, 4H), 8.06 (d, *J =* 4.1 Hz, 2H), 8.02–7.99 (m, 4H), 7.93 (d, *J =* 5.0 Hz, 2H), 7.86–7.83 (m, 1H), 7.64–7.46 (m, 14H), 7.43 (d, *J =* 8.7 Hz, 2H), 7.12 (td, *J =* 8.9, 1.6 Hz, 4H), 7.06–7.00 (m, 2H), 2.99 (s, 3H), 2.97 (s, 6H), 2.96 (s, 3H), 2.90 (d, *J =* 5.0 Hz, 6H); ^13^C NMR (CD_3_OD, 125 MHz) δ/ppm; 173.7, 172.2, 167.8, 166.7, 153.6, 153.3, 153.2, 153.1, 152.3, 151.8, 151.7, 151.3, 150.8, 149.8, 148.8, 148.0, 147.9, 147.9, 138.7, 138.7, 137.5, 136.4, 136.2, 136.1, 135.7, 135.5, 135.1, 134.3, 133.6, 133.4, 133.0, 132.3, 132.1, 131.5, 131.2, 130.9, 130.8, 130.6, 130.4, 129.1, 128.4, 128.0, 127.7, 126.6, 126.5, 126.5, 126.0, 125.8, 125.4, 125.2, 125.1, 124.9, 124.0, 123.9, 122.9, 122.8, 122.7, 121.8, 121.1, 120.9, 117.2, 117.2, 117.1, 115.2, 114.1, 113.3, 29.3, 28.7, 24.1, 23.5, 22.6, 22.3. HRMS; calcd for C_47_H_36_ClF_3_N_8_O_3_Ru [M]^−^; 953.1516, found; 953.1502.

### 4.3 Biological Activity

#### 4.3.1 Stability Studies

Absorption spectra of freshly prepared solutions of the samples were acquired at 25 °C in the range of 200–800 nm with a Shimadzu UV-1280 spectrophotometer, taking into account the solvent cutoff. Samples were dissolved in the proper solvents, and the resulting solutions were placed in QS quartz cuvettes (path length 1 cm).^40^

#### 4.3.2 EGFR Inhibition Assay

Enzyme activity was performed using the TAKARA Universal Tyrosine Kinase Assay and Wash and Stop Solution for Sulfuric Acid Free ELISA according to Ölgen et al.^36^ The enzyme activity reaction was performed on a precoated 96-well plate. To determine the activity, 40 μL of hEGFR and 10 μL of ATP-2Na solution were added to each well on the plate and incubated at 37 °C for 30 min. Then, the solution was removed from the well and the wells were washed 4 times with wash buffer. After this process, 100 μL of blocking solution was added and incubated for another 30 min at 37 °C. The blocking solution was discarded and the wells were washed again with wash buffer. Subsequently, 50 μL of antiphosphotyrosine (PY20-HRP) solution was added to each well and incubated at 37 °C for 30 min. Afterward, the antibody solution was removed and the wells were washed 4 times with the washing solution. 100 μL of HRP substrate solution (TMBZ) was added to the wells and the plate was incubated at 37 °C for 15 min. In addition to this mixture, 100 μL of stop solution was added to the wells in the same order that the substrate solution was added. Activity was measured at 450 nm.

In the studies to determine the inhibition potentials of the synthesized molecules on hEGFR, the solutions of different concentrations were prepared by dissolving the molecules with ethanol. The volumes were adjusted so that the solvent amount in the reaction medium was 3% and preincubated with the enzyme (Copeland 2005) (ISBN: 978-1-118-48813-3). The reaction was repeated only in the presence of this solvent to determine the effect of the solvent on the enzyme. Using the results obtained as a result of the inhibition studies, % relative activity–inhibitor concentration graph was drawn for each molecule and the inhibitory concentration at which the activity decreased by 50% was determined as the IC_50_ value.

#### 4.3.3 Cytotoxicity Assay

HepG2 (hepatocellular carcinoma; ATCC HB-8065), Caco-2 (Colon carcinoma; ATCC HTB-37), HT-29 (colon carcinoma; ATCC HTB-37), MCF-7 (Breast cancer; ATCC HTB-22), A549 (lung carcinoma; ATCC CCL-185), and healthy HEK293T (embryonic kidney epithelial; ATCC CRL-3216) cell lines were cultured in DMEM-F12 medium containing 10% fetal bovine serum (FBS) and antibiotic (100 U/mL penicillin and 100 μg/mL streptomycin). The cells were incubated at 37 °C under an atmosphere of 5% CO_2_ and 95% humidity. HepG2-SR cells were selected according to the short-term sorafenib exposure protocol.^41^ The cells were exposed to sorafenib at concentrations of 1, 2, and 4 μM for 72 h in gradual increments. At each stage, surviving cells were plated in sorafenib-free medium. They were exposed to the next higher concentration of sorafenib by passaging 3 times at each concentration. Resistant cells were maintained in medium containing 4 μM sorafenib.

Effects of synthesized compounds on cell viability of HepG2, Caco-2, HT-29, MCF-7, A549, and HEK293T cell lines tested with MTT (3-(4,5-dimethylthiazol-2-yl)-2,5-diphenyltetrazolium bromide) assay. The cells were passaged as 5 × 10^3^ per well in basal medium and incubated for adherence in 96-well plates. Sorafenib and synthesized sorafenib–ruthenium complexes were added at 10 decreasing concentrations between 100 and 0.2 μM and incubated for 48 h. At the end of 48 h, 10 μL of yellow-colored MTT agent was added and plates were incubated at 37 °C for 4 h. Formazan crystals formed after 4 h were dissolved with 100 μL of DMSO, and absorbance was measured at 540 nm. Viable cells produced darker color and nonviable cells produced lighter color because they could not convert MTT to formazan. The % viability was calculated relative to the negative controls. Concentration–viability graphs were drawn for each test substance in the GraphPad Prism 9.00, and the IC_50_ values of the substances were calculated for each cell line.^42^

#### 4.3.4 Apoptosis Assay

The apoptotic properties of the synthesized compounds were determined in HepG2 cell line using Muse Annexin V & Dead Cell reagent in Muse Cell Analyzer. HepG2 cells were seeded in 24-well plates and synthesized substances were added at 3 or 4 different concentrations (0.78, 3.125, 12.5, and 50 μM). After 48 h of incubation, cells were harvested with trypsin and transferred to PBS. 100 μL of Muse Annexin V & Dead Cell reagent was added to the cell suspension in 100 μL of PBS. The cells were allowed to stain for 30 min at room temperature in the dark. The apoptotic profiles of the substances were determined by flowing the samples in the Muse Cell Analyzer. Results are given as a percentage (%).^43^

There are two different dyes in the reagent: Annexin V (apoptosis marker) and 7-AAD (dead cell marker). The profiles of the cells after staining with these dyes according to their viability are as follows:

Bottom-left: Living cells [Annexin V (−) and 7-AAD (−)]

Bottom-right: Early apoptotic cells [Annexin V (+) and 7-AAD (−)]

Top-right: Late apoptotic or dead cells by apoptosis [Annexin V (+) and 7-AAD (+)]

Top-left: Necrotic cells [Annexin V (−) and 7-AAD (+)]

#### 4.3.5 Cell Cycle Assay

The effects of the synthesized compounds on the cell cycle in the HepG2 cell line were determined using the Muse Cell Cycle reagent in Muse Cell Analyzer. HepG2 cells were seeded in 24-well plates and synthesized substances were added at 3 or 4 different concentrations (0.78, 3.125, 12.5, and 50 μM). After 48 h of incubation, cells were harvested with trypsin and washed with PBS. The cell pellet was fixed in cold 70% ethanol for 3 h at 20 °C. The fixed cells were washed again with PBS, the supernatant was discarded, and 200 μL of Muse Cell Cycle reagent was added. The cells were allowed to stain for 30 min at room temperature in the dark. The effects of the substances on the cell cycle were determined by flowing the samples in the Muse Cell Analyzer device. Results are given as a percentage (%).

The assay uses PI-based staining of DNA content to distinguish and quantify the percentage of cells in each cell cycle phase (G0/G1, S, and G2/M). Cells in G0/G1 phase are selected in blue, the S phase in purple, and the G2/M phase in green.^44^

#### 4.3.6 Western Blot Assay

Effects of synthesized compounds on the expression of proteins in the tyrosine kinase pathway (Egfr, p-Egfr, Akt, p-Akt, Erk1/2 (p44/42 MAPK), p-Erk1/2 (p-p44/42 MAPK)) in HepG2 cell line was determined by the immunoblotting method. HepG2 cells were seeded in 6-well plates and synthesized substances were added at 2 different concentrations (12.5 μM and/or 3.125 μM and/or 0.78 μM). After 48 h of incubation, cells were harvested with trypsin. The cells were washed with cold PBS and were transferred to Ripa cell lysis buffer (Santa Cruz). They were centrifuged at 14000 rpm at +4 °C for 20 min. Protein concentration was measured with Qubit 2.0 Fluorometer. Samples were boiled at 95 °C for 5 min in Laemmli sample buffer (Bio-Rad). Samples were run on 4–10% polyacrylamide gels. Samples were transferred to the PVDF membrane by a cold transfer device (Bio-Rad) and blocked with 5% skim milk or 1% bovine serum albumin (BSA). Membranes were incubated with primary antibodies overnight for EGFR (Abclonal A2909), p-EGFR (Abclonal AP0301), Akt (CST 4691), p-Akt (CST 4060), Erk1/2 (CST 4695), p-Erk1/2 (CST 9101) and 1 h at RT for GAPDH (Abclonal). After secondary antibody incubation for 1 h at RT, images were taken on Bio-Rad Chemidoc imaging device using LumiGLO (CST) chemiluminescence substrate. Bands were calculated with the Image Lab (Bio-Rad) and were analyzed with GraphPad Prism 9.00.^42^

#### 4.3.7 Antiangiogenic Activity

The antiangiogenic effects of the synthesized compounds were evaluated by the Western blot method on the expression of proteins in the angiogenesis pathway (VEGFR, Akt, p-Akt, Erk1/2 (p44/42 MAPK), p-Erk1/2 (p-p44/42 MAPK) in the HUVEC (Human umbilical vein endothelial) cell line). HUVEC cells were seeded in 6-well plates and synthesized substances were added at 2 different concentrations (12.5 μM and/or 3.125 μM and/or 0.78 μM). After 48 h of incubation, cells were induced with 100 ng/mL VEGFR for 15 min and harvested with trypsin. The same procedures were applied as 4.3.5. VEGFR (CST 9698) antibody incubation was made overnight.^42^

### 4.4 Computational Studies

Molecular Docking studies were carried out by Schrödinger Molecular Modeling Software (2023-1) with Maestro (13.5) interface. MD simulations were carried out using Desmond (D. E. Shaw Research) with Maestro (13.1) interface.

#### 4.4.1 Preparation of Ligands and Proteins

Two-dimensional structures of the ligands were drawn with Chem-Draw. Initially, the coordination bonds of the ruthenium complex were drawn as normal bonds and the molecule was transferred to Schrödinger. By selecting the molecule on Schrödinger’s workspace, the covalent bonds between the metal and the ligand were set as coordination interactions using the “create zero-order bonds to metal” parameter under the build tab. Then, the energy minimization of the molecule was achieved by using the minimize parameter under the edit tab of Schrödinger Maestro and the preparation of the ligands was completed. The verification of correct bond orders has been accomplished by assigning bond orders through the Edit → Assign → Bond Orders menu. Additionally, to ensure a reasonable coordination geometry and prevent distorted structures, the “Perform postdocking minimization” setting in the Output tab of the Glide ligand docking application has been unchecked.^45^ The 3D X-ray crystallographic structures of the target proteins were obtained from the Protein Data Bank, accessible via the RCSB Web site (https://www.rcsb.org). In this study, epidermal growth factor receptor (EGFR) protein (PDB ID: 5X2A, the crystal resolution: 1.85 Å, origin: *Homo sapiens*) was used to carry out all *in silico* studies. Careful preparation and refinement of these protein structure was carried out using the Protein Preparation Wizard module within Schrödinger Maestro 13.5.^46^

#### 4.4.2 Glide Docking and Induced Fit Docking

The methods previously published by our research group were used to perform glide docking and induced fit docking.^47,48^ The ligands that were prepared underwent docking with the receptors using the Induced Fit Docking (IFD) protocol in Schrödinger Release 2023-1. The IFD protocol, integrated into the Schrödinger suite, employs Glide (Schrödinger, LLC, New York, NY). The centroid of the native ligand served as the center for the Glide grid, with the inner box side set to 20 Å and the outer box side automatically determined. The binding energy, represented by the IFD Score, was computed for each generated pose, and the resulting poses for each protein–ligand complex underwent thorough visual examination.^49^

#### 4.4.3 Prime MM-GBSA Analysis

The methods previously published by our research group were used to perform prime MM-GBSA analysis.^50^ The calculation of the free binding energy for the docked poses were carried out utilizing the Prime MM/GBSA (Molecular Mechanics Generalized Born Surface Area) module within the Schrödinger suite 2023. Default parameters were applied in the MM/GBSA module, including the VSGB solvation model and the Minimize sampling method. To account for flexibility, constraints on flexible residues were implemented, designating the residues around the receptor pocket as having flexible conformations during the energy calculation.^51^

#### 4.4.4 Molecular Dynamics Simulations

The methods previously published by our research group were used to perform molecular dynamics simulations.^52^ The Desmond System Builder module was employed to configure the complex within a solvent-soaked orthorhombic periodic box, with a selected orthorhombic box size of 10 Å as boundary conditions. To achieve a neutral system charge, Na^+^ and Cl^–^ ions were added, resulting in a 0.15 M NaCl salt concentration. The constructed solvated system underwent minimization using the OPLS3e force field. System equilibration occurred at 300 K and 1.01325 bar in the NPT (normal pressure and temperature) ensemble. Simulations were conducted over a 200 ns time frame, with data recorded at 200 ps intervals. Molecular dynamic trajectories were generated and analyzed utilizing Desmond’s Simulation Interaction Diagram (SID).^47^

#### 4.4.5 Molecular Docking Validations

The methods previously published by our research group were used to perform molecular docking validations.^53^

### 4.5 Formation of Micelles

PEG–PLGA polymeric micelles containing **Ru3S** or **Ru4S** complexes, which showed the best results in the cytotoxicity test, were formed by the coprecipitation method. For this purpose, 1 mg of either **Ru3S** or **Ru4S** complex and poly(ethylene glycol) methyl ether-*block*-poly(lactide-*co*-glycolide) (PEG–PLGA, PEG average *M*_n_ 5000, PLGA average *M*_n_ 10,000, lactide:glycolide 50:50, 10 mg) polymer were mixed overnight in a vial containing 1 mL of DMSO. The next day, micelle (“self-assembly”) structures were formed by adding dropwise into a vial containing 4 mL of water and the solution was stirred for 24 h. Then, DMSO was removed by dialysis.^54^ The micelle formation process of the amphiphilic polymer without any complexes was followed by using a similar procedure. M refers to PEG–PLGA polymeric micelles without any complexes. **M1** and **M2** refer to **Ru3S**- and **Ru4S**-loaded polymeric micelles.

### 4.6 Release Studies

The release profile of **Ru4S** from **M2** was evaluated by the dialysis method in two different pH buffer solutions (pH = 5.5 and pH 7.4). 2 mL of **M2** was placed into a dialysis bag with a molecular weight cutoff of 3.50 kDa and dialyzed against 50 mL of phosphate-buffered saline (PBS) in an orbital shaker with a stirring speed of 100 rpm at 37 °C. 2 mL of samples was taken at a certain time point and fresh buffer solution was replaced in the same volume instead. To quantitatively determine the amount of drug released, the taken samples at each time interval were analyzed by LC-HRMS.^55−57^

## Supporting Information Available

^1^H, ^13^C NMR, and MS spectra of complexes, and viability (%) – log concentration (μM) curves for the cytotoxic effects; graphs showing the tyrosine kinase inhibition and apoptosis; and cell cycle profiles of complexes; molecular docking 2D and 3D ligand–protein interaction poses of rest of the Ru complexes (PDF)
Molecular formula strings (CSV)

## Abbreviations Used

Akt: protein kinase B
DLC: drug loading content
EE: entrapment efficiency
GAPDH: glyceraldehyde-3-phosphate dehydrogenase
IFD: induced fit docking
MD: molecular dynamics
MEK: mitogen-activated protein kinase
MM-GBSA: molecular mechanics with generalized born and surface area
MTT: 3-(4,5-dimethylthiazol-2-yl)-2,5-diphenyltetrazolium bromide
PDGFR: platelet-derived growth factor receptor
PLGA: poly(lactic-*co*-glycolic acid)
RAF: rapidly accelerated fibrosarcoma
RMSF: root-mean-square fluctuation
TKs: tyrosine kinases
XP: extra precision

## References

[ref1] Zhong H. J.; Wang W. H.; Kang T. S.; Yan H.; Yang Y. L.; Xu L. P.; Wang Y. Q.; Ma D. L.; Leung C. H. A Rhodium(III) Complex as an Inhibitor of Neural Precursor Cell Expressed, Developmentally Down-Regulated 8-Activating Enzyme with in Vivo Activity against Inflammatory Bowel Disease. J. Med. Chem. 2017, 60 (1), 497–503. 10.1021/acs.jmedchem.6b00250. 27976900

[ref2] Kurzwernhart A.; Kandioller W.; Bachler S.; Bartel C.; Martic S.; Buczkowska M.; Muhlgassner G.; Jakupec M. A.; Kraatz H. B.; Bednarski P. J.; Arion V. B.; Marko D.; Keppler B. K.; Hartinger C. G. Structure-Activity Relationships of Targeted Ru-II(eta(6)-p-Cymene) Anticancer Complexes with Flavonol-Derived Ligands. J. Med. Chem. 2012, 55 (23), 10512–10522. 10.1021/jm301376a. 23134291

[ref3] Thota S.; Rodrigues D. A.; Crans D. C.; Barreiro E. J. Ru(II) Compounds: Next-Generation Anticancer Metallotherapeutics?. J. Med. Chem. 2018, 61 (14), 5805–5821. 10.1021/acs.jmedchem.7b01689. 29446940

[ref4] Skoczynska A.; Lux K.; Mayer P.; Lorenz I.-P.; Krajewska U.; Rozalski M.; Dołęga A.; Budzisz E. Spectroscopic and cytotoxic characteristics of (p-cymene)Ru(II) complexes with bidentate coumarins and density functional theory comparison with selected Pd(II) complexes. Inorg. Chim. Acta 2017, 456, 105–112. 10.1016/j.ica.2016.10.036.

[ref5] Skoczynska A.; Małecka M.; Cieslak M.; Kazmierczak-Baranska J.; Krolewska-Golinska K.; Leniart A.; Budzisz E. Synthesis, structural analysis, redox properties and in vitro antitumor evaluation of half-sandwich complexes of Ru(II) with aminocoumarins. Polyhedron 2017, 127, 307–314. 10.1016/j.poly.2017.02.011.

[ref6] Karges J.; Tharaud M.; Gasser G. Polymeric Encapsulation of a Ru(II)-Based Photosensitizer for Folate-Targeted Photodynamic Therapy of Drug Resistant Cancers. J. Med. Chem. 2021, 64 (8), 4612–4622. 10.1021/acs.jmedchem.0c02006. 33818111

[ref7] Romero-Canelón I.; Salassa L.; Sadler P. J. The contrasting activity of iodido versus chlorido ruthenium and osmium arene azo- and imino-pyridine anticancer complexes: control of cell selectivity, cross-resistance, p53 dependence, and apoptosis pathway. J. Med. Chem. 2013, 56 (3), 1291–1300. 10.1021/jm3017442. 23368735

[ref8] Chen W. X.; Song X. D.; He S. F.; Sun J.; Chen J. X.; Wu T.; Mao Z. W. Ru(II) complexes bearing guanidinium ligands as potent anticancer agents. J. Inorg. Biochem. 2016, 164, 91–98. 10.1016/j.jinorgbio.2016.09.004. 27666421

[ref9] Pelletier F.; Comte V.; Massard A.; Wenzel M.; Toulot S.; Richard P.; Picquet M.; Le Gendre P.; Zava O.; Edafe F.; Casini A.; Dyson P. J. Development of Bimetallic Titanocene-Ruthenium-Arene Complexes As Anticancer Agents: Relationships between Structural and Biological Properties. J. Med. Chem. 2010, 53 (19), 6923–6933. 10.1021/jm1004804. 20822096

[ref10] Skoczynska A.; Malecka M.; Cieslak M.; Kazmierczak-Baranska J.; Krolewska-Golinska K.; Leniart A.; Budzisz E. Synthesis, structural analysis, redox properties and in vitro antitumor evaluation of half-sandwich complexes of Ru(II) with aminocoumarins. Polyhedron 2017, 127, 307–314. 10.1016/j.poly.2017.02.011.

[ref11] Chen J.; Tao Q.; Wu J.; Wang M. M.; Su Z.; Qian Y.; Yu T.; Wang Y.; Xue X. L.; Liu H. K. A lysosome-targeted ruthenium(II) polypyridyl complex as photodynamic anticancer agent. J. Inorg. Biochem. 2020, 210, 111132 10.1016/j.jinorgbio.2020.111132. 32569884

[ref12] Caruso F.; Rossi M.; Benson A.; Opazo C.; Freedman D.; Monti E.; Gariboldi M. B.; Shaulky J.; Marchetti F.; Pettinari R.; Pettinari C. Ruthenium-Arene Complexes of Curcumin: X-Ray and Density Functional Theory Structure, Synthesis, and Spectroscopic Characterization, in Vitro Antitumor Activity, and DNA Docking Studies of (p-Cymene)Ru(curcuminato)chloro. J. Med. Chem. 2012, 55 (3), 1072–1081. 10.1021/jm200912j. 22204522

[ref13] Solís-Ruiz J. A.; Barthe A.; Riegel G.; Saavedra-Diaz R. O.; Gaiddon C.; Le Lagadec R. Light activation of cyclometalated ruthenium complexes drives towards caspase 3 dependent apoptosis in gastric cancer cells. J. Inorg. Biochem. 2020, 208, 111080 10.1016/j.jinorgbio.2020.111080. 32330762

[ref14] Kaulage M. H.; Maji B.; Pasadi S.; Bhattacharya S.; Muniyappa K. Novel ruthenium azo-quinoline complexes with enhanced photonuclease activity in human cancer cells. Eur. J. Med. Chem. 2017, 139, 1016–1029. 10.1016/j.ejmech.2017.08.059. 28910739

[ref15] He S. F.; Chen B. B.; Hao Y. H.; Chen J. X.; Song X. D.; Mei J.; Chen W. X.; Sun J. Synthesis, characterization and anticancer activity of two Ru(II) polypyridyl complexes [Ru(dpq)(2)L](PF6)(2) (L = maip, paip). Inorg. Chim. Acta 2018, 480, 62–69. 10.1016/j.ica.2018.05.006.

[ref16] Notaro A.; Frei A.; Rubbiani R.; Jakubaszek M.; Basu U.; Koch S.; Mari C.; Dotou M.; Blacque O.; Gouyon J.; Bedioui F.; Rotthowe N.; Winter R. F.; Goud B.; Ferrari S.; Tharaud M.; Rezacova M.; Humajova J.; Tomsik P.; Gasser G. Ruthenium(II) Complex Containing a Redox-Active Semiquinonate Ligand as a Potential Chemotherapeutic Agent: From Synthesis to In Vivo Studies. J. Med. Chem. 2020, 63 (10), 5568–5584. 10.1021/acs.jmedchem.0c00431. 32319768

[ref17] Mari C.; Rubbiani R.; Gasser G. Biological evaluation of nitrile containing Ru(II) polypyridyl complexes as potential photodynamic therapy agents. Inorg. Chim. Acta 2017, 454, 21–26. 10.1016/j.ica.2015.10.010.

[ref18] Wilhelm S.; Carter C.; Lynch M.; Lowinger T.; Dumas J.; Smith R. A.; Schwartz B.; Simantov R.; Kelley S. Discovery and development of sorafenib: a multikinase inhibitor for treating cancer. Nat. Rev. Drug. Discovery 2006, 5 (10), 835–844. 10.1038/nrd2130. 17016424

[ref19] Gao J.; Jian J.; Jiang Z.; Van Schepdael A. Screening assays for tyrosine kinase inhibitors: A review. J. Pharm. Biomed. Anal. 2023, 223, 115166 10.1016/j.jpba.2022.115166. 36403346

[ref20] Heffeter P.; Atil B.; Kryeziu K.; Groza D.; Koellensperger G.; Korner W.; Jungwirth U.; Mohr T.; Keppler B. K.; Berger W. The ruthenium compound KP1339 potentiates the anticancer activity of sorafenib in vitro and in vivo. Eur. J. Cancer 2013, 49 (15), 3366–3375. 10.1016/j.ejca.2013.05.018. 23790465 PMC3807657

[ref21] Bregman H.; Meggers E. Ruthenium half-sandwich complexes as protein kinase inhibitors: An N-succinimidyl ester for rapid derivatizations of the cyclopentadienyl moiety. Org. Lett. 2006, 8 (24), 5465–5468. 10.1021/ol0620646. 17107048

[ref22] Atilla-Gokcumen G. E.; Williams D. S.; Bregman H.; Pagano N.; Meggers E. Organometallic compounds with biological activity: A very selective and highly potent cellular inhibitor for glycogen synthase kinase 3. ChemBioChem 2006, 7 (9), 1443–1450. 10.1002/cbic.200600117. 16858717

[ref23] Bregman H.; Carroll P. J.; Meggers E. Rapid access to unexplored chemical space by ligand scanning around a ruthenium center: Discovery of potent and selective protein kinase inhibitors. J. Am. Chem. Soc. 2006, 128 (3), 877–884. 10.1021/ja055523r. 16417378

[ref24] Debreczeni J. E.; Bullock A. N.; Atilla G. E.; Williams D. S.; Bregman H.; Knapp S.; Meggers E. Ruthenium half-sandwich complexes bound to protein kinase Pim-1. Angew. Chem., Int. Ed. 2006, 45 (10), 1580–1585. 10.1002/anie.200503468. 16381041

[ref25] Bregman H.; Williams D. S.; Atilla G. E.; Carroll P. J.; Meggers E. An organometallic inhibitor for glycogen synthase kinase 3. J. Am. Chem. Soc. 2004, 126 (42), 13594–13595. 10.1021/ja046049c. 15493898

[ref26] Yidan Lai N. L.; Lu N.; Luo S.; et al. Shuangling Luo, Haobing Wang, and Pingyu Zhang, A Photoactivated Sorafenib-Ruthenium(II) Prodrug for Resistant Hepatocellular Carcinoma Therapy through Ferroptosis and Purine Metabolism Disruption. J. Med. Chem. 2022, 65 (19), 13041–13051. 10.1021/acs.jmedchem.2c00880. 36134739

[ref27] Gillani T. B.; Rawling T.; Murray M. Cytochrome P450-Mediated Biotransformation of Sorafenib and Its N-Oxide Metabolite: Implications for Cell Viability and Human Toxicity. Chem. Res. Toxicol. 2015, 28 (1), 92–102. 10.1021/tx500373g. 25489883

[ref28] Bankston D.; Dumas J.; Natero R.; Riedl B.; Monahan M. K.; Sibley R. A scaleable synthesis of BAY 43–9006: A potent Raf kinase inhibitor for the treatment of cancer. Org. Process. Res. Dev. 2002, 6 (6), 777–781. 10.1021/op020205n.

[ref29] Kurt B. Z.; Gazioglu I.; Sonmez F.; Kucukislamoglu M. Synthesis, antioxidant and anticholinesterase activities of novel coumarylthiazole derivatives. Bioorg. Chem. 2015, 59, 80–90. 10.1016/j.bioorg.2015.02.002. 25706320

[ref30] Al-Rawashdeh N. A. F.; Chatterjee S.; Krause J. A.; Connick W. B. Ruthenium Bis-diimine Complexes with a Chelating Thioether Ligand: Delineating 1,10-Phenanthrolinyl and 2,2 ′-Bipyridyl Ligand Substituent Effects. Inorg. Chem. 2014, 53 (1), 294–307. 10.1021/ic4022454. 24325318

[ref31] Viala C.; Coudret C. An expeditious route to cis-Ru(bpy)(2)Cl-2 (bpy = 2,2 ′-bipyridine) using carbohydrates as reducers. Inorg. Chim. Acta 2006, 359 (3), 984–989. 10.1016/j.ica.2005.07.019.

[ref32] Nikolić S.; Mihajlovic-Lalic L. E.; Vidosavljevic M.; Arandelovic S.; Radulovic S.; Grguric-Sipka S. Mono- and binuclear Ru(II) arene complexes with (fluoro substituted) picolinic acid: Synthesis, characterization and cytotoxicity. J. Organomet. Chem. 2019, 902, 120966 10.1016/j.jorganchem.2019.120966.

[ref33] Shohayeb S. M.; Mohamed R. G.; Moustafa H.; El-Medani S. M. Synthesis, spectroscopic, DFT calculations and biological activity studies of ruthenium carbonyl complexes with 2-picolinic acid and a secondary ligand. J. Mol. Struct. 2016, 1119, 442–450. 10.1016/j.molstruc.2016.05.009.

[ref34] Altinkok C.; Acik G.; Karabulut H. R. F.; Ciftci M.; Tasdelen M. A.; Dag A. Synthesis and characterization of bile acid-based polymeric micelle as a drug carrier for doxorubicin. Polym. Adv. Technol. 2021, 32, 4860–4868. 10.1002/pat.5478.

[ref35] Sevgi E.; Dag A.; Kizilarslan-Hancer C.; Atasoy S.; Kurt B. Z.; Aksakal O. Evaluation of cytotoxic and antioxidant potential of Dittrichia viscosa (L.) Greuter used in traditional medicine. J. Ethnopharmacol. 2021, 276, 114211 10.1016/j.jep.2021.114211. 34015367

[ref36] Ölgen S.; Isgor Y. G.; Coban T. Synthesis and activity of novel 5-substituted pyrrolo[2,3-d]pyrimidine analogues as pp60(c-Src) tyrosine kinase inhibitors. Arch. Pharm. 2008, 341 (2), 113–120. 10.1002/ardp.200700141. 18214841

[ref37] Morgillo F.; Martinelli E.; Troiani T.; Orditura M.; De Vita F.; Ciardiello F. Antitumor activity of sorafenib in human cancer cell lines with acquired resistance to EGFR and VEGFR tyrosine kinase inhibitors. PLoS One 2011, 6 (12), e28841 10.1371/journal.pone.0028841. 22174910 PMC3235154

[ref38] Ezzoukhry Z.; Louandre C.; Trecherel E.; Godin C.; Chauffert B.; Dupont S.; Diouf M.; Barbare J. C.; Maziere J. C.; Galmiche A. EGFR activation is a potential determinant of primary resistance of hepatocellular carcinoma cells to sorafenib. Int. J. Cancer 2012, 131 (12), 2961–2969. 10.1002/ijc.27604. 22514082

[ref39] Hue R. J.; Vatassery R.; Mann K. R.; Gladfelter W. L. Zinc oxide nanocrystal quenching of emission from electron-rich ruthenium-bipyridine complexes. Dalton Trans. 2015, 44 (10), 4630–4639. 10.1039/C4DT03272A. 25655833

[ref40] Brustolin L.; Nardon C.; Pettenuzzo N.; Zuin Fantoni N.; Quarta S.; Chiara F.; Gambalunga A.; Trevisan A.; Marchio L.; Pontisso P.; Fregona D. Synthesis, chemical characterization and cancer cell growth-inhibitory activities of Cu(ii) and Ru(iii) aliphatic and aromatic dithiocarbamato complexes. Dalton Trans. 2018, 47 (43), 15477–15486. 10.1039/C8DT02965B. 30334060

[ref41] Zhou W.; Lou W.; Chen J.; Ding B.; Chen B.; Xie H.; Zhou L.; Zheng S.; Jiang D. AG-1024 Sensitizes Sorafenib-Resistant Hepatocellular Carcinoma Cells to Sorafenib via Enhancing G1/S Arrest. Onco. Targets. Ther. 2021, 14, 1049–1059. 10.2147/OTT.S289324. 33623392 PMC7894871

[ref42] Kurt B. Z.; Sonmez F.; Ozturk D.; Akdemir A.; Angeli A.; Supuran C. T. Synthesis of coumarin-sulfonamide derivatives and determination of their cytotoxicity, carbonic anhydrase inhibitory and molecular docking studies. Eur. J. Med. Chem. 2019, 183, 111702 10.1016/j.ejmech.2019.111702. 31542715

[ref43] Goncu B.; Sevgi E.; Hancer C. K.; Gokay G.; Ozten N. Differential anti-proliferative and apoptotic effects of lichen species on human prostate carcinoma cells. PLoS One 2020, 15 (12), e0244831 10.1371/journal.pone.0238303. 32997661 PMC7527208

[ref44] Abu Aboud O.; Wettersten H. I.; Weiss R. H. Inhibition of PPARalpha induces cell cycle arrest and apoptosis, and synergizes with glycolysis inhibition in kidney cancer cells. PLoS One 2013, 8 (8), e71115 10.1371/journal.pone.0071115. 23951092 PMC3737191

[ref45] Tota J.; Mahajan H. K.; Battu S.; Srilatha B. N.; Itteboina R.; Vijjulatha M. Synthesis, Characterisation, Docking Studies And Biological Activity Of Metal (II) Complexes Of Schiff Base Ligand Derived From 4-Chloro-2-Benzothiazolamine And Imidazole-2-Carboxaldehyde. J. Appl. Chem. 2016, 9 (6), 17–27.

[ref46] Tokalı F. S.; Taslimi P.; Sadeghi M.; Şenol H. Synthesis and evaluation of quinazolin-4(3H)-one derivatives as multitarget metabolic enzyme inhibitors: A biochemistry-oriented drug design. ChemistrySelect 2023, 8 (25), e202301158 10.1002/slct.202301158.

[ref47] Tahirli S.; Aliyeva F.; Şenol H.; Demukhamedova S.; Akverdieva G.; Aliyeva I.; Veysova S.; Sadeghian N.; Günay S.; Erden Y.; Taslimi P.; Sujayev A.; Chiragov F. Novel complex compounds of nickel with 3-(1-phenyl-2,3-dimethyl-pyrazolone-5)azopentadione-2,4: synthesis, NBO analysis, reactivity descriptors and in silico and in vitro anti-cancer and bioactivity studies. J. Biomol. Struct. Dyn. 2024, 1–25. 10.1080/07391102.2024.2309646. 38294759

[ref48] Şenol H.; Ghaffari-Moghaddam M.; Bulut Ş.; Akbaş F.; Köse A.; Topçu G. Synthesis and anticancer activity of novel derivatives of α,β-unsaturated ketones based on oleanolic acid: in vitro and in silico studies against prostate cancer cells. Chem. Biodivers. 2023, 20 (9), e202301089 10.1002/cbdv.202301089. 37596247

[ref49] Tokalı F. S.; Şenol H.; Bulut Ş.; Hacıosmanoğlu-Aldoğan E. Synthesis, characterization and molecular docking studies of highly selective new hydrazone derivatives of anthranilic acid and their ring closure analogue Quinazolin-4(3H)-ones against lung cancer cells A549. J. Mol. Struct. 2023, 1282, 135176 10.1016/j.molstruc.2023.135176.

[ref50] Şenol H.; Şahin R. B.; Mercümek B.; Kapucu H. B.; Hacıosmanoğlu E.; Dinç H.; Yüksel Mayda P. Synthesis of ursolic acid arylidene-hydrazide hybrid compounds and investigation of their cytotoxic and antimicrobial effects. Nat. Prod. Res. 2023, 37 (15), 2500–2507. 10.1080/14786419.2022.2051170. 35275500

[ref51] Şenol H.; Ghaffari-Moghaddam M.; Alim Toraman G. Ö.; Güller U. Novel chalcone derivatives of ursolic acid as acetylcholinesterase inhibitors: Synthesis, characterization, biological activity, ADME prediction, molecular docking and molecular dynamics studies. J. Mol. Struct. 2024, 1295, 136804 10.1016/j.molstruc.2023.136804.

[ref52] Demirkıran Ö.; Erol E.; Şenol H.; Kesdi İ. M.; Alim Toraman G. Ö.; Okudan E. Ş.; Topcu G. Cytotoxic meroterpenoids from brown alga Stypopodium schimperi (Kützing) Verlaque & Boudouresque with comprehensive molecular docking & dynamics and ADME studies. Process Biochem. 2024, 136, 90–108. 10.1016/j.procbio.2023.11.029.

[ref53] Şenol H.; Çakır F. 3-Amino-thiophene-2-carbohydrazide Derivatives as Anti Colon Cancer Agents: Synthesis, Characterization, In-Silico and In-Vitro Biological Activity Studies. ChemistrySelect 2023, 8 (39), e202302448 10.1002/slct.202302448.

[ref54] Chen X. F.; Chen J. Z.; Li B. W.; Yang X.; Zeng R. J.; Liu Y. J.; Li T.; Ho R. J. Y.; Shao J. W. PLGA-PEG-PLGA triblock copolymeric micelles as oral drug delivery system: In vitro drug release and in vivo pharmacokinetics assessment. J. Colloid Interface Sci. 2017, 490, 542–552. 10.1016/j.jcis.2016.11.089. 27923139

[ref55] Babos G.; Biro E.; Meiczinger M.; Feczko T. Dual Drug Delivery of Sorafenib and Doxorubicin from PLGA and PEG-PLGA Polymeric Nanoparticles. Polymers 2018, 10 (8), 895 10.3390/polym10080895. 30960820 PMC6403728

[ref56] Omurtag Ozgen P. S.; Atasoy S.; Kurt B. Z.; Durmus Z.; Yigit G.; Dag A. Glycopolymer decorated multiwalled carbon nanotubes for dual targeted breast cancer therapy. J. Mater. Chem. B 2020, 8 (15), 3123–3137. 10.1039/C9TB02711D. 32211704

[ref57] Dag A.; Cakilkaya E.; Ozgen P. S. O.; Atasoy S.; Erdem G. Y.; Cetin B.; Kokuroglu A. C.; Gurek A. G. Phthalocyanine-Conjugated Glyconanoparticles for Chemo-photodynamic Combination Therapy. Biomacromolecules 2021, 22 (4), 1555–1567. 10.1021/acs.biomac.0c01811. 33793222

