# Supplementary material for: Synthesis of Sorafenib−Ruthenium Complexes, Investigation of Biological Activities and Applications in Drug Delivery Systems as an Anticancer Agent
Source: J Med Chem. 2024 Mar 12;67(6):4463–82. doi: 10.1021/acs.jmedchem.3c01115 (PMC10983010; doi:10.1021/acs.jmedchem.3c01115)
Supplement: Supplementary file 1 — jm3c01115_si_001.pdf [file jm3c01115_si_001.pdf]

Supporting Information of Paper

Synthesis of Sorafenib-Ruthenium Complexes, Investigation of Biological Activities and Applications in Drug Delivery Systems as an Anticancer Agent

Belma Zengin Kurt[1*], Dilek Öztürk Civelek[2*], Elmas Begüm Çakmak[3], Yakup Kolcuoğlu[4], Halil Şenol[1], Begüm Nurpelin Sağlık Özkan[5], Aydan Dag[1], Kadriye Benkli[6]

[1]*Bezmialem Vakif University, Faculty of Pharmacy, Department of Pharmaceutical Chemistry, 34093, Istanbul, Türkiye*

[2]*Bezmialem Vakif University, Faculty of Pharmacy, Department of Pharmacology, 34093, Istanbul, Türkiye*

[3]*Sakarya University, Institute of Science, 34000, Sakarya, Türkiye*

[4]*Karadeniz Technical University, Faculty of Science, Department of Chemistry, 61080, Trabzon, Türkiye*

[5]*Anadolu University, Faculty of Pharmacy, Department of Pharmaceutical Chemistry, 26470, Eskişehir, Türkiye*

[6]*Badakbas Pharmacy, Altintepe str. Koknarli 6/C, Maltepe, 34840, Istanbul, Türkiye*

*Corresponding author: Belma Zengin Kurt; e-mail: bzengin@bezmialem.edu.tr, Dilek Öztürk Civelek; email: dozturk@bezmialem.edu.tr

Table of Contents

^{1}H NMR, ^{13}C NMR, and MS spectra of complexes S3

HPLC trace S23

Stability studies S27

IC_{50} curves of EGFR inhibition S28

IC_{50} curves of cytotoxicity S29

Apoptosis profiles S35

Cell cycle profiles S39

Repetitions of western blot experiment in HepG2 cell line S42

Repetitions of western blot experiment in HUVEC cell line S42

Entrapment efficiency (EE) equations S43

Drug Release S43

Molecular Docking 2D and 3D ligand-protein interactions S45

Molecular Docking Validations S52

Elemental Analyses S52

References S53

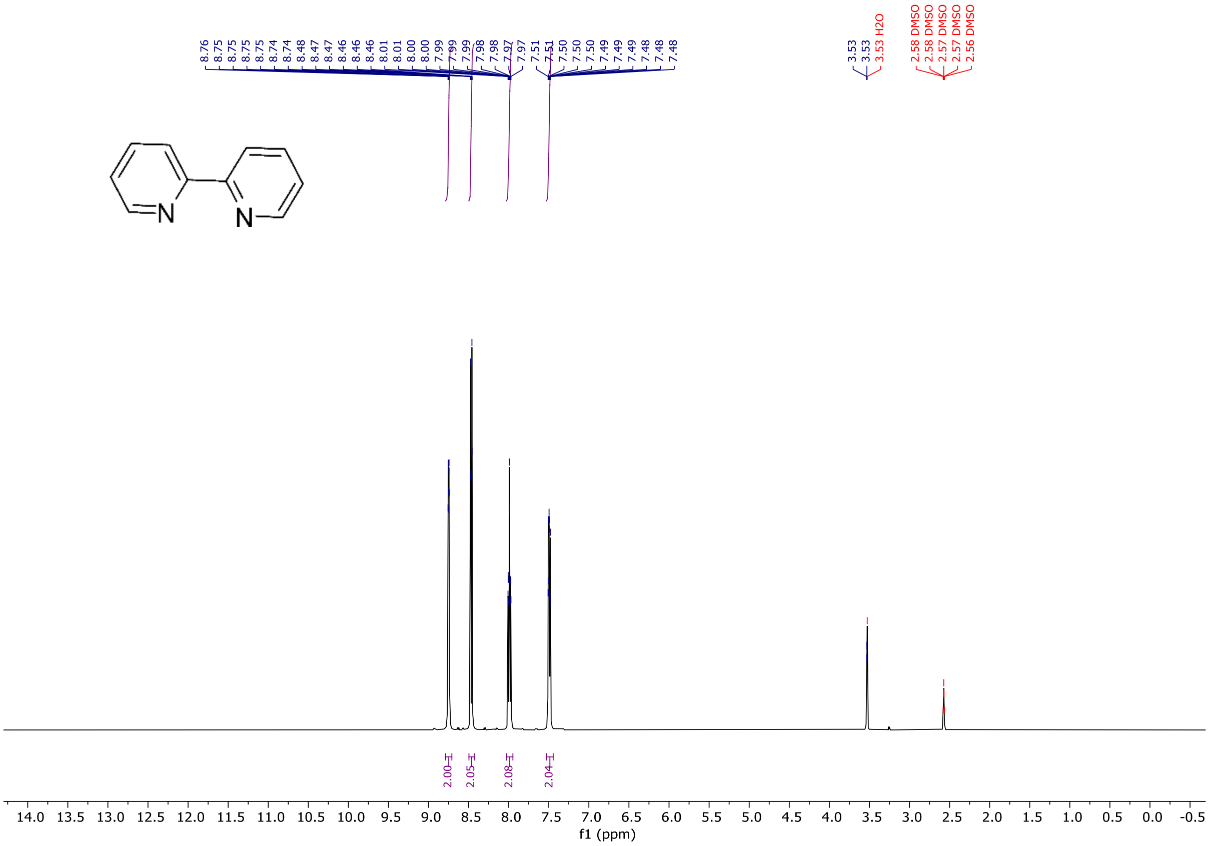

Figure S1. ^{1}H NMR (500 MHz, DMSO-*d6*) spectrum of **2,2'-Bipyridin (bpy)**

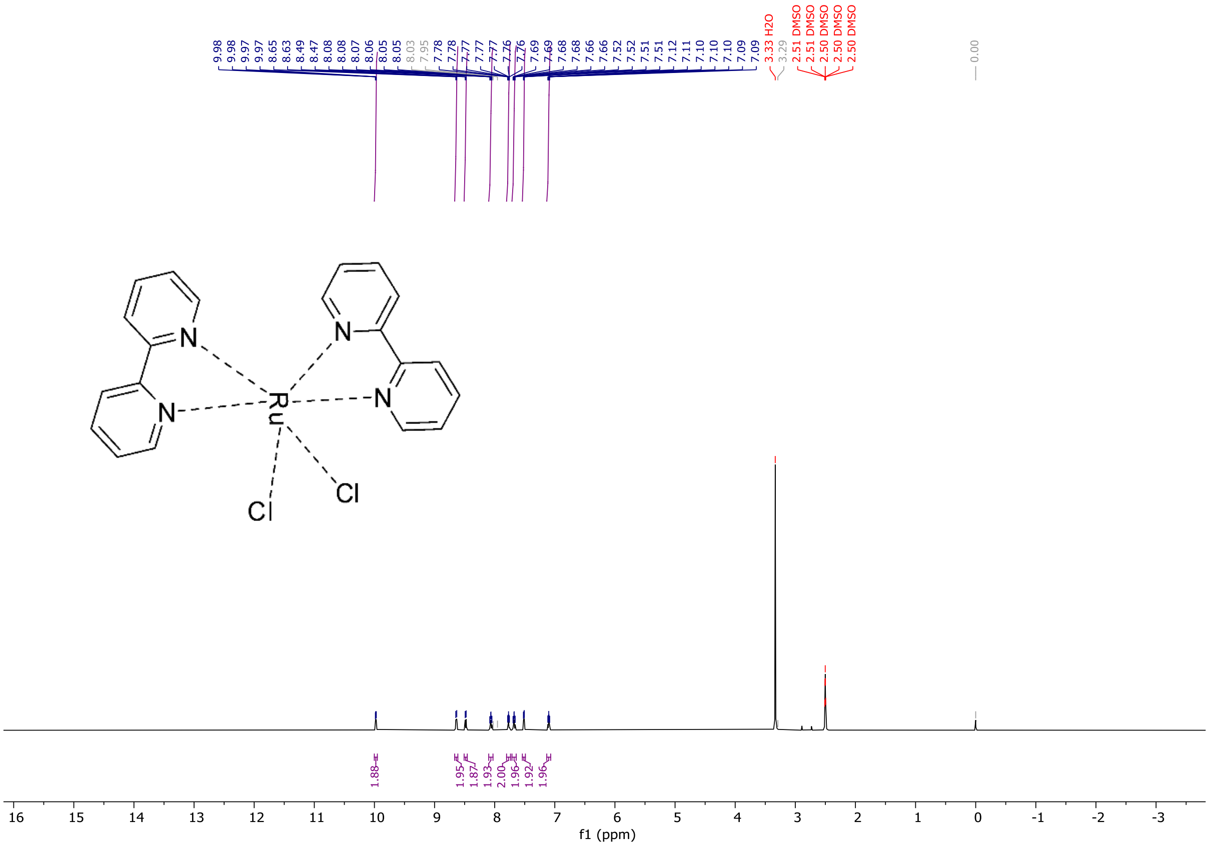

Figure S2. ^{1}H NMR (500 MHz, DMSO-*d6*) spectrum of **Ru(bpy)$_2$Cl$_2$**

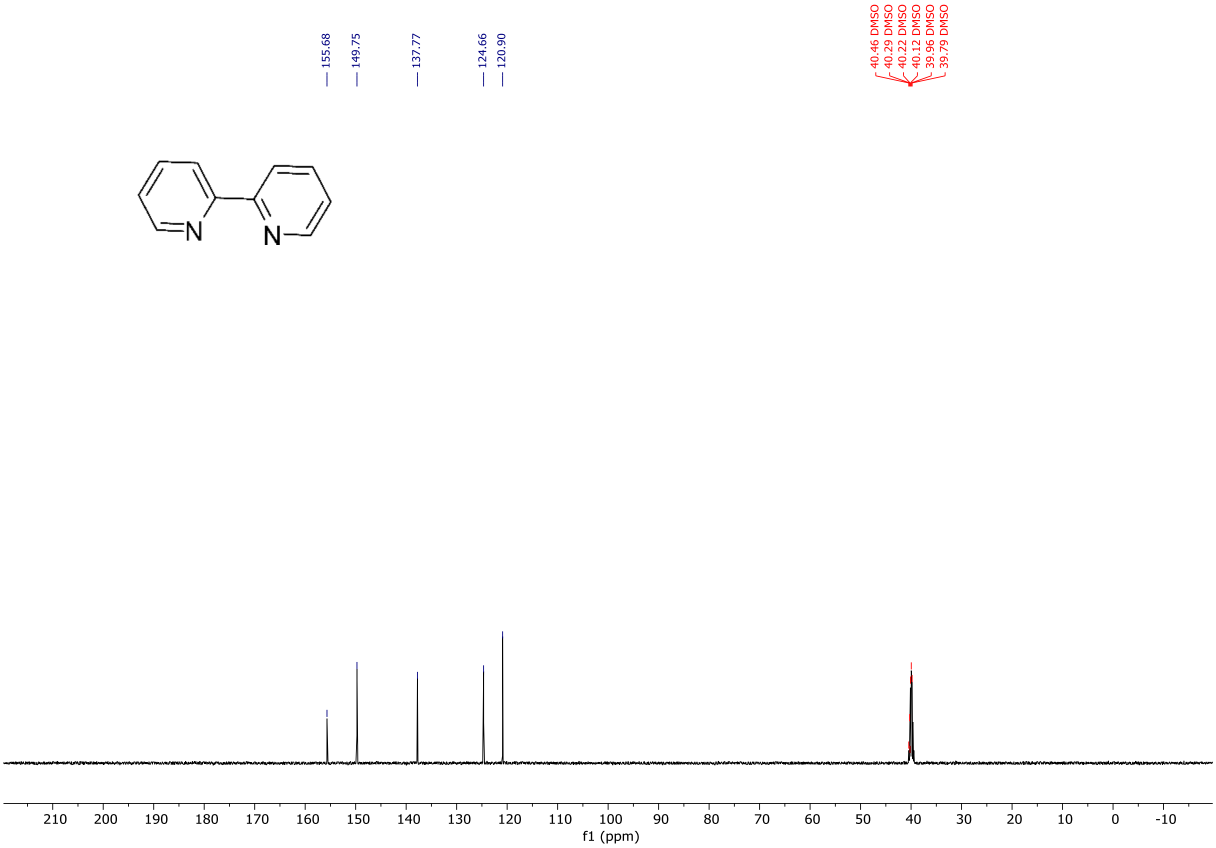

Figure S3. ^{13}C NMR (125 MHz, DMSO-*d6*) spectrum of **2,2'-Bipyridin (bpy)**

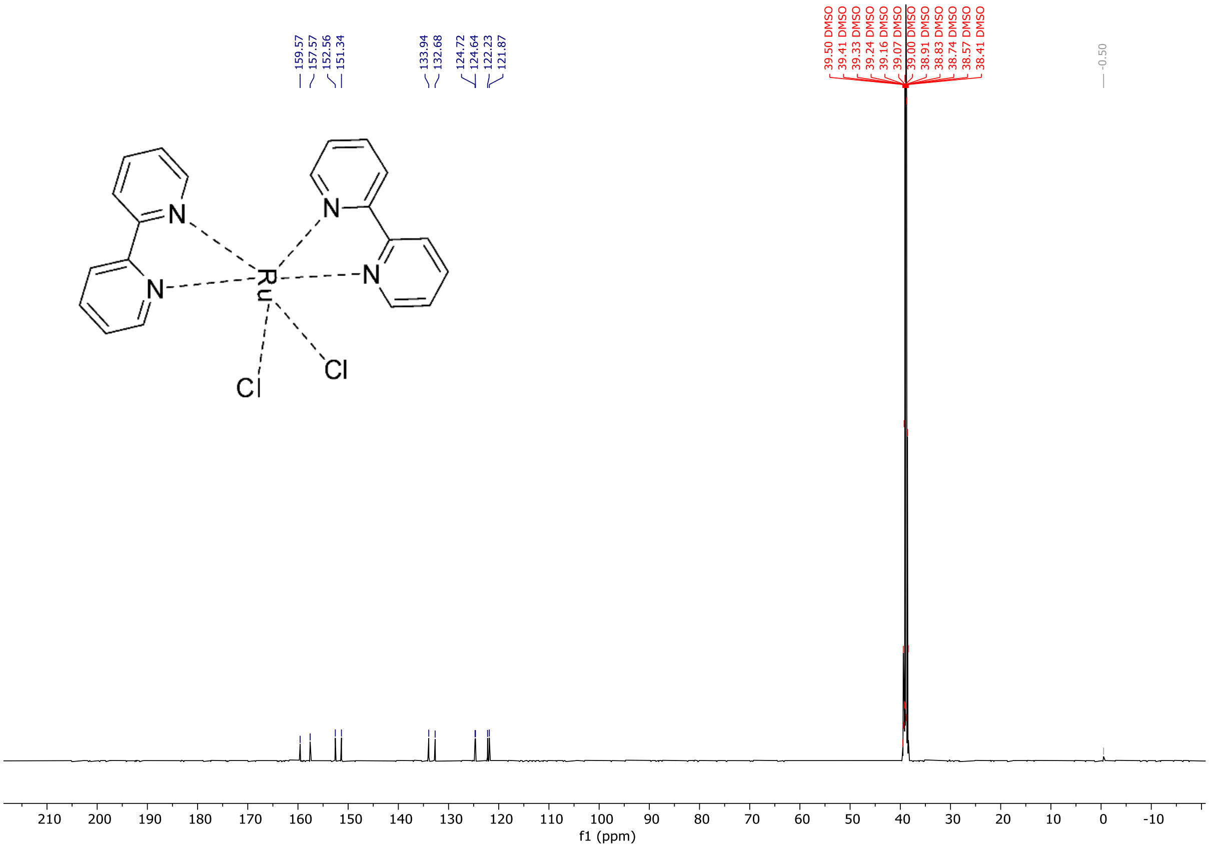

Figure S4. ^{13}C NMR (125 MHz, DMSO-*d6*) spectrum of **Ru(bpy)$_2$Cl$_2$**

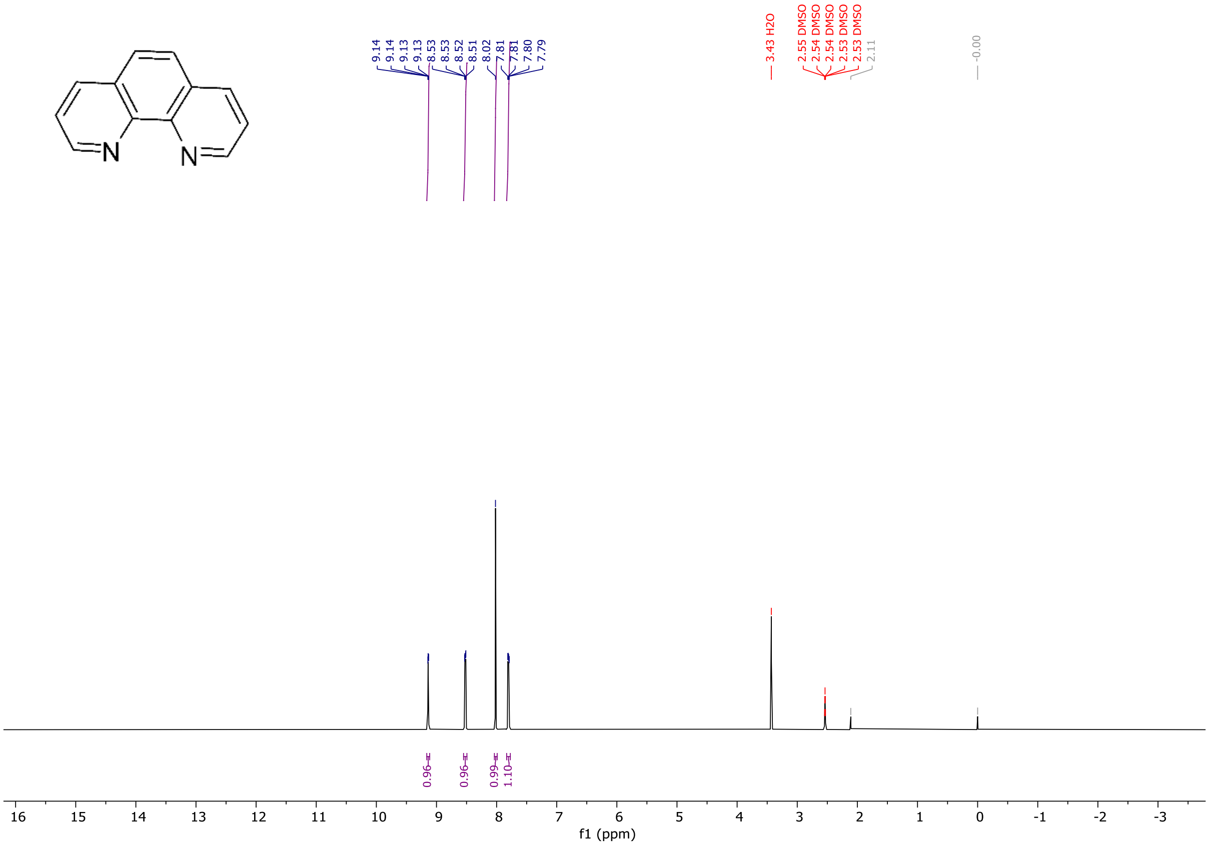

Figure S5. ^{1}H NMR (500 MHz, DMSO-*d6*) spectrum of **1,10-Phenanthroline (phen)**

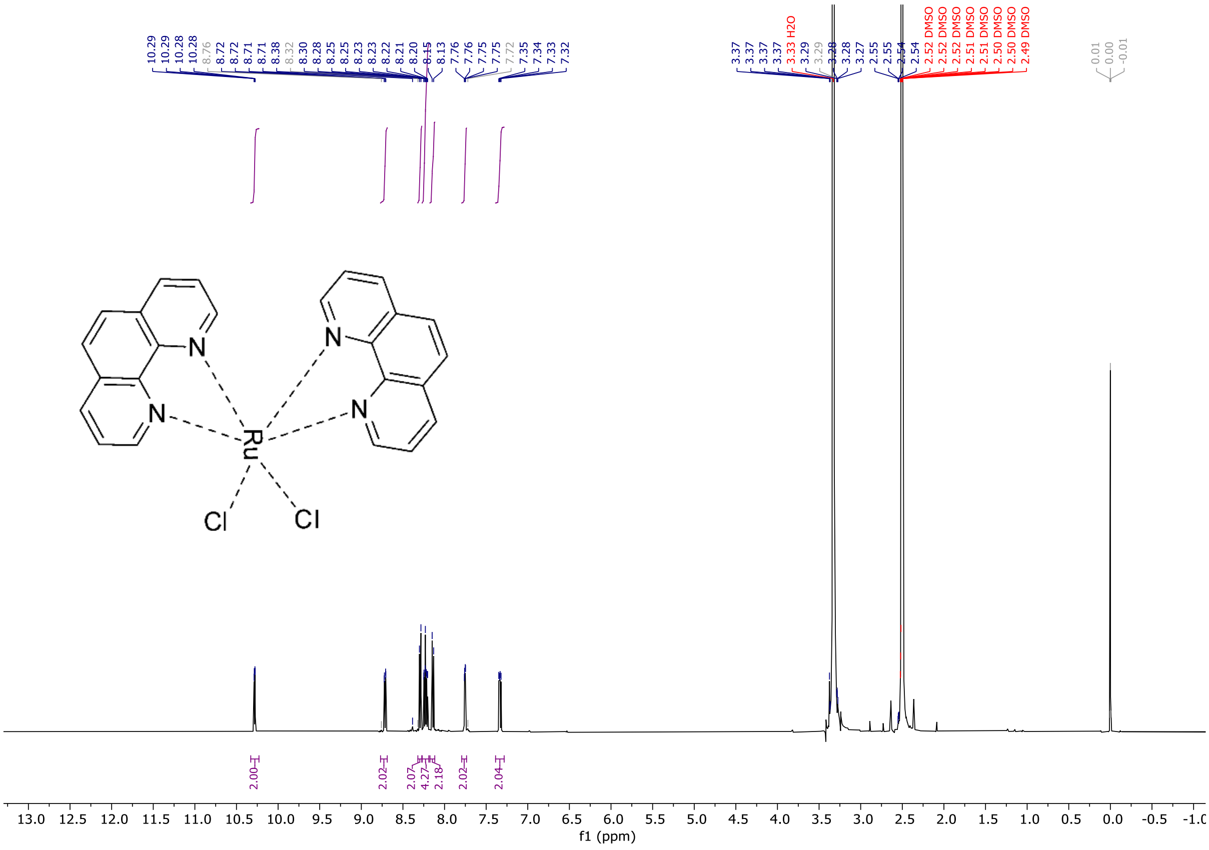

Figure S6. ^{1}H NMR (500 MHz, DMSO-*d6*) spectrum of **Ru(phen)$_2$Cl$_2$**

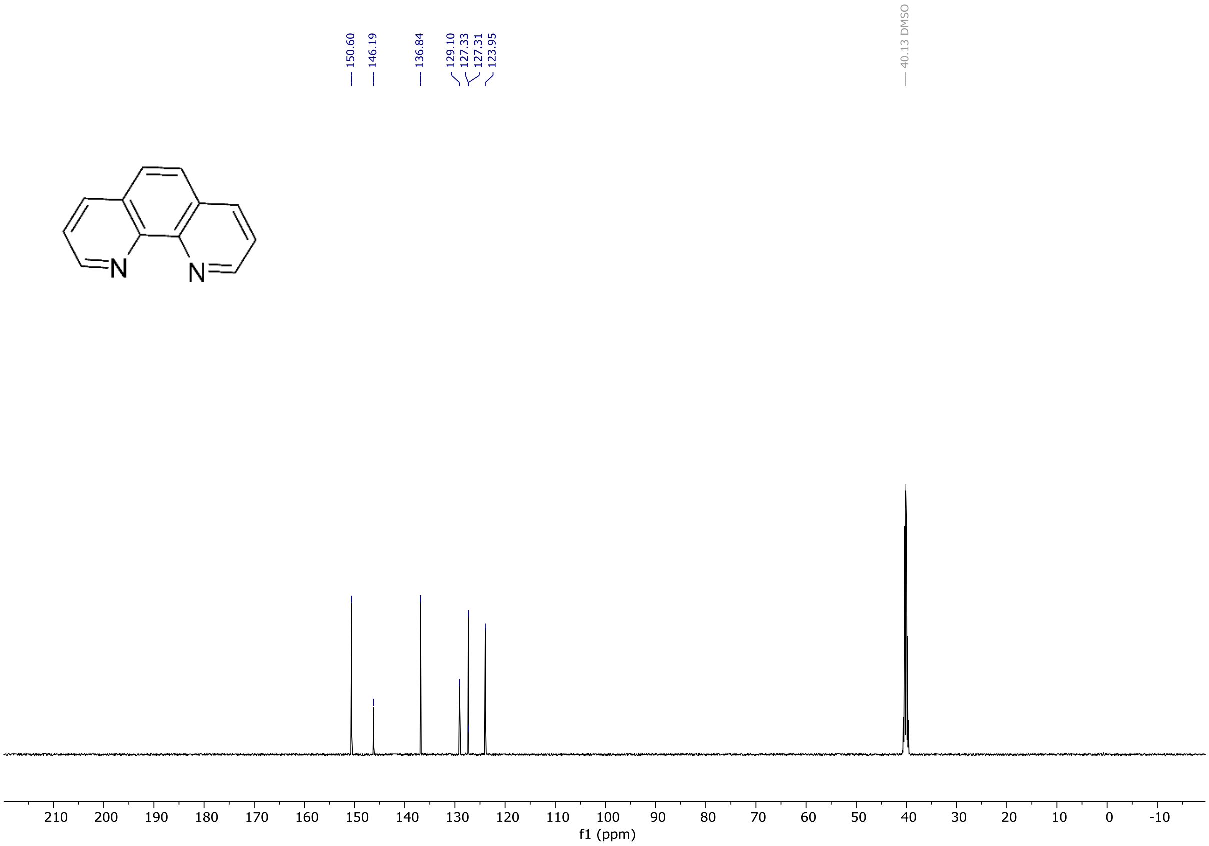

Figure S7. ^{13}C NMR (125 MHz, DMSO-*d6*) spectrum of **1,10-Phenanthroline (phen)**

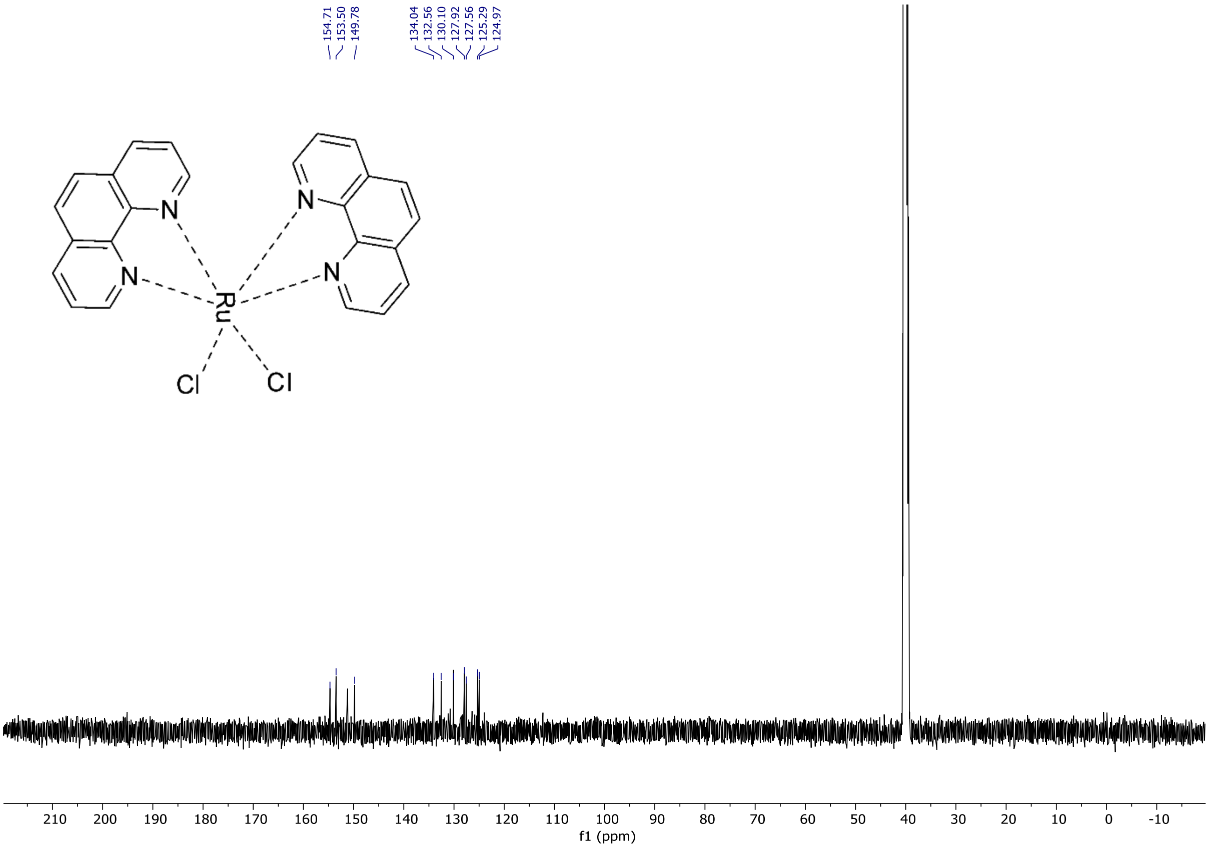

Figure S8. ^{13}C NMR (125 MHz, DMSO-*d6*) spectrum of **$Ru(phen)_2Cl_2$**

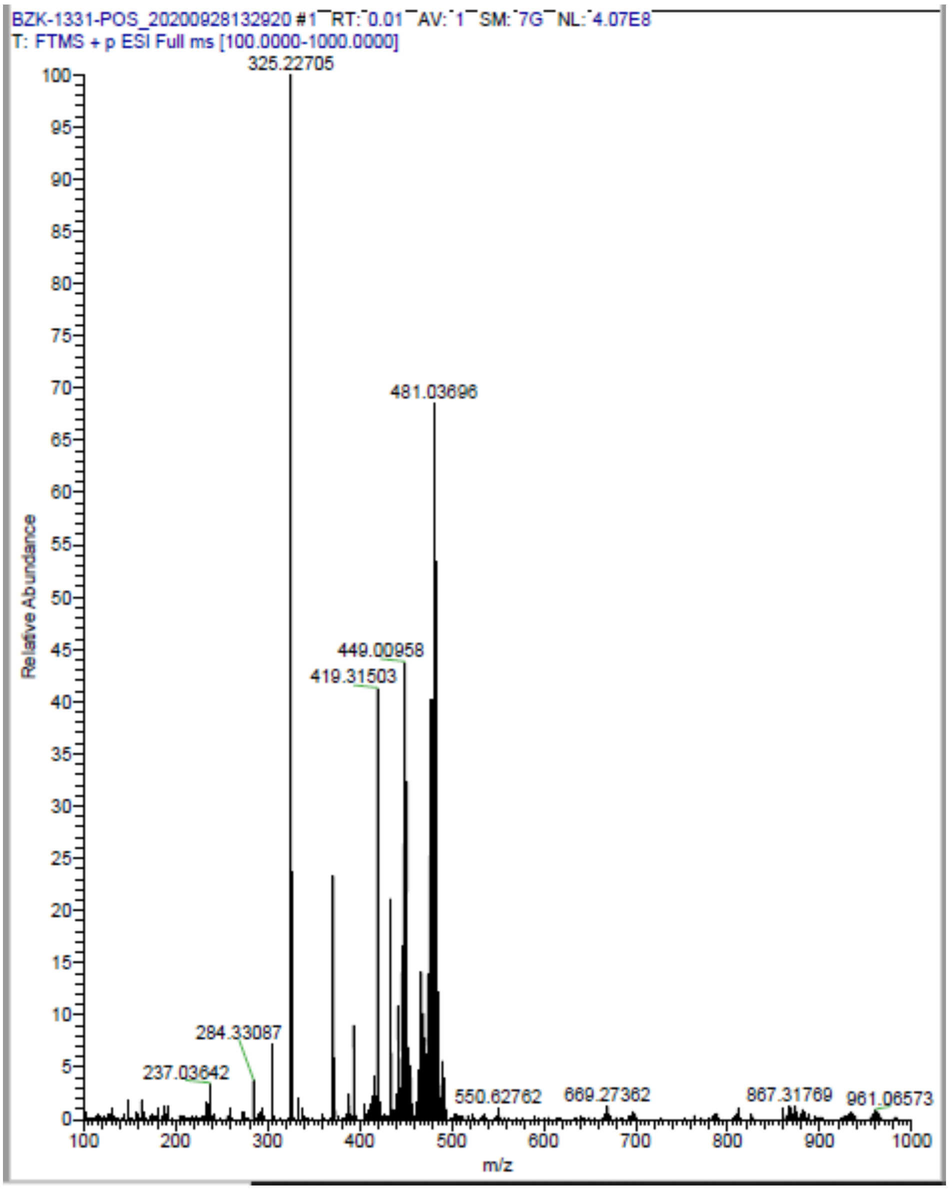

Figure S9. Mass spectrum of **$Ru(bpy)_2Cl_2$**

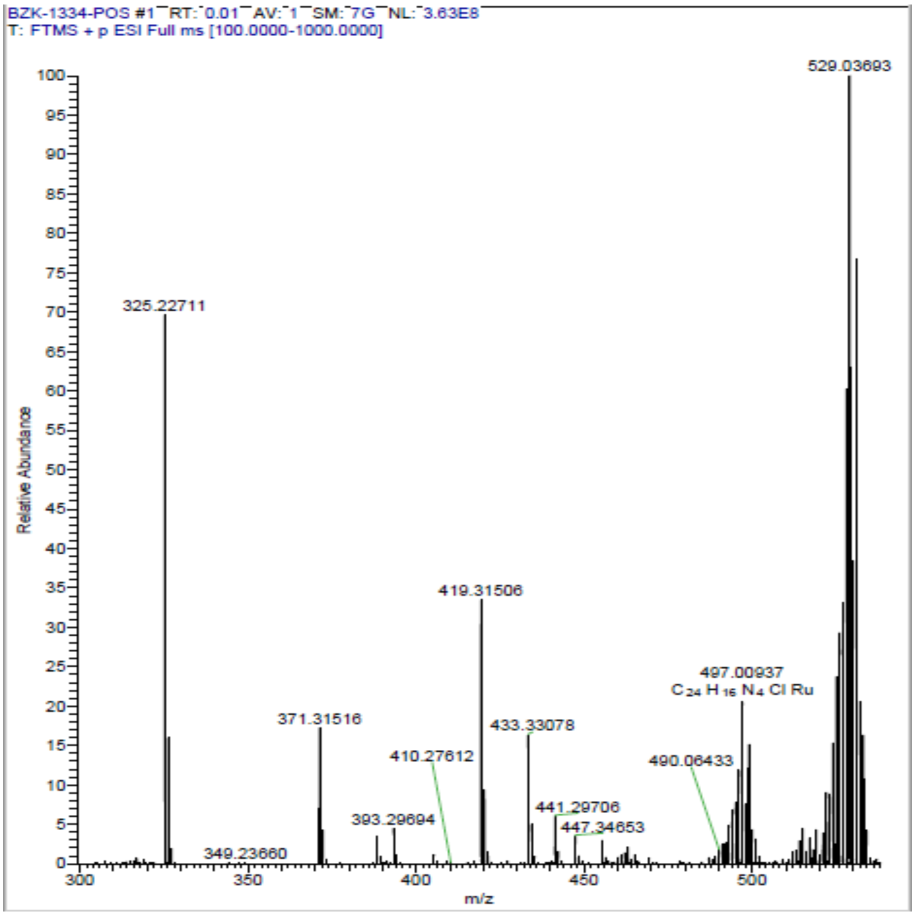

Figure S10. Mass spectrum of **Ru(phen)$_2$Cl$_2$**

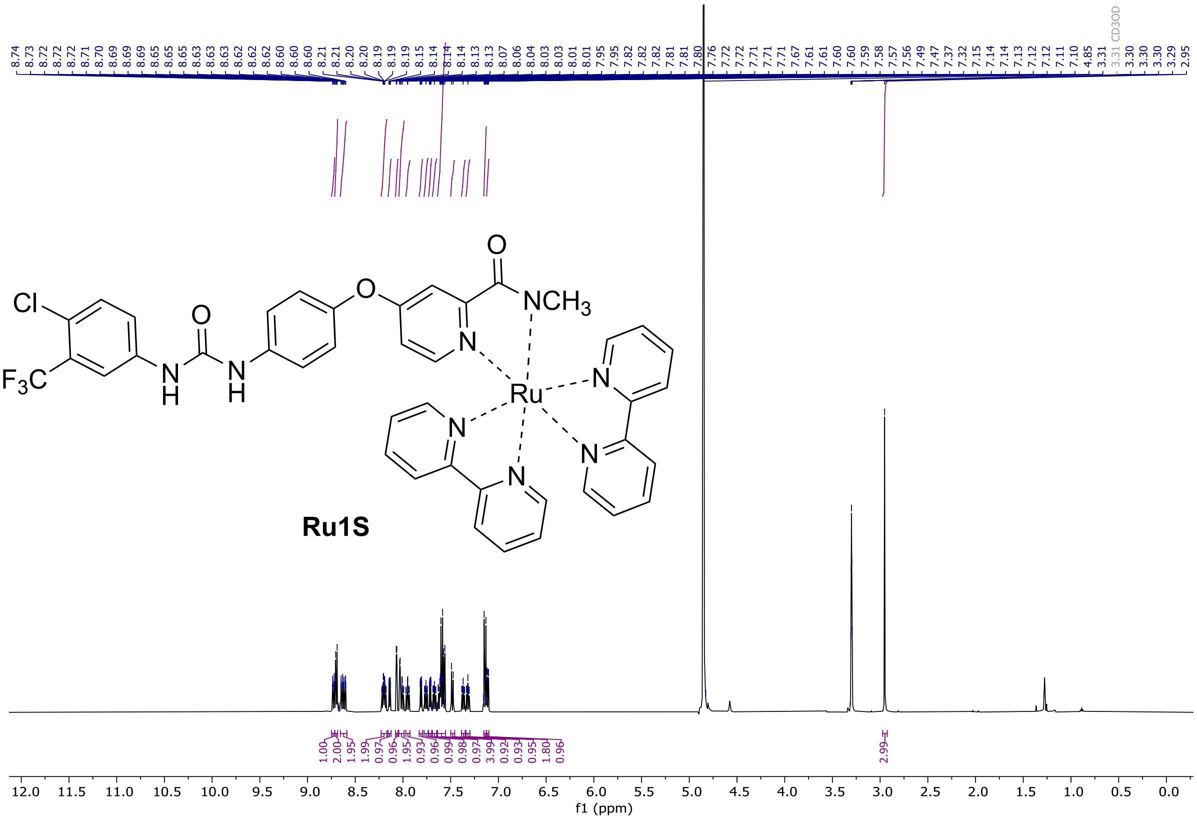

Figure S11. ^{1}H NMR (500 MHz, CD_3OD) spectrum of **Ru1S**

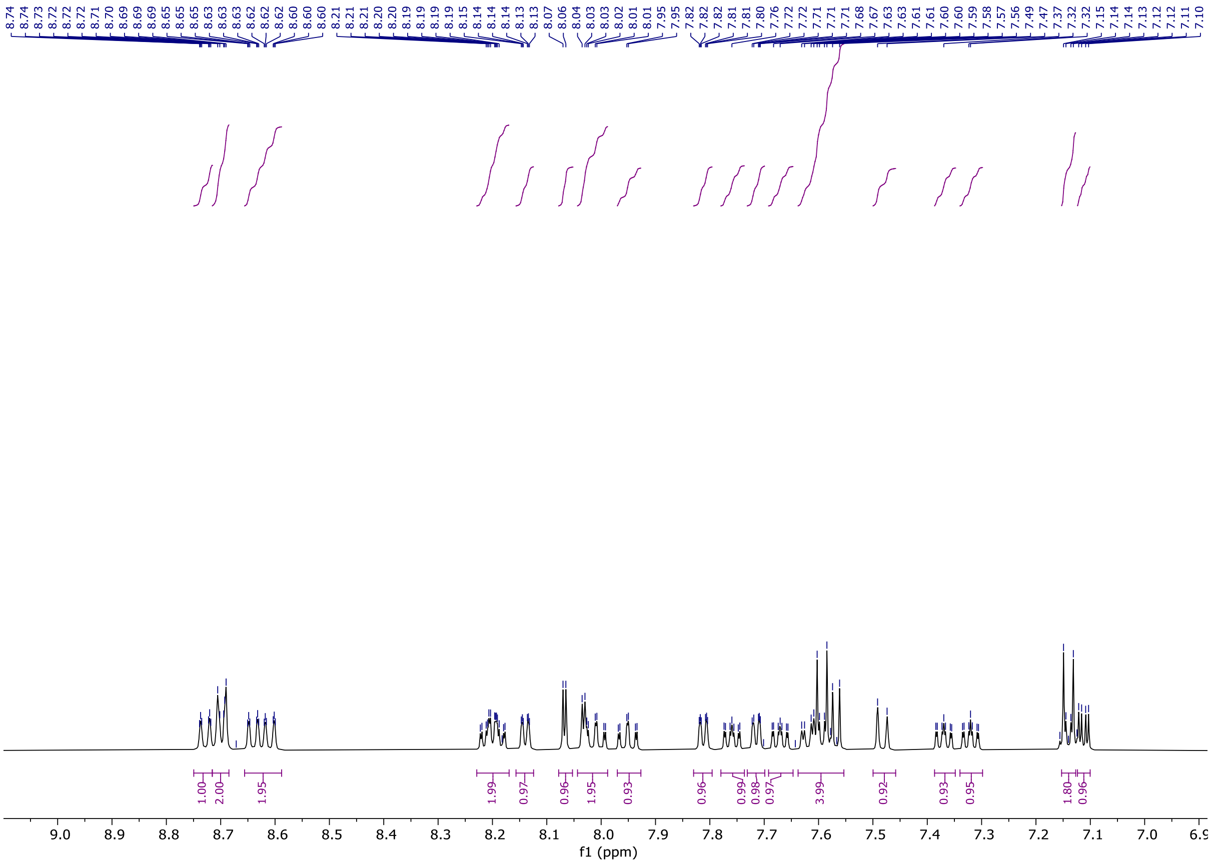

Figure S12. Extended aromatic region ^{1}H NMR (500 MHz, CD_3OD) spectrum of **Ru1S**

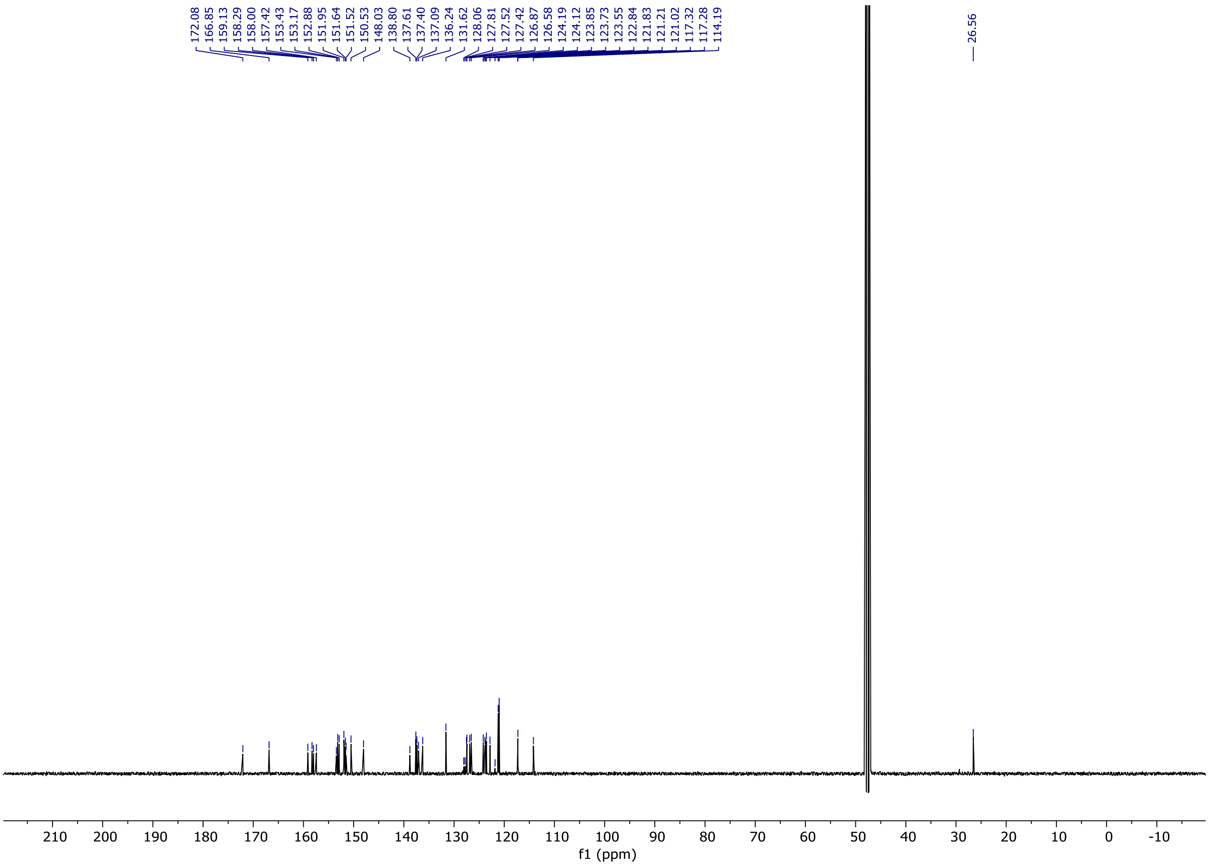

Figure S13. ^{13}C NMR (125 MHz, CD_3OD) spectrum of **Ru1S**

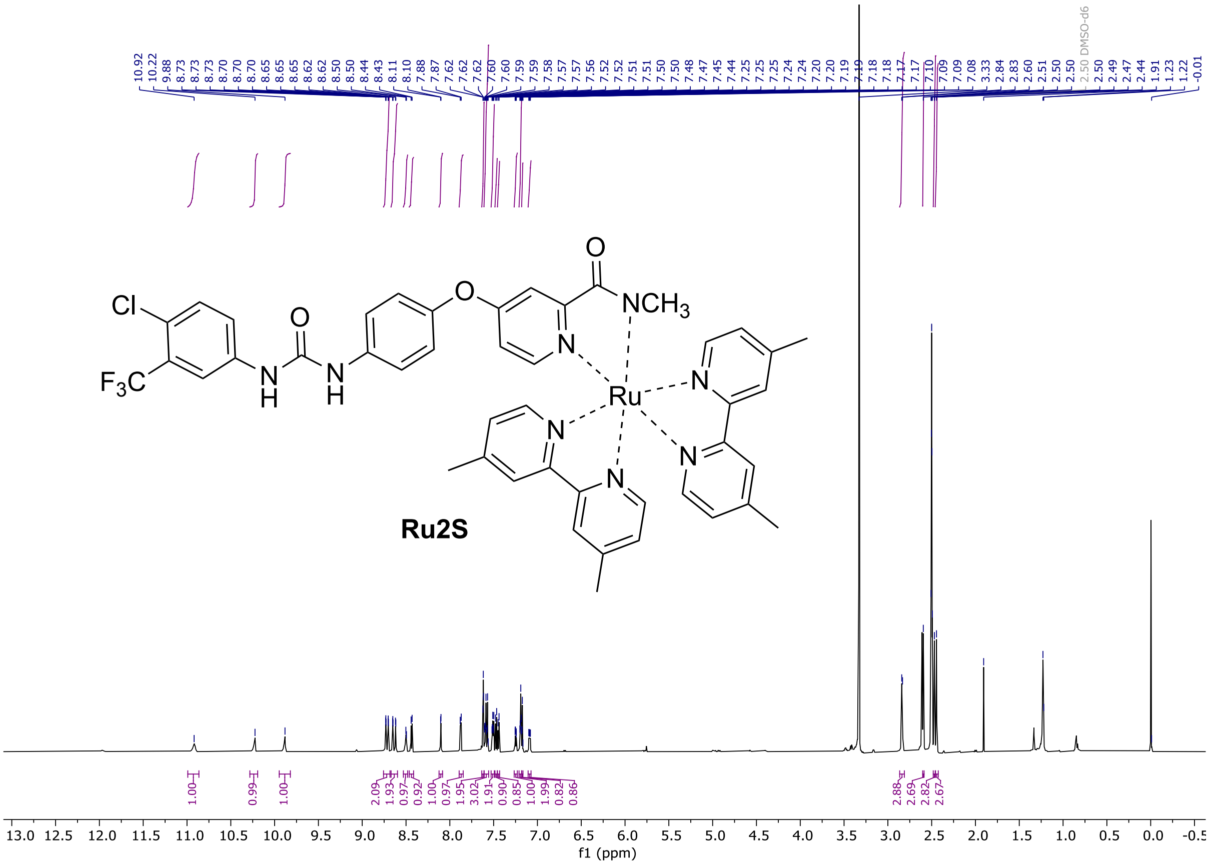

Figure S14. ^{1}H NMR (500 MHz, DMSO-*d6*) spectrum of **Ru2S**

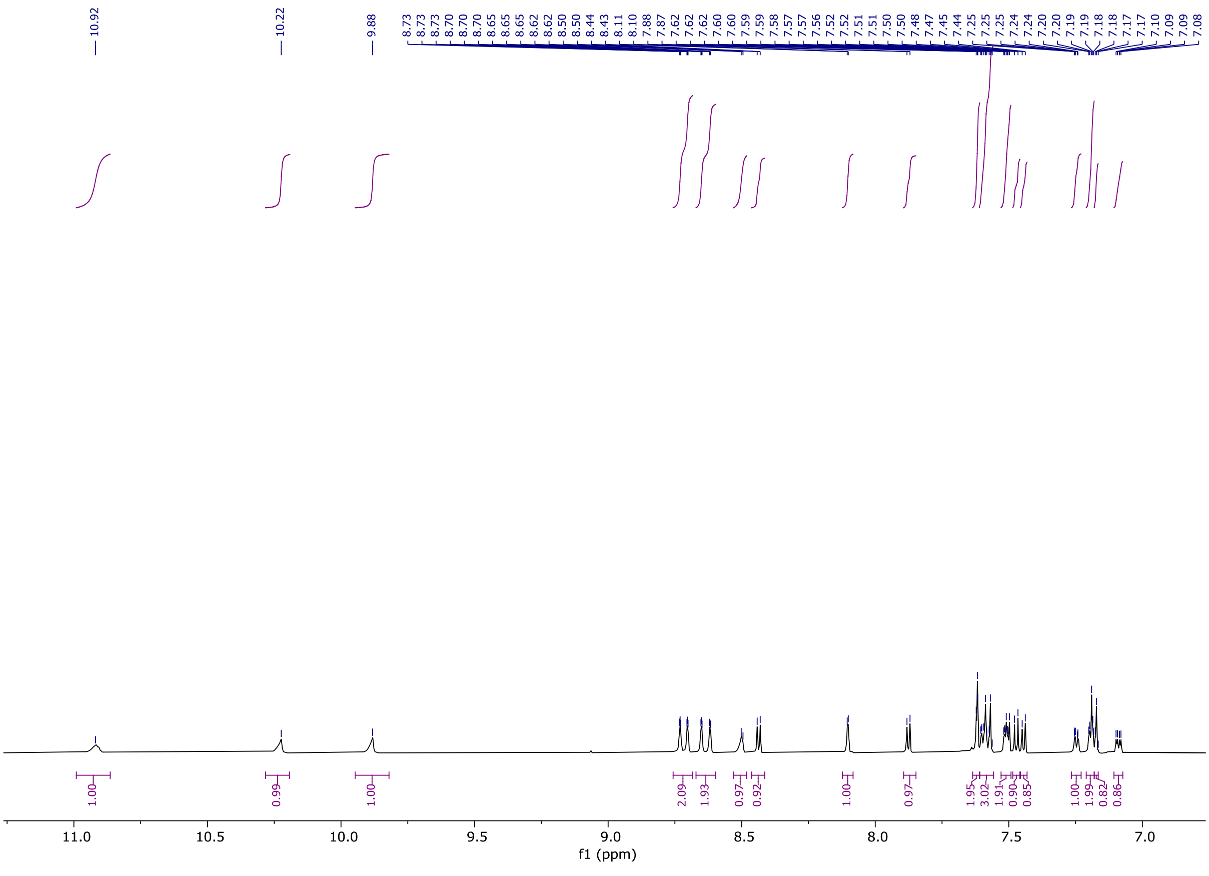

Figure S15. Extended aromatic region ^{1}H NMR (500 MHz, DMSO-*d6*) spectrum of **Ru2S**

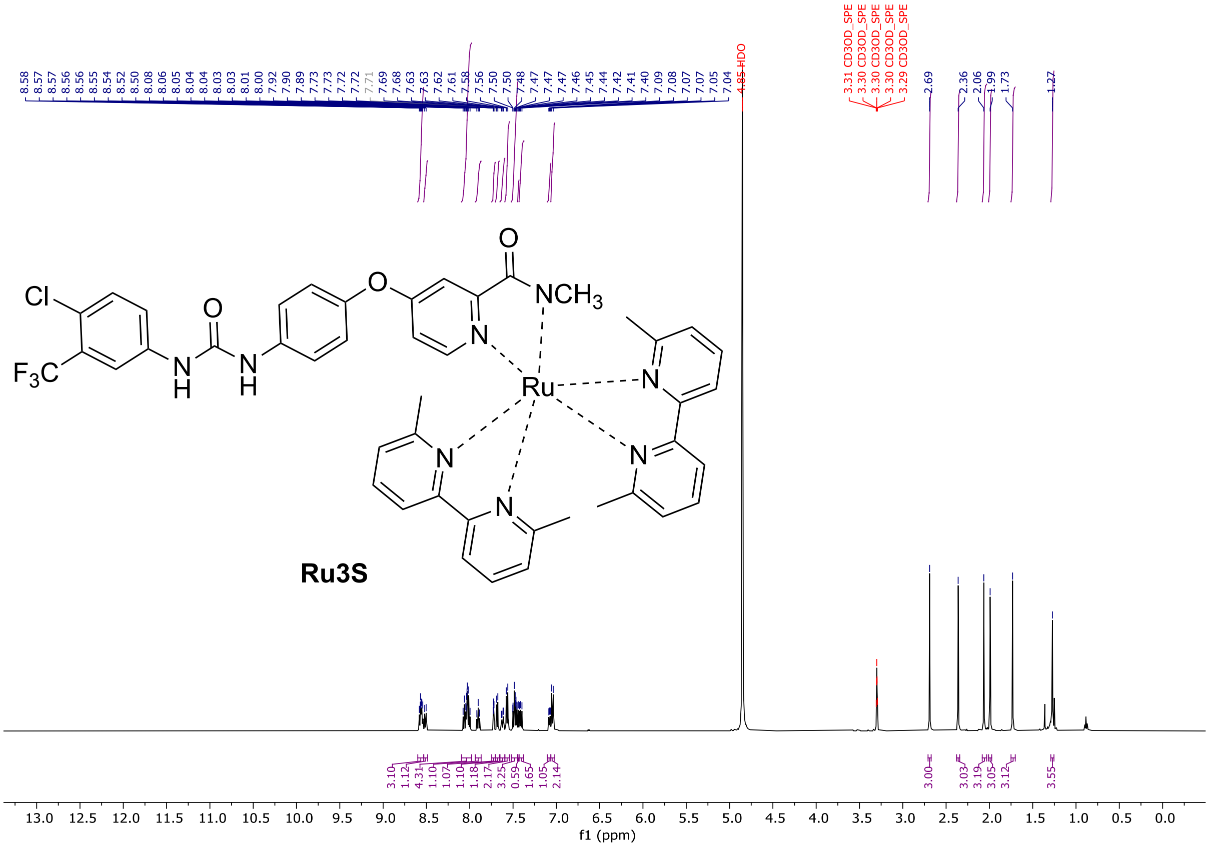

Figure S16. ^{1}H NMR (500 MHz, CD_3OD) spectrum of **Ru3S**

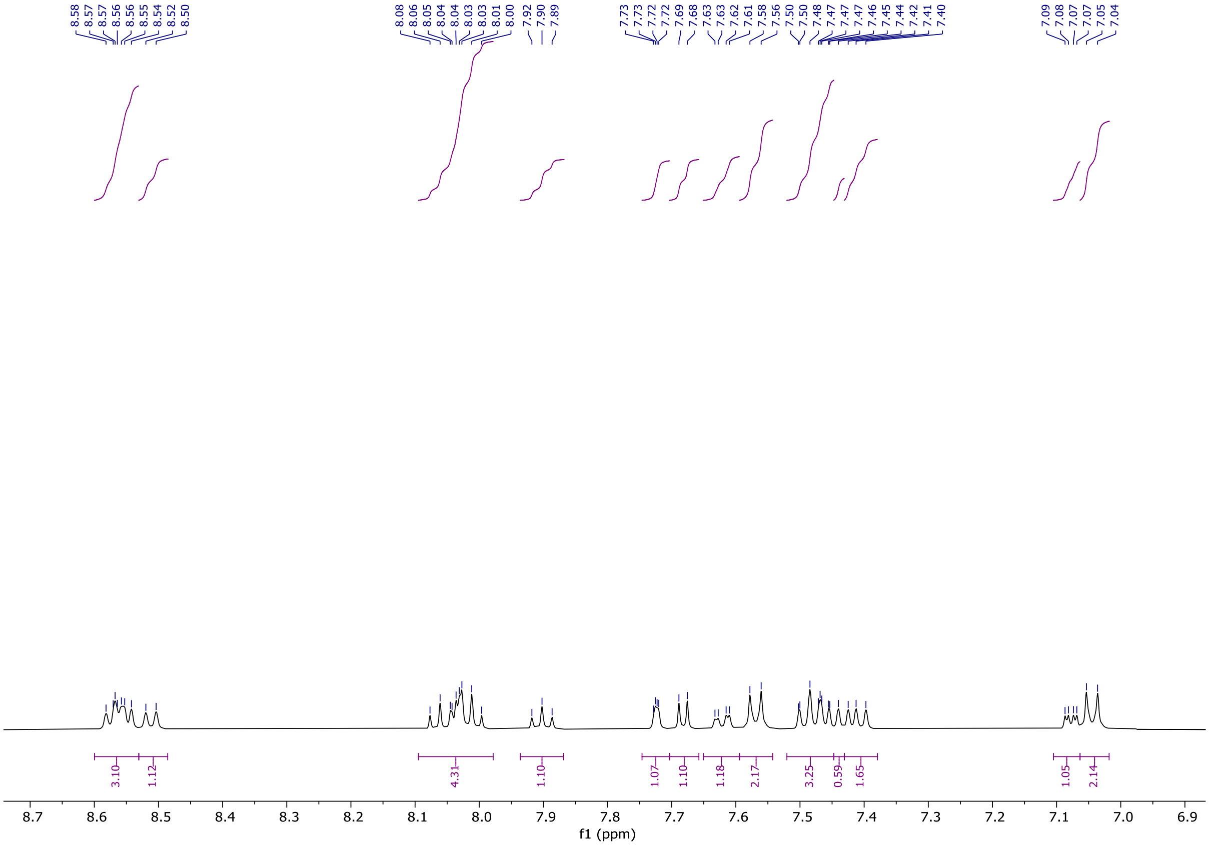

Figure S17. Extended aromatic region ^{1}H NMR (500 MHz, CD_3OD) spectrum of **Ru3S**

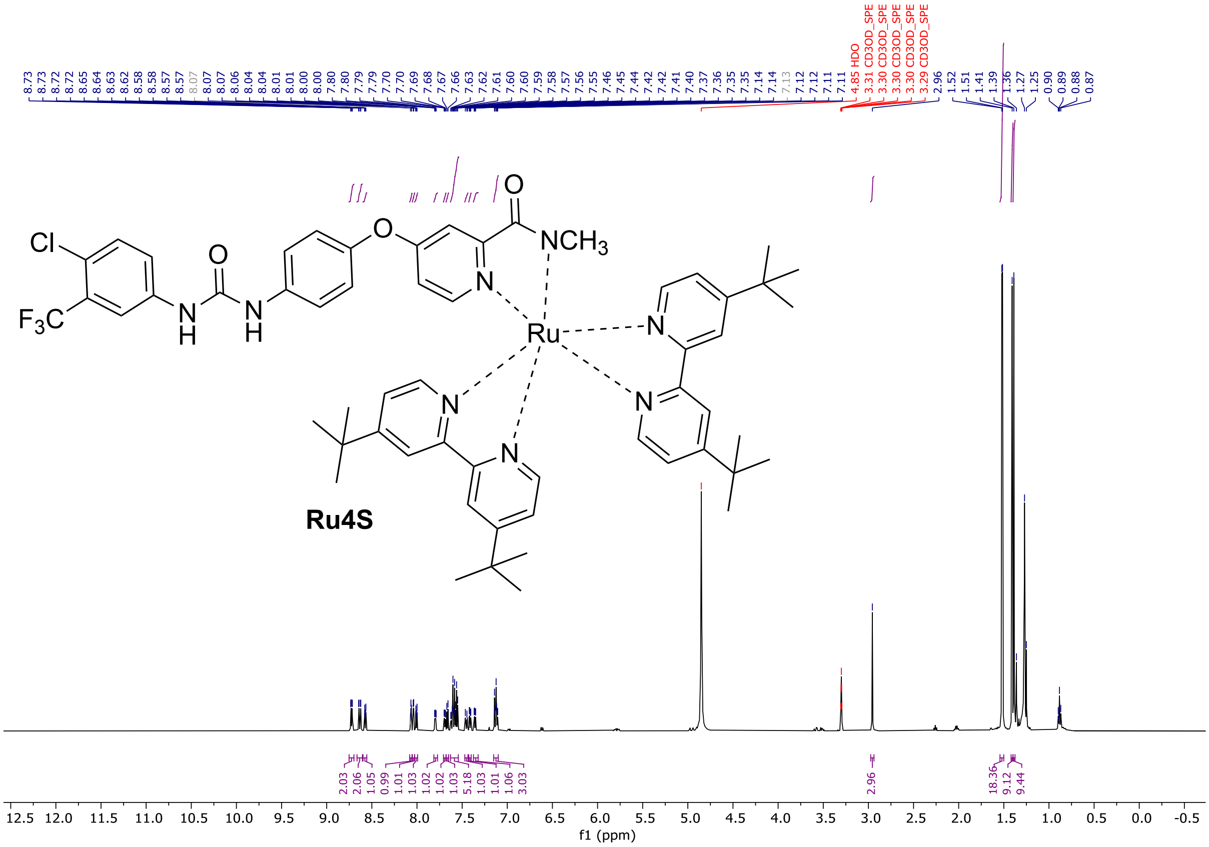

Figure S18. ^{1}H NMR (500 MHz, CD_3OD) spectrum of **Ru4S**

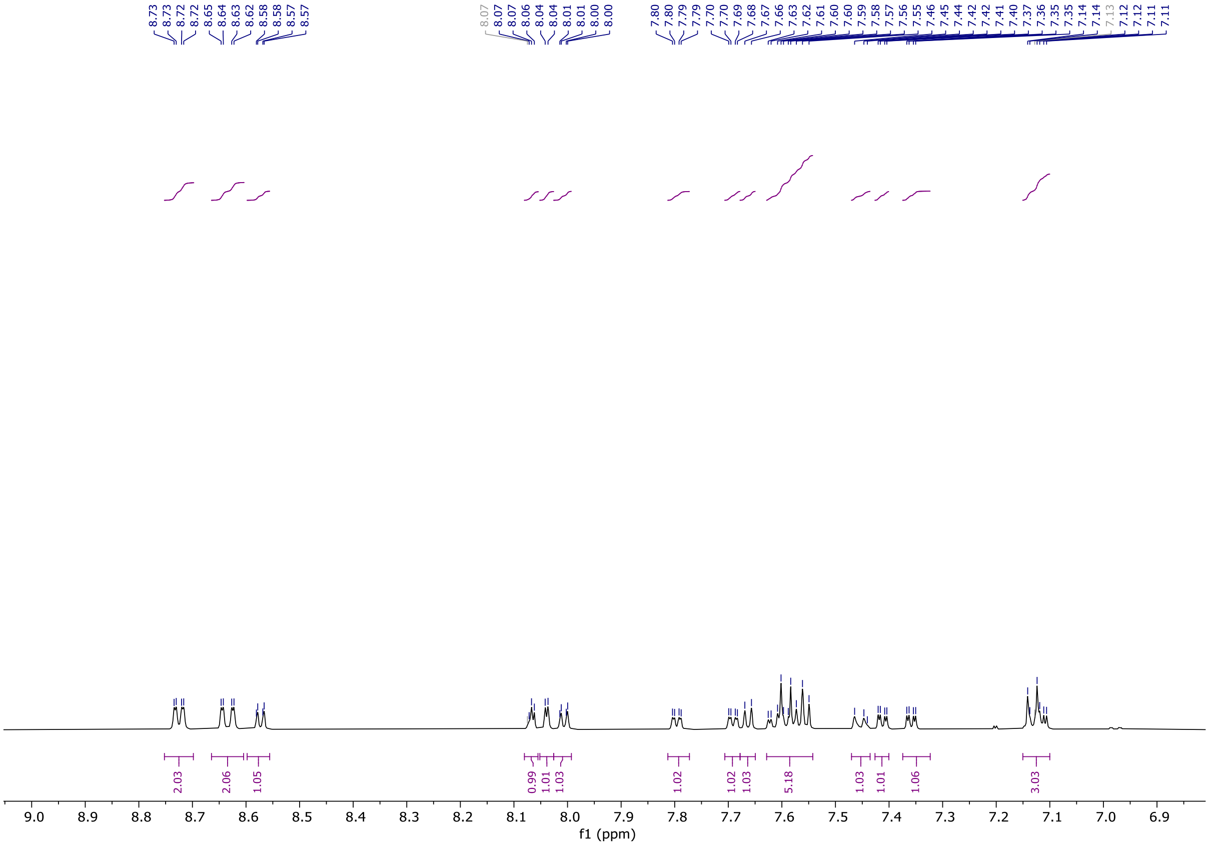

Figure S19. Extended aromatic region ^{1}H NMR (500 MHz, CD_3OD) spectrum of **Ru4S**

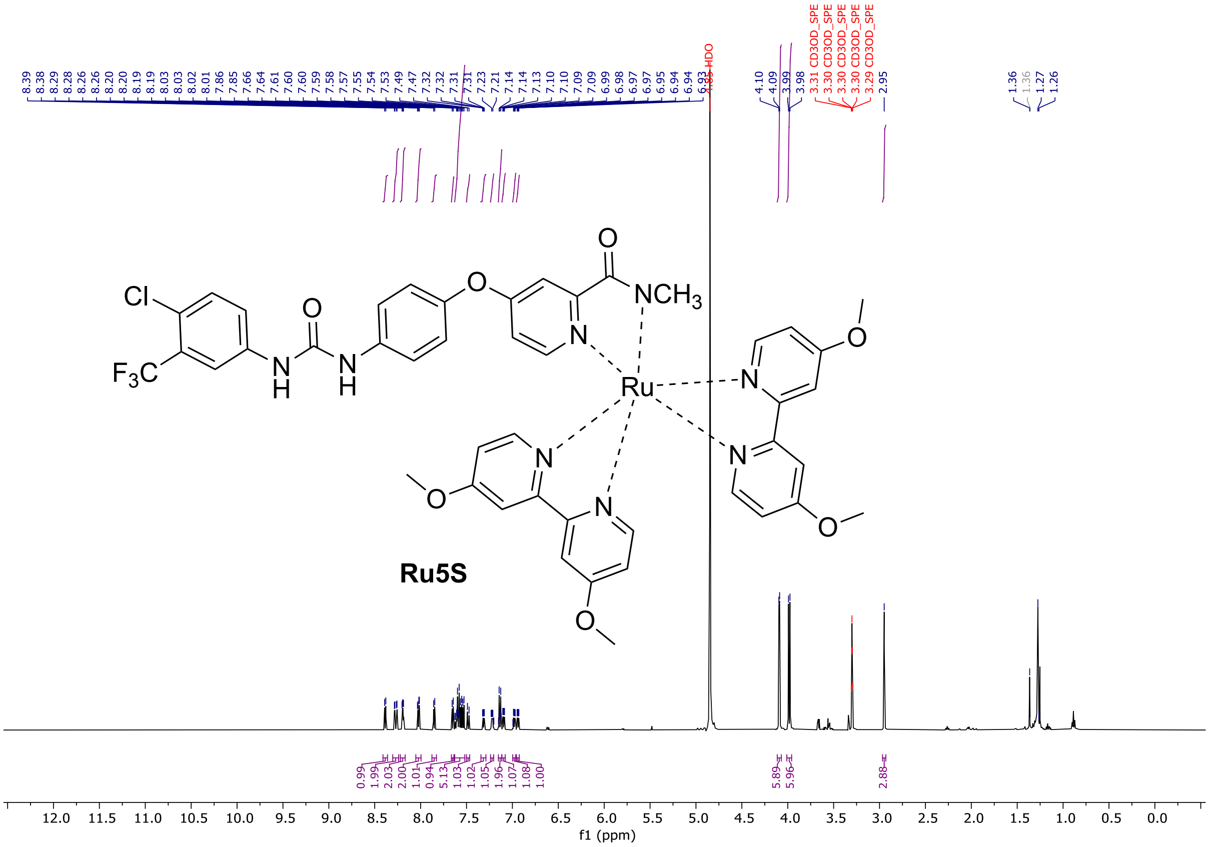

Figure S20. ^{1}H NMR (500 MHz, CD_3OD) spectrum of **Ru5S**

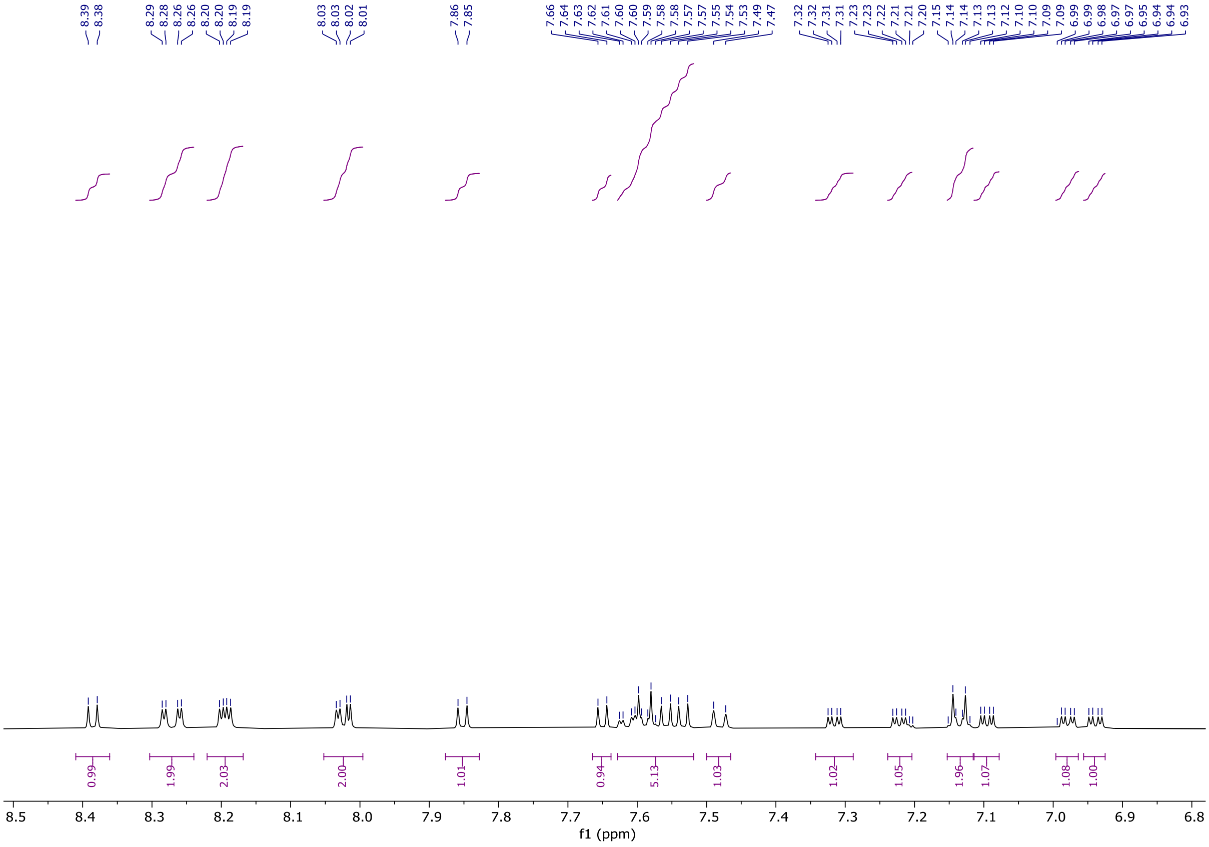

Figure S21. Extended aromatic region ^{1}H NMR (500 MHz, CD_3OD) spectrum of **Ru5S**

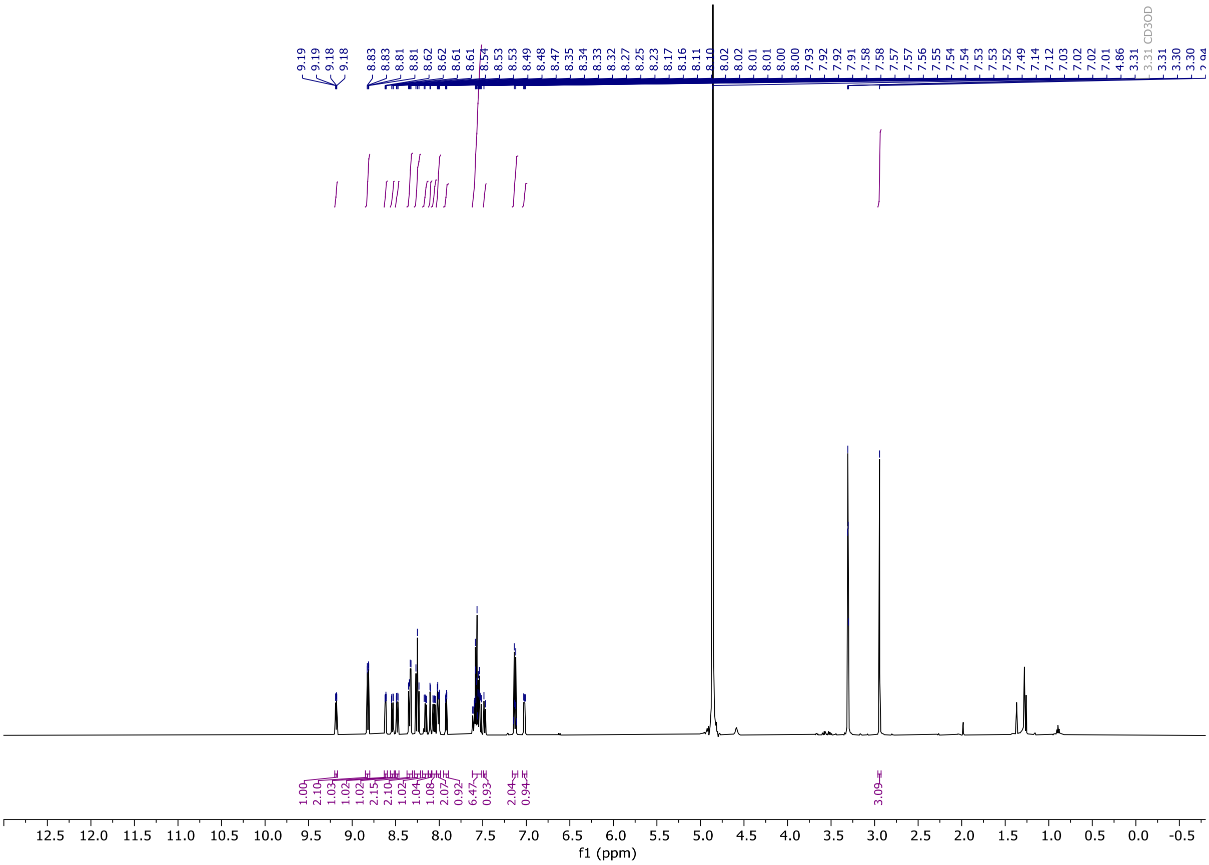

Figure S22. ^{1}H NMR (500 MHz, CD_3OD) spectrum of **Ru6S**

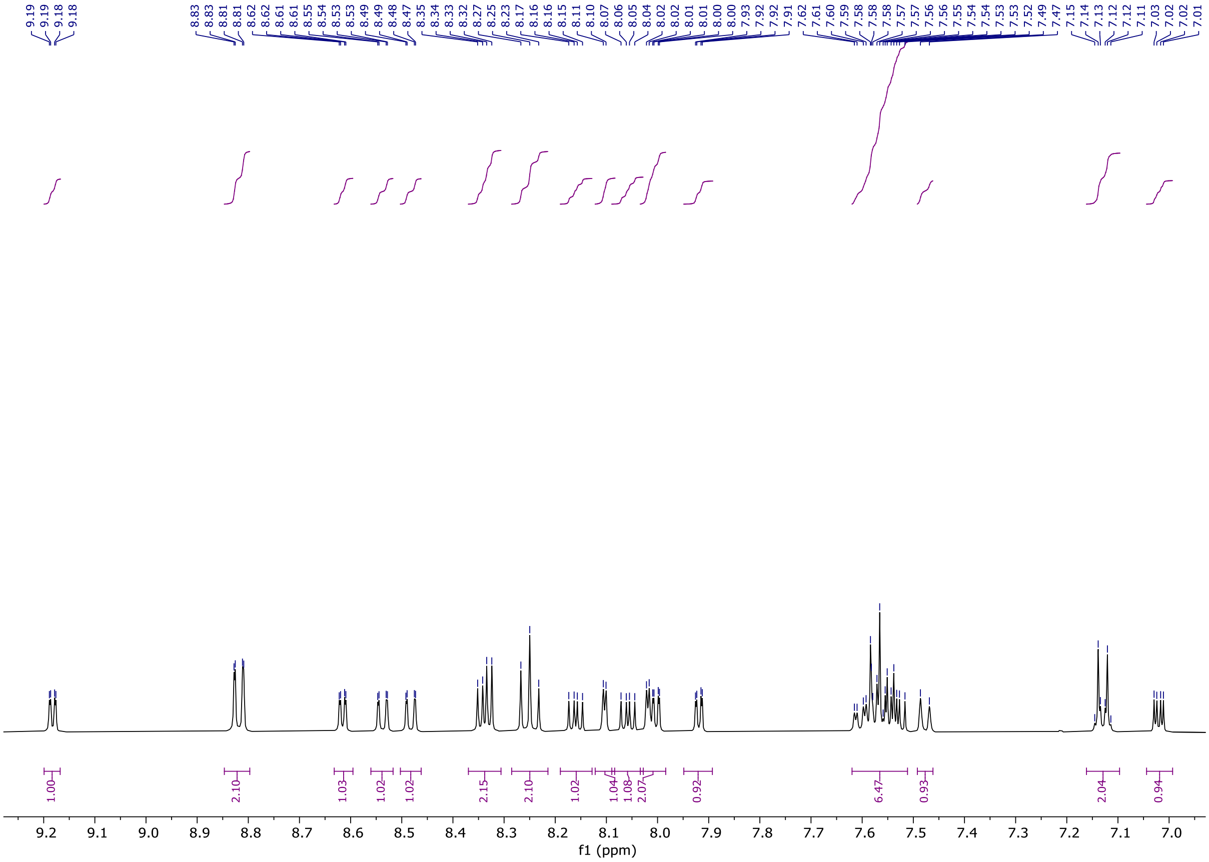

Figure S23. Extended aromatic region ^{1}H NMR (500 MHz, CD_3OD) spectrum of **Ru6S**

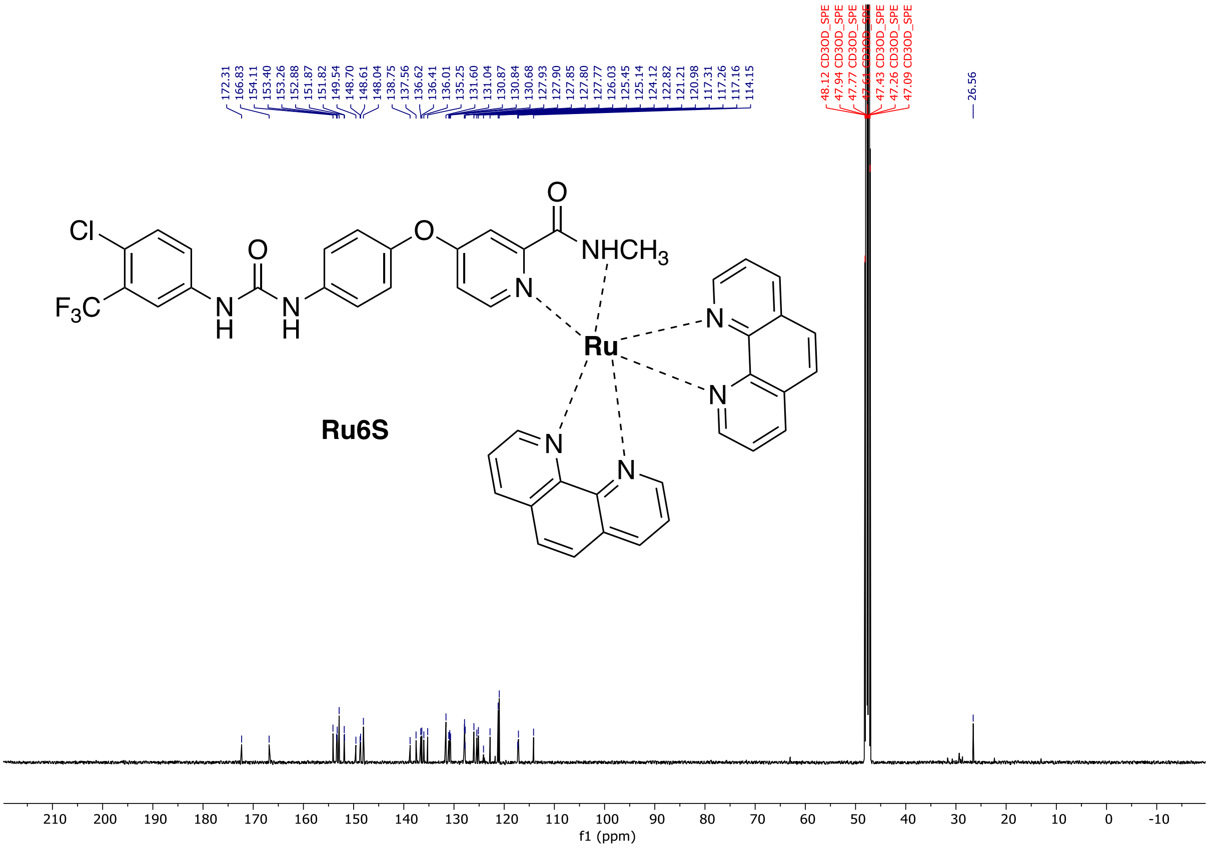

Figure S24. ^{13}C NMR (125 MHz, CD_3OD) spectrum of **Ru6S**

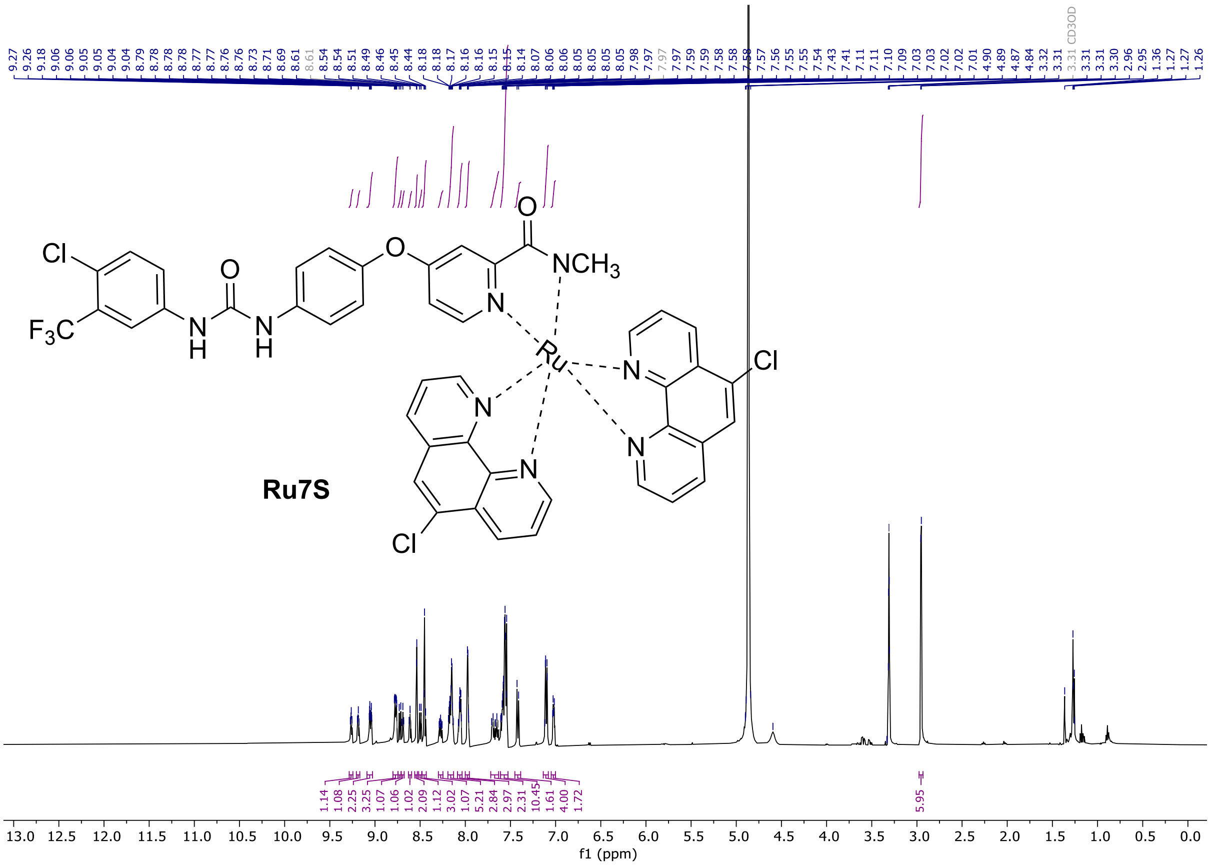

Figure S25. ^{1}H NMR (500 MHz, CD_3OD) spectrum of **Ru7S**

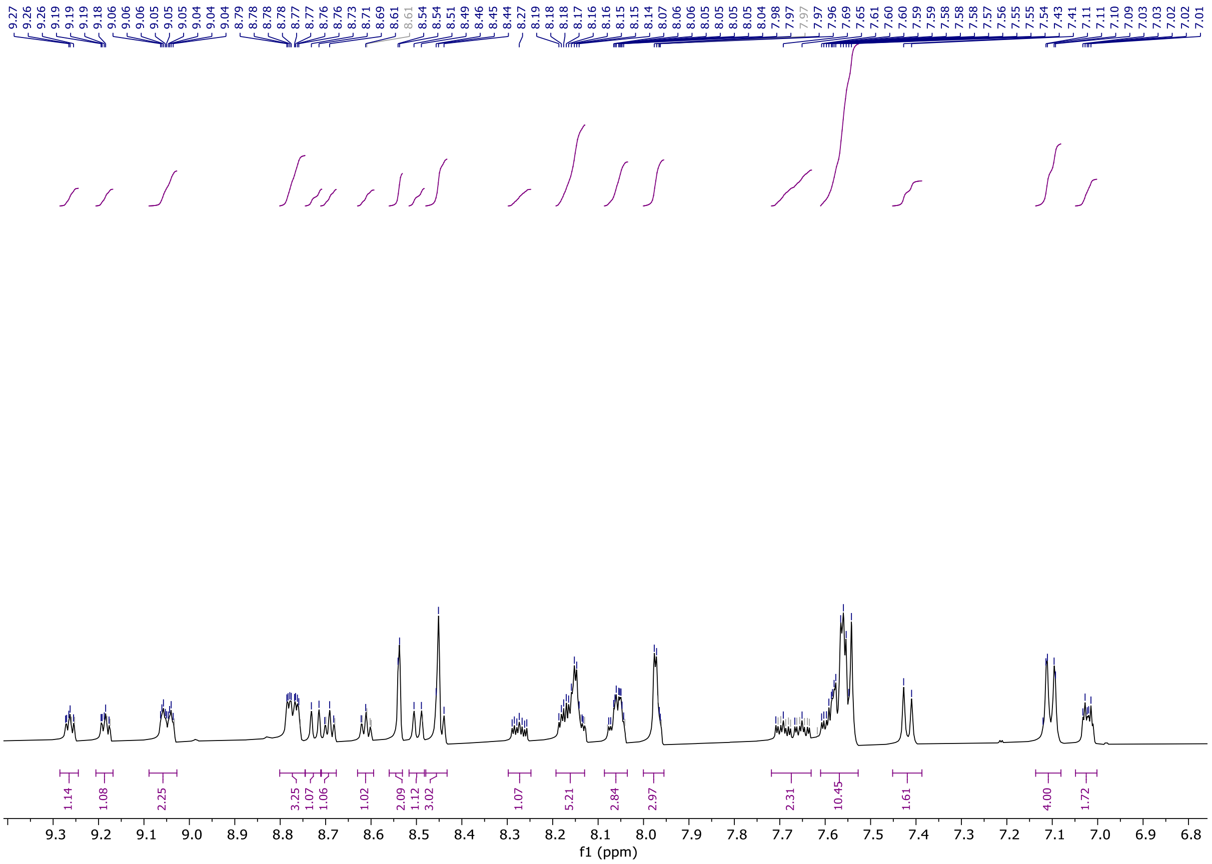

Figure S26. Extended aromatic region ^{1}H NMR (500 MHz, CD_3OD) spectrum of **Ru7S**

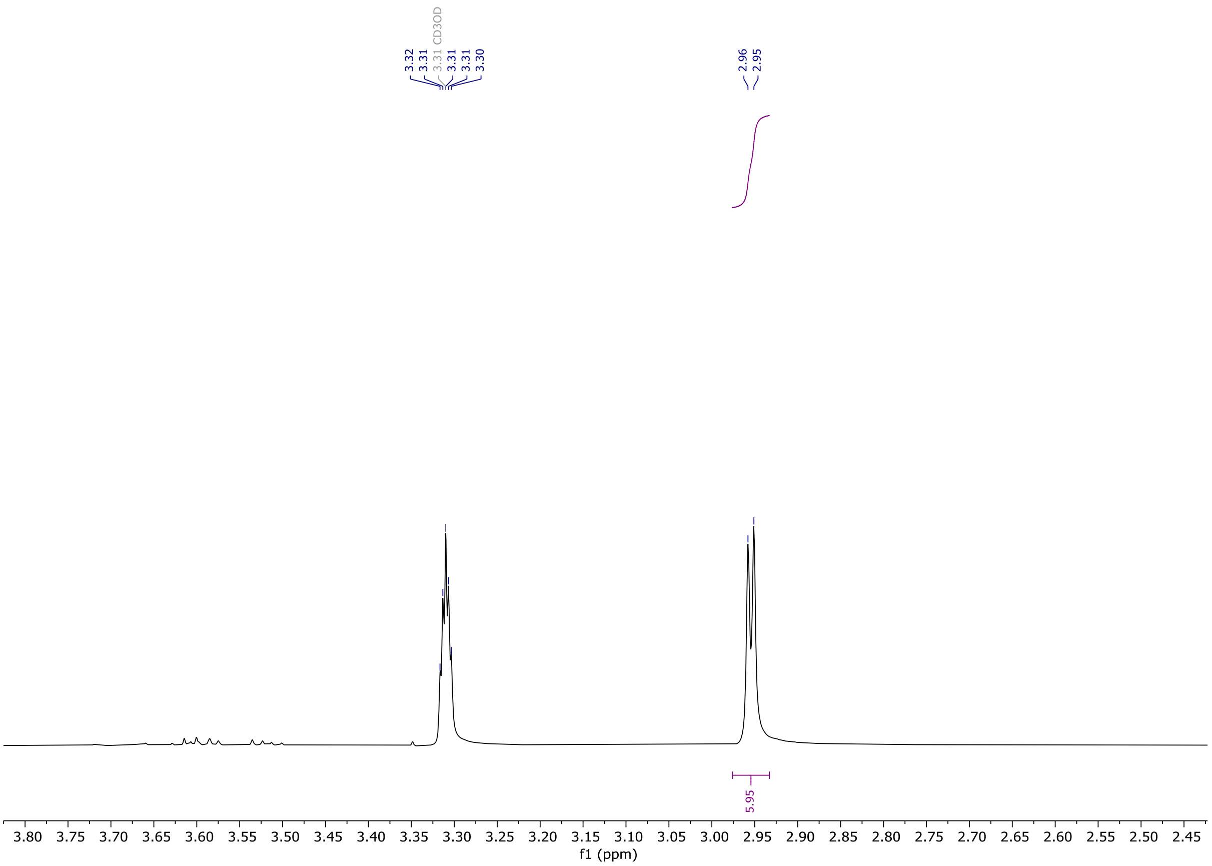

Figure S27. Extended aliphatic region ^{1}H NMR (500 MHz, CD_3OD) spectrum of **Ru7S**

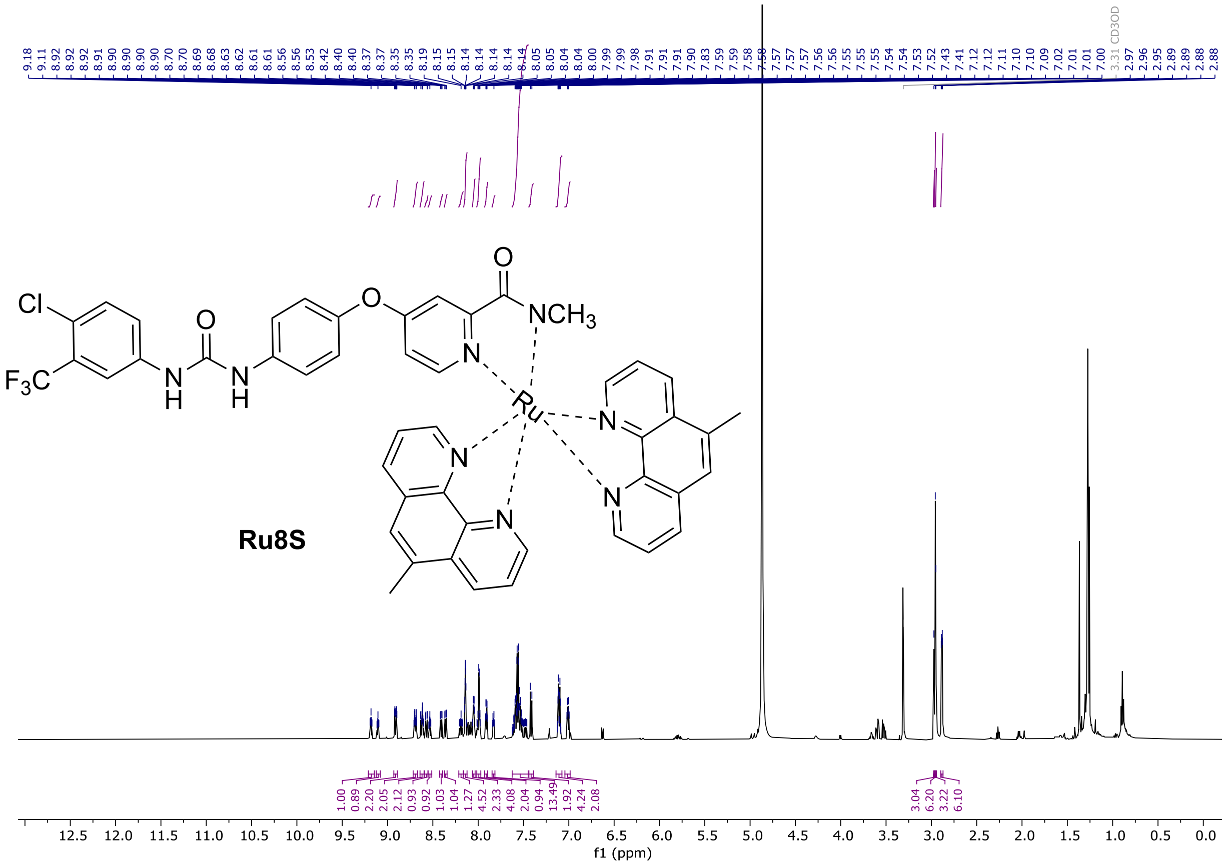

Figure S28. ^1H NMR (500 MHz, CD_3OD) spectrum of **Ru8S**

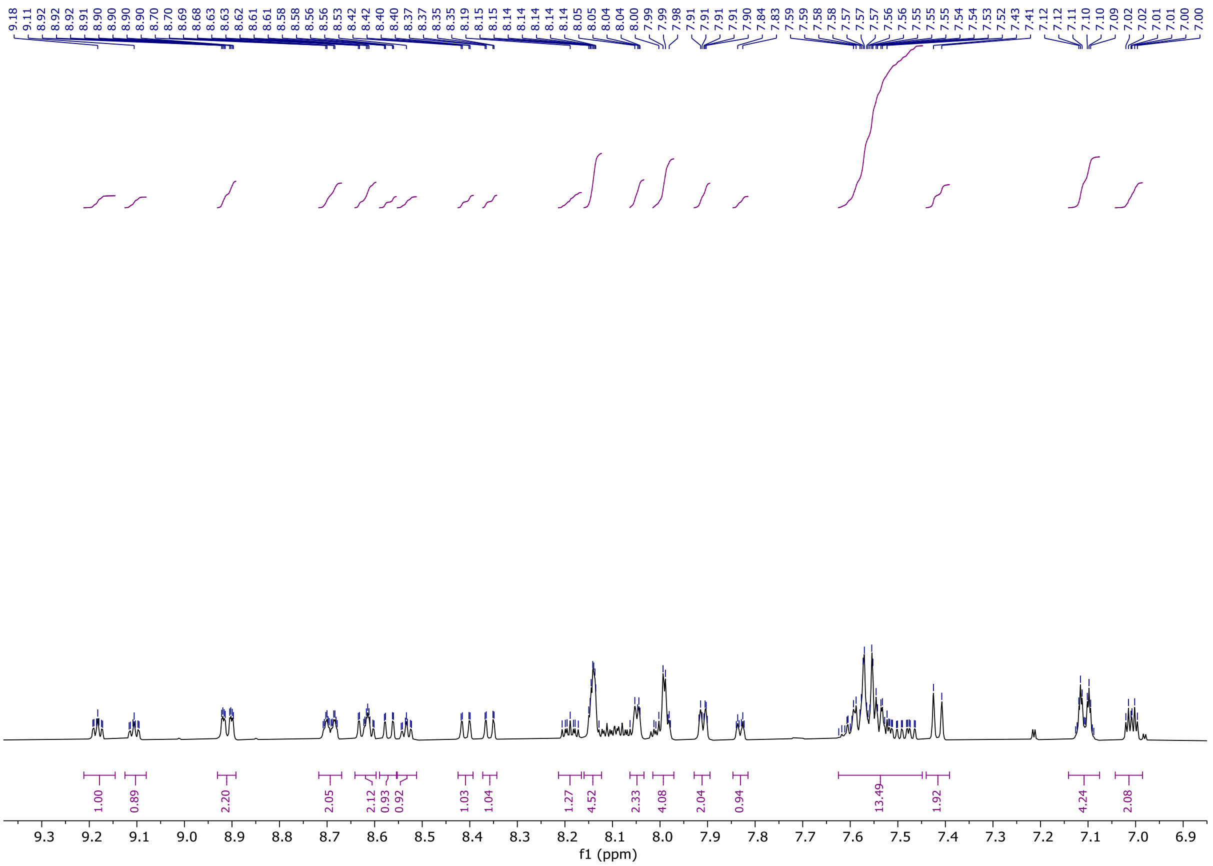

Figure S29. Extended aromatic region ^1H NMR (500 MHz, CD_3OD) spectrum of **Ru8S**

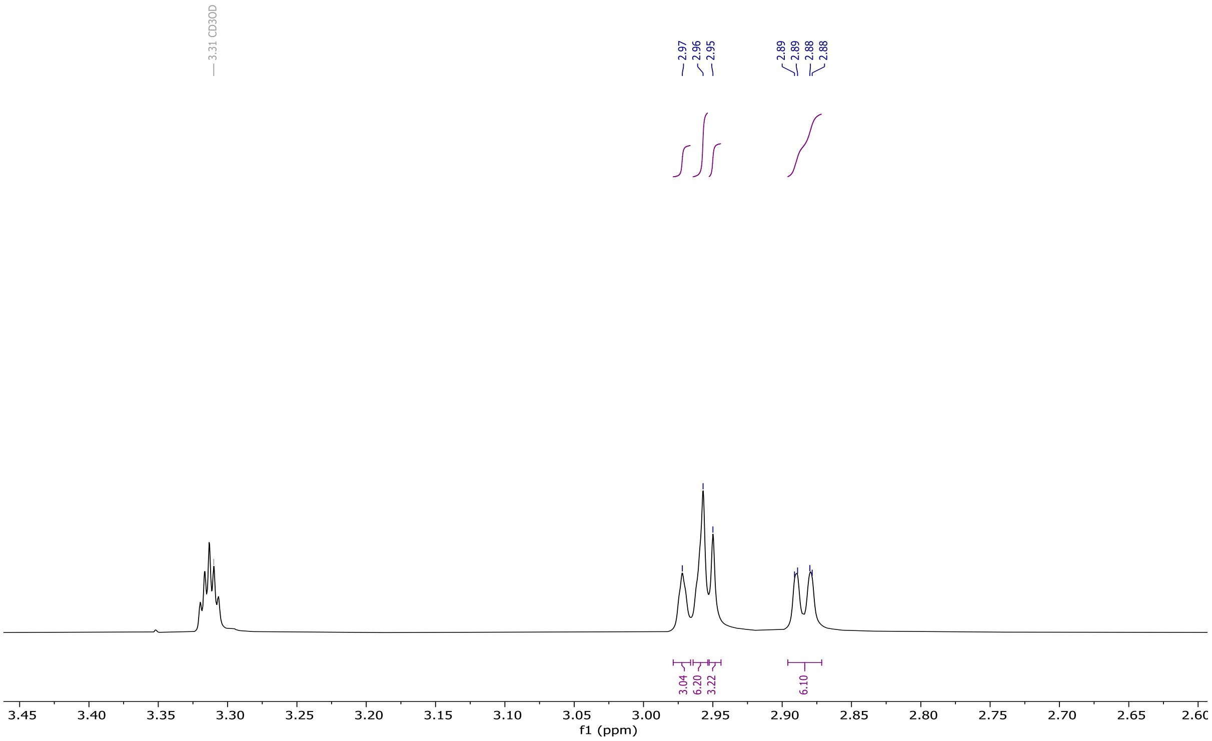

Figure S30. Extended aliphatic region ^{1}H NMR (500 MHz, CD_3OD) spectrum of **Ru8S**

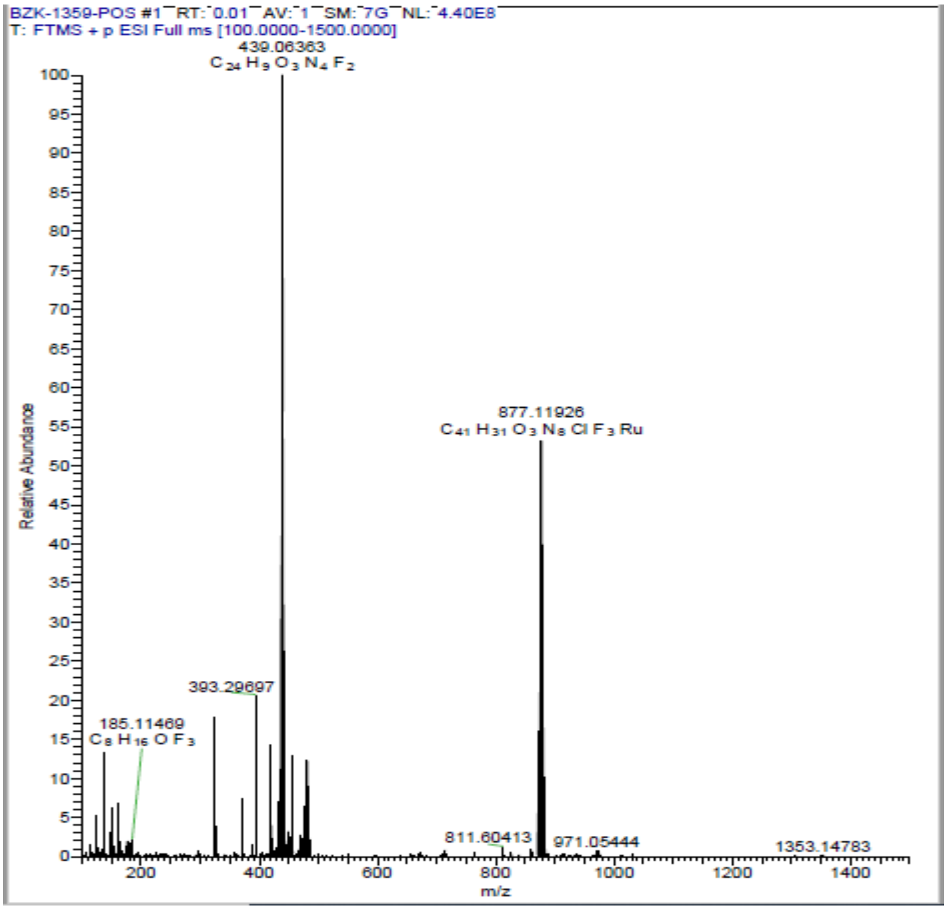

Figure S31. Mass spectrum of **Ru1S**

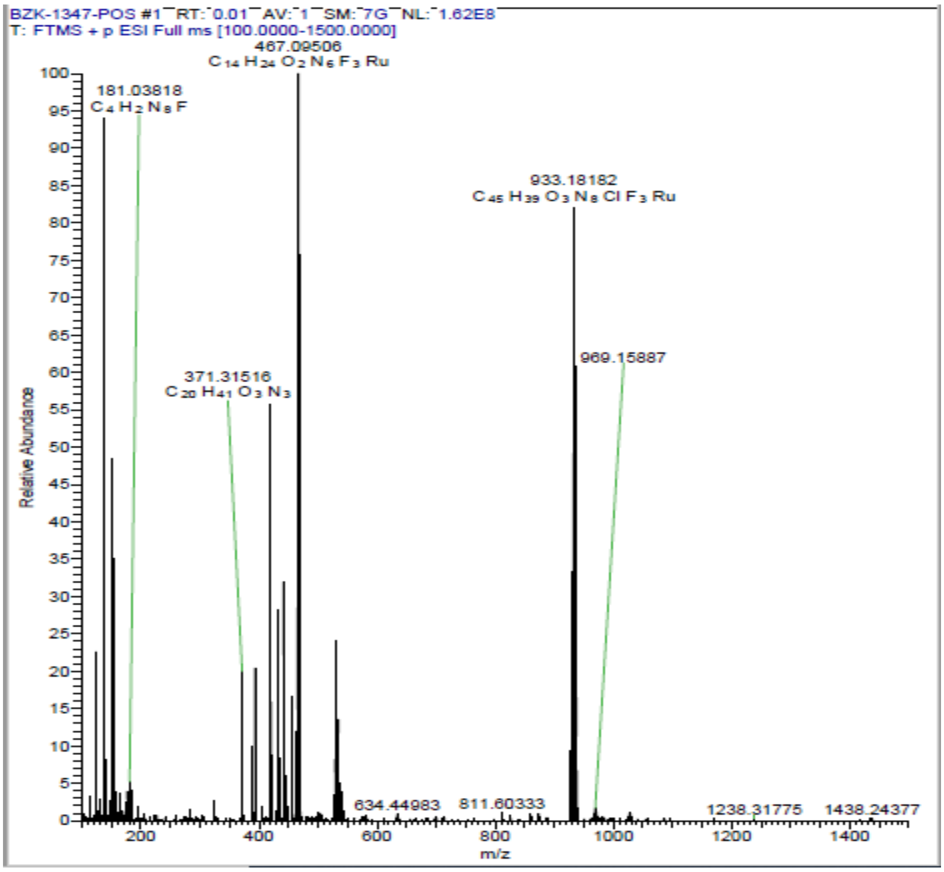

Figure S32. Mass spectrum of **Ru2S**

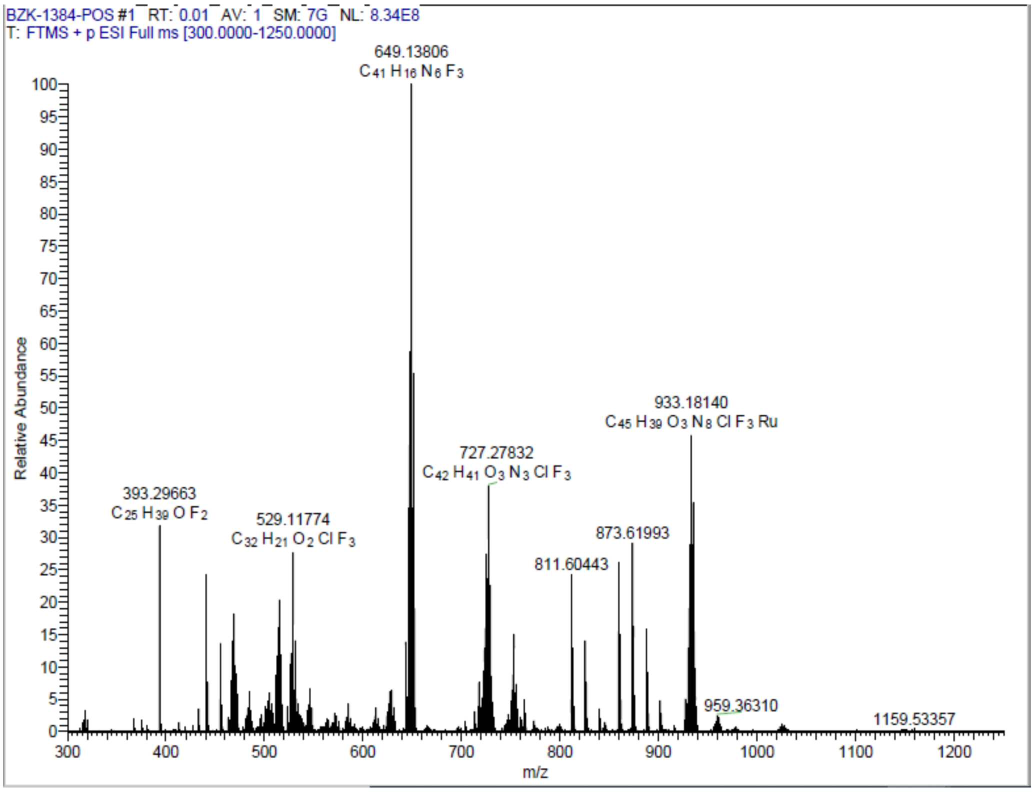

Figure S33. Mass spectrum of **Ru3S**

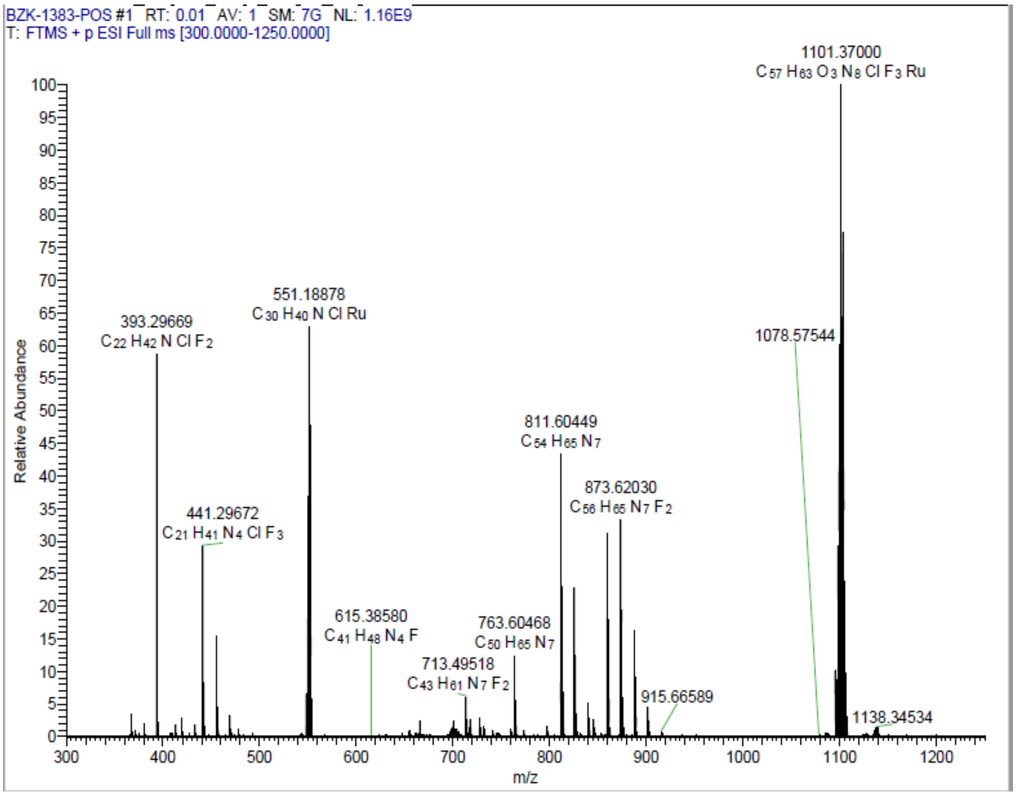

Figure S34. Mass spectrum of **Ru4S**

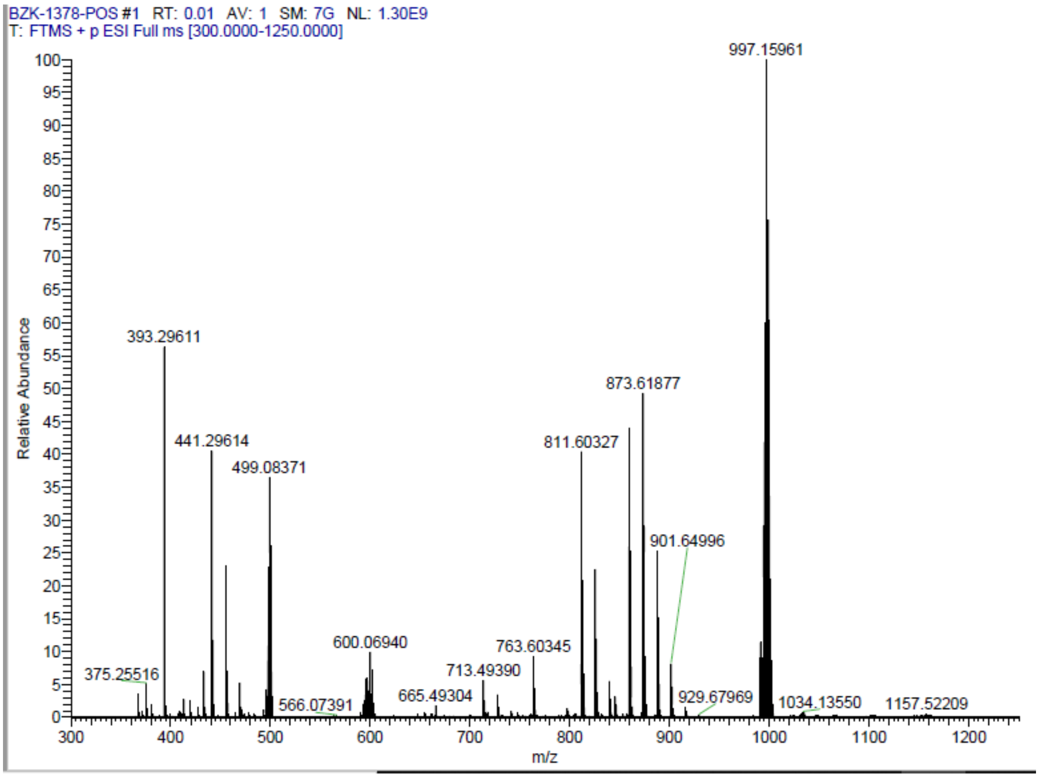

Figure S35. Mass spectrum of **Ru5S**

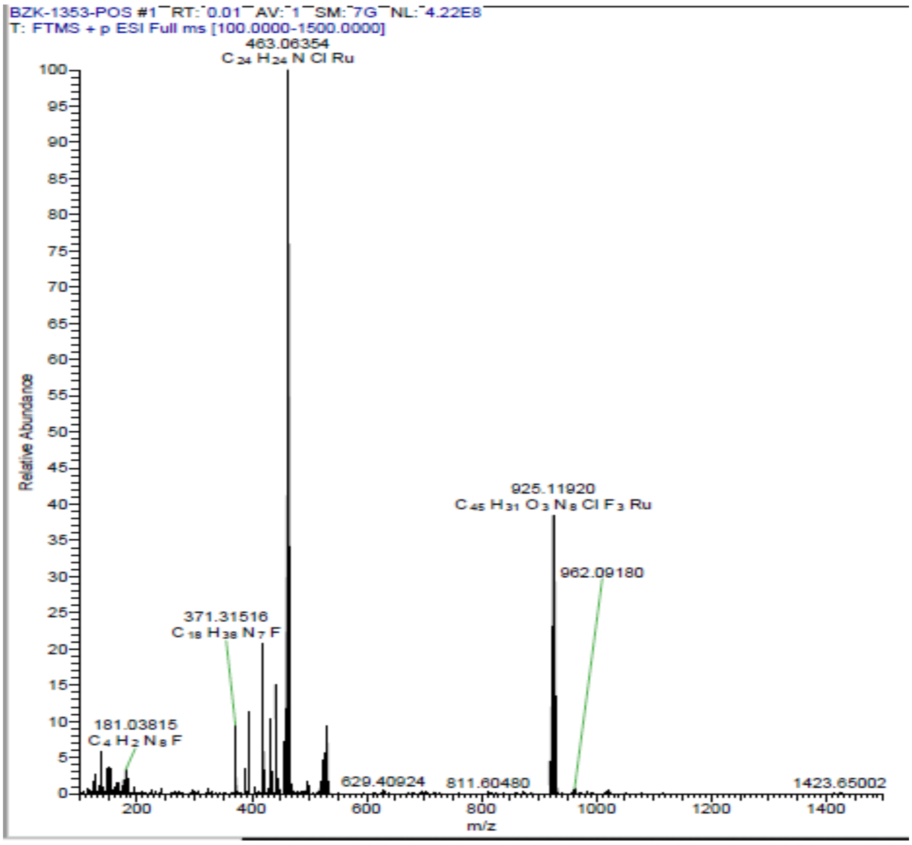

Figure S36. Mass spectrum of **Ru6S**

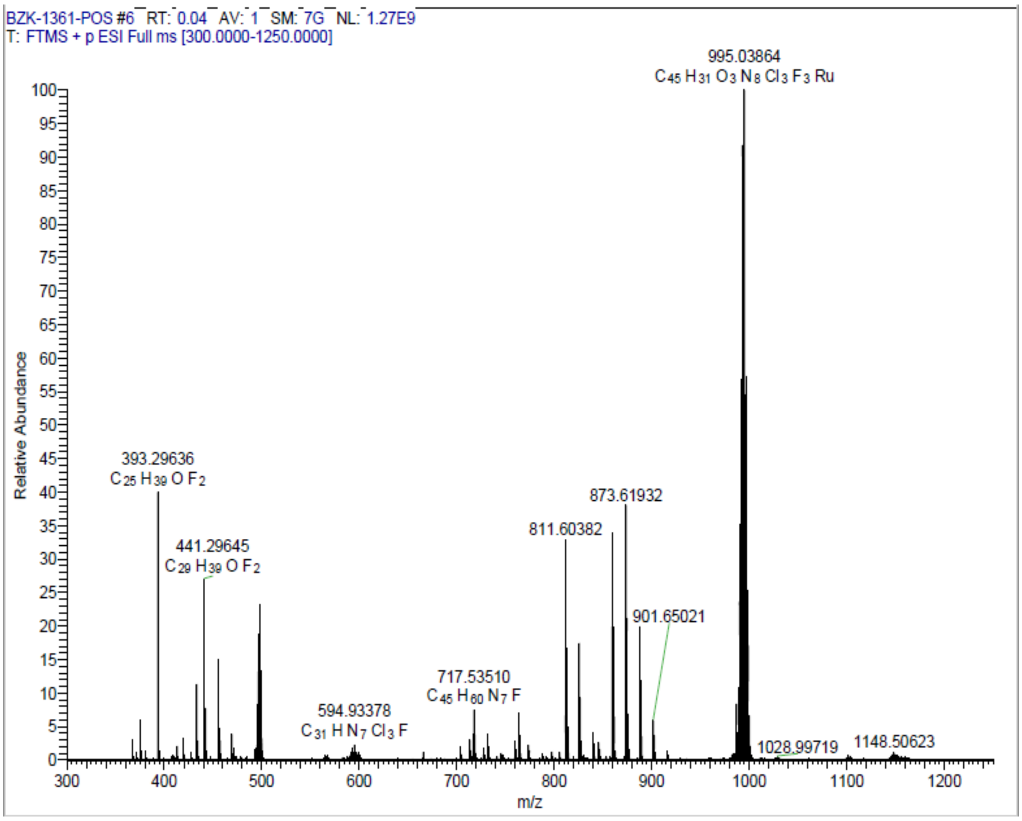

Figure S37. Mass spectrum of **Ru7S**

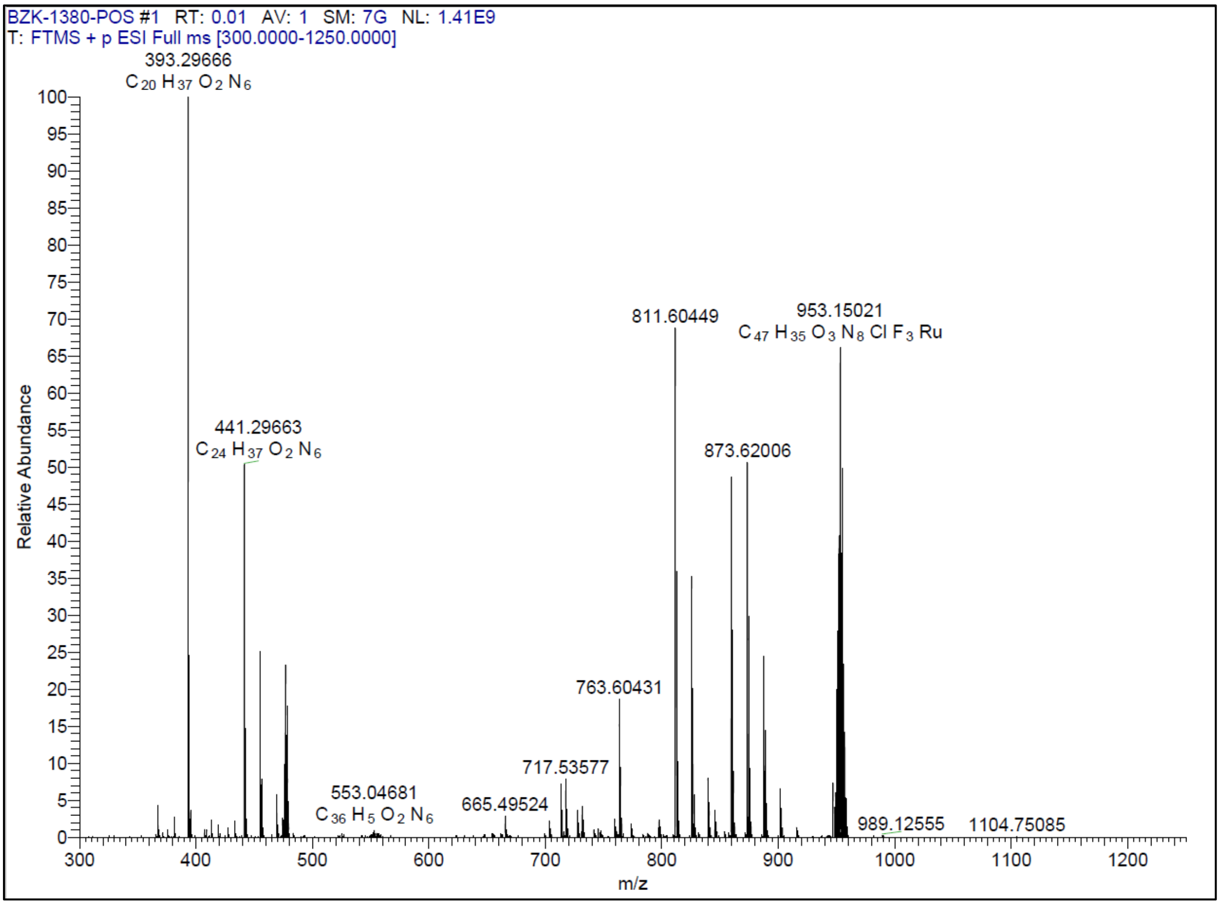

Figure S38. Mass spectrum of **Ru8S**

Peak purity analysis of the compounds

Method: A Shimadzu HPLC system (Kyoto, Japan) with an LC-30AD pump and SPD-20A UV/VIS detector was used for the determination of the compounds. Chromatographic separation was carried out on a Phenomenex C18 column (250×4.6 mm, 5 µm) at 30 °C using methanol:water (90:10, v/v) with 0.1% formic acid isocratically at a flow rate of 1 ml/min. The chromatographic peaks were detected via a UV detector at 290 nm.

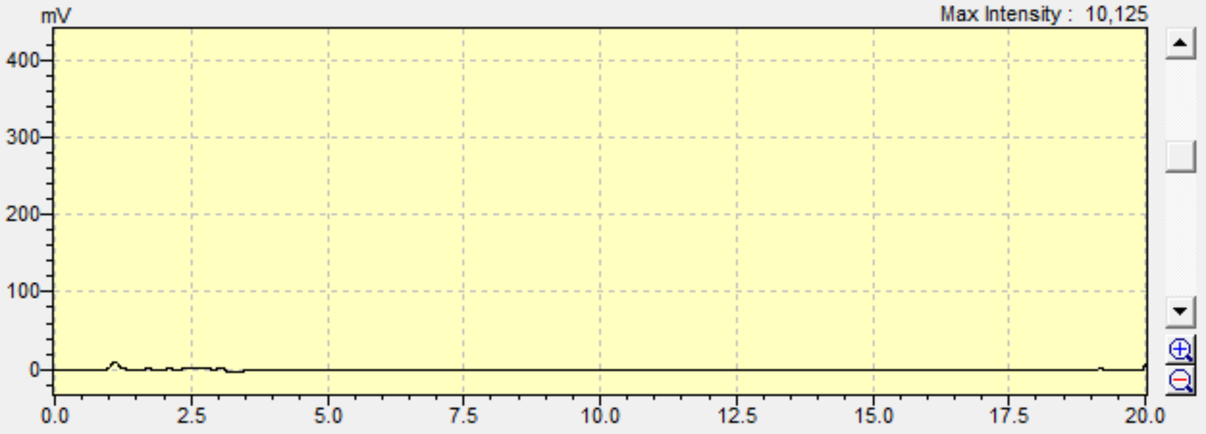

Figure S39. Methanol HPLC trace

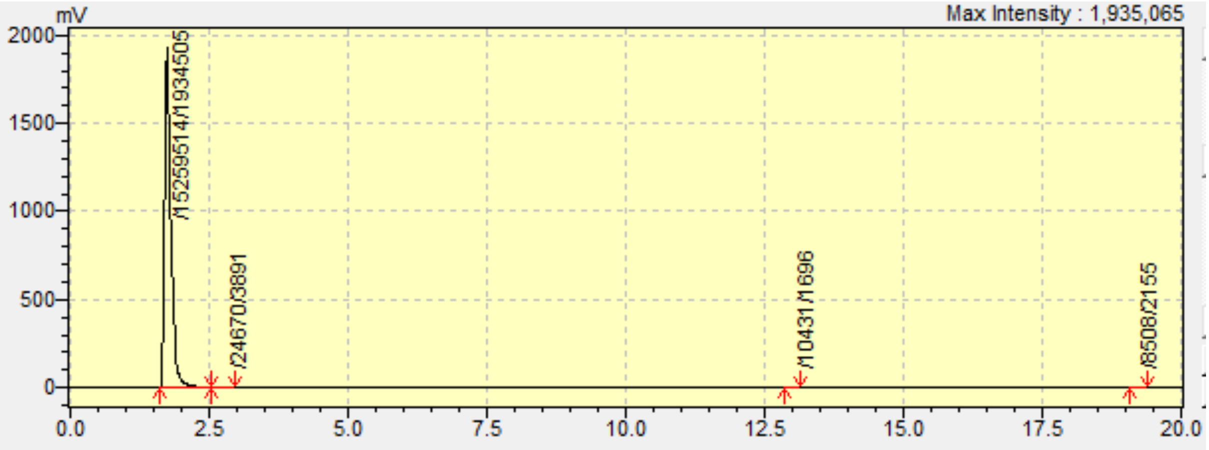

	Select	Ret. Time	Area	Height
1	☐	1.737	15259513.6	1934505.
2	☐	2.769	24670.4	3891.1
3	☐	13.002	10431.5	1696.3
4	☐	19.163	8507.5	2155.3

Figure S40. Compound **Ru1S** HPLC trace**;** Purity=99.72%

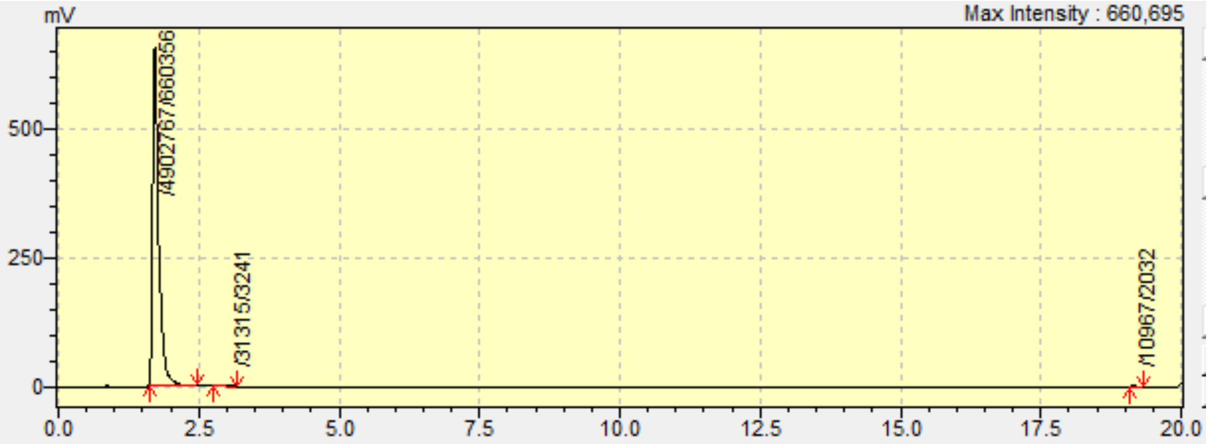

	Select	Ret. Time	Area	Height
1	☐	1.710	4902766.6	660355.8
2	☐	3.031	31314.7	3241.0
3	☐	19.162	10967.2	2032.3

Figure S41. Compound **Ru2S** HPLC trace; Purity=99.14%

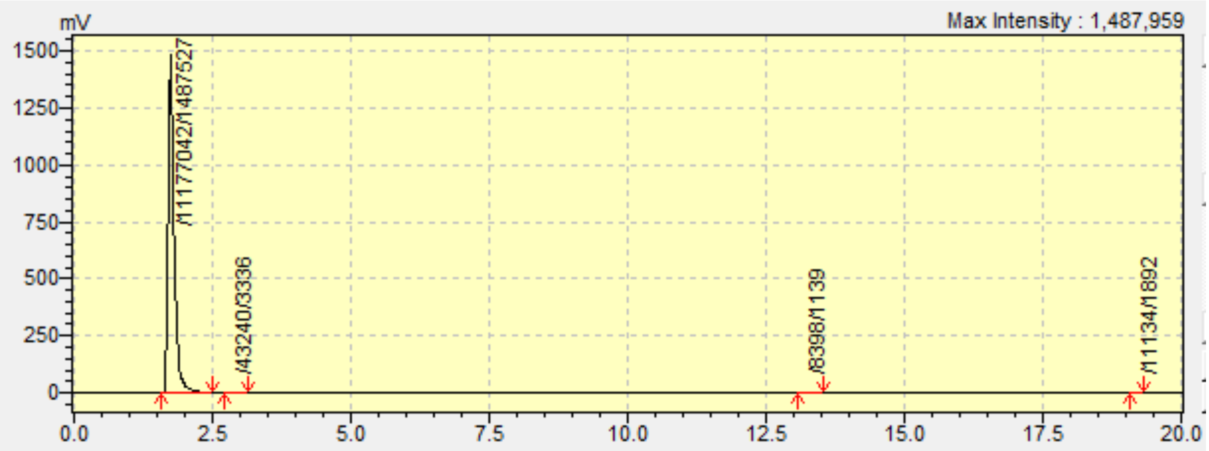

	Select	Ret. Time	Area	Height
1	☐	1.741	11177041.8	1487526.
2	☐	2.817	43240.0	3335.8
3	☐	13.191	8397.8	1139.5
4	☐	19.168	11134.5	1892.1

Figure S42. Compound **Ru3S** HPLC trace; Purity=99.44%

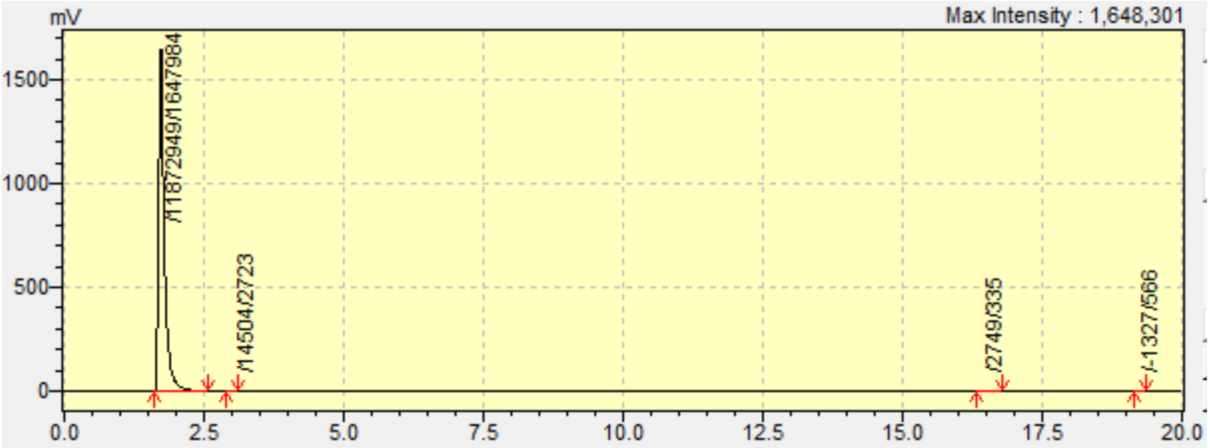

	Select	Ret. Time	Area	Height
1	☐	1.724	11872949.5	1647983.
2	☐	3.000	14503.5	2723.0
3	☐	16.395	2749.1	335.1
4	☐	19.176	-1326.5	566.0

Figure S43. Compound **Ru4S** HPLC trace; Purity=99.84%

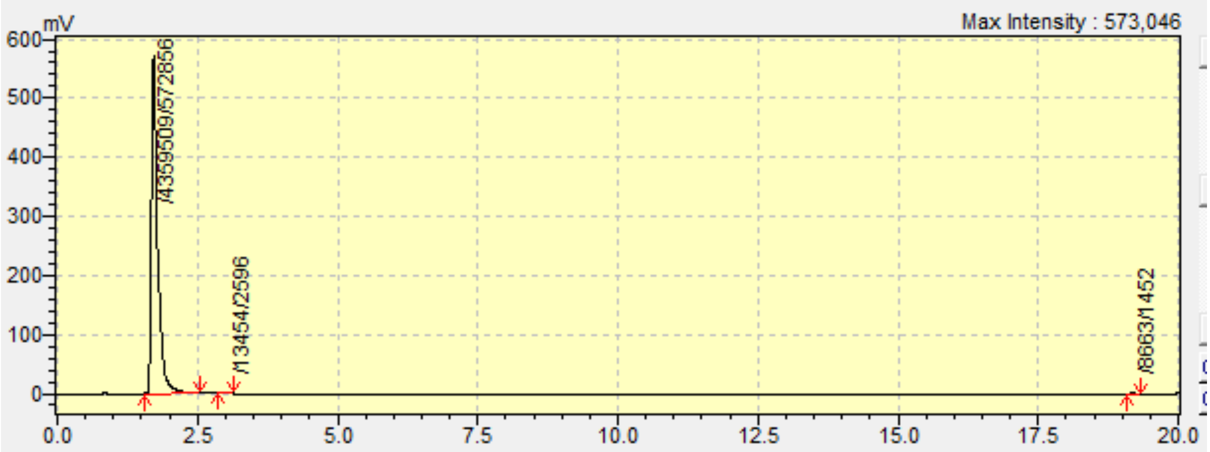

	Select	Ret. Time	Area	Height
1	☐	1.712	4359509.3	572856.3
2	☐	3.021	13453.7	2595.7
3	☐	19.181	8663.4	1452.2

Figure S44. Compound **Ru5S** HPLC trace; Purity=99.49%

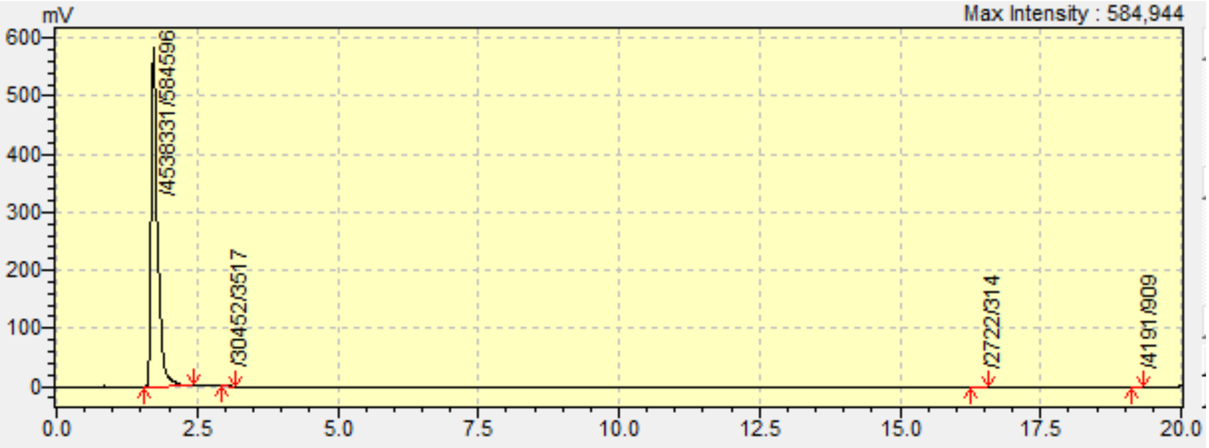

	Select	Ret. Time	Area	Height
1		1.720	4538331.1	584595.9
2		3.015	30451.6	3517.2
3		16.391	2722.4	314.5
4		19.175	4190.6	909.4

Figure S45. Compound **Ru6S** HPLC trace; Purity=99.18%

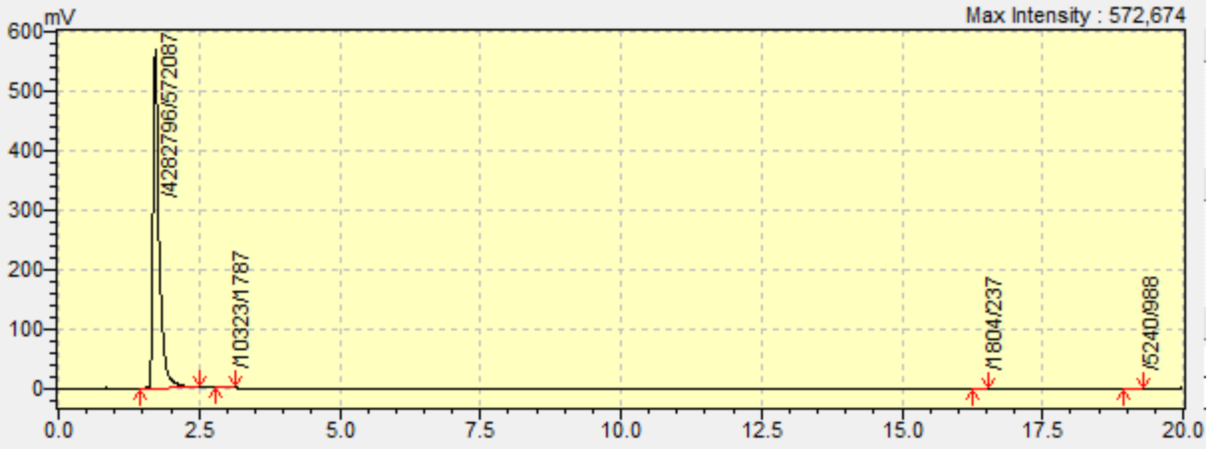

	Select	Ret. Time	Area	Height
1		1.717	4282795.9	572087.4
2		2.993	10323.1	1787.0
3		16.396	1804.2	237.4
4		19.179	5240.2	987.6

Figure S46. Compound **Ru7S** HPLC trace; Purity=99.60%

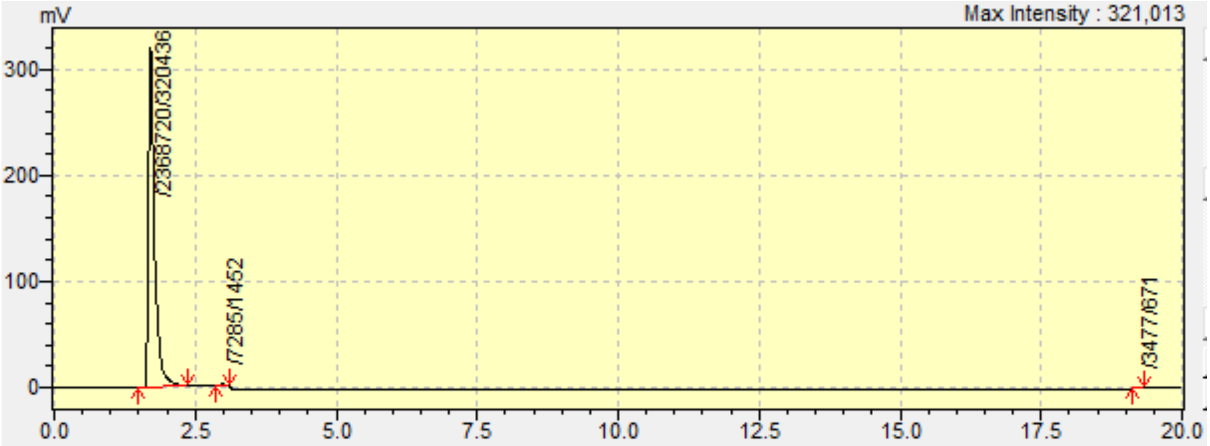

	Select	Ret. Time	Area	Height
1	☐	1.705	2368720.5	320436.1
2	☐	2.984	7285.3	1452.1
3	☐	19.184	3476.6	670.7

Figure S47. Compound **Ru8S** HPLC trace**;** Purity=99.55%

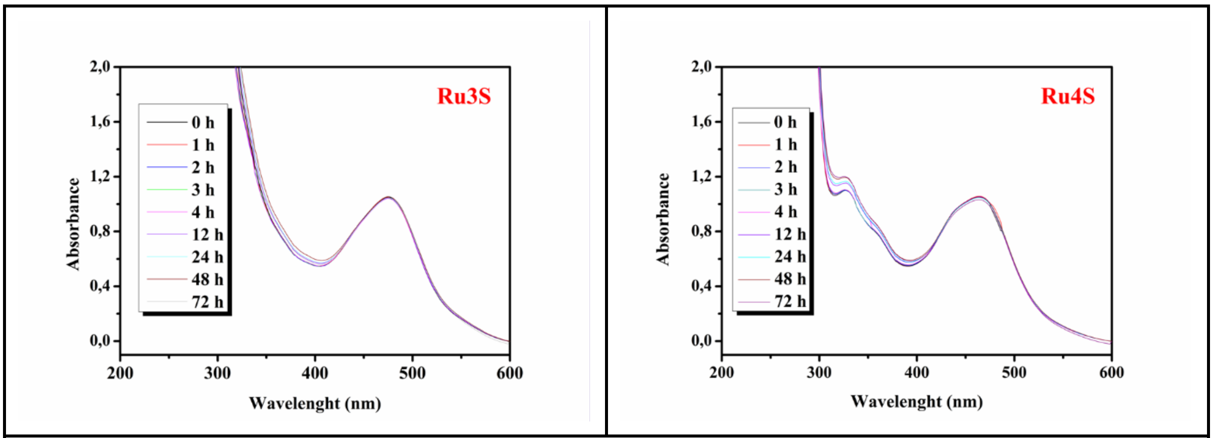

Figure S48. UV-vis stability analysis of **Ru3S** and **Ru4S** in DMSO/water (1-99% v/v)

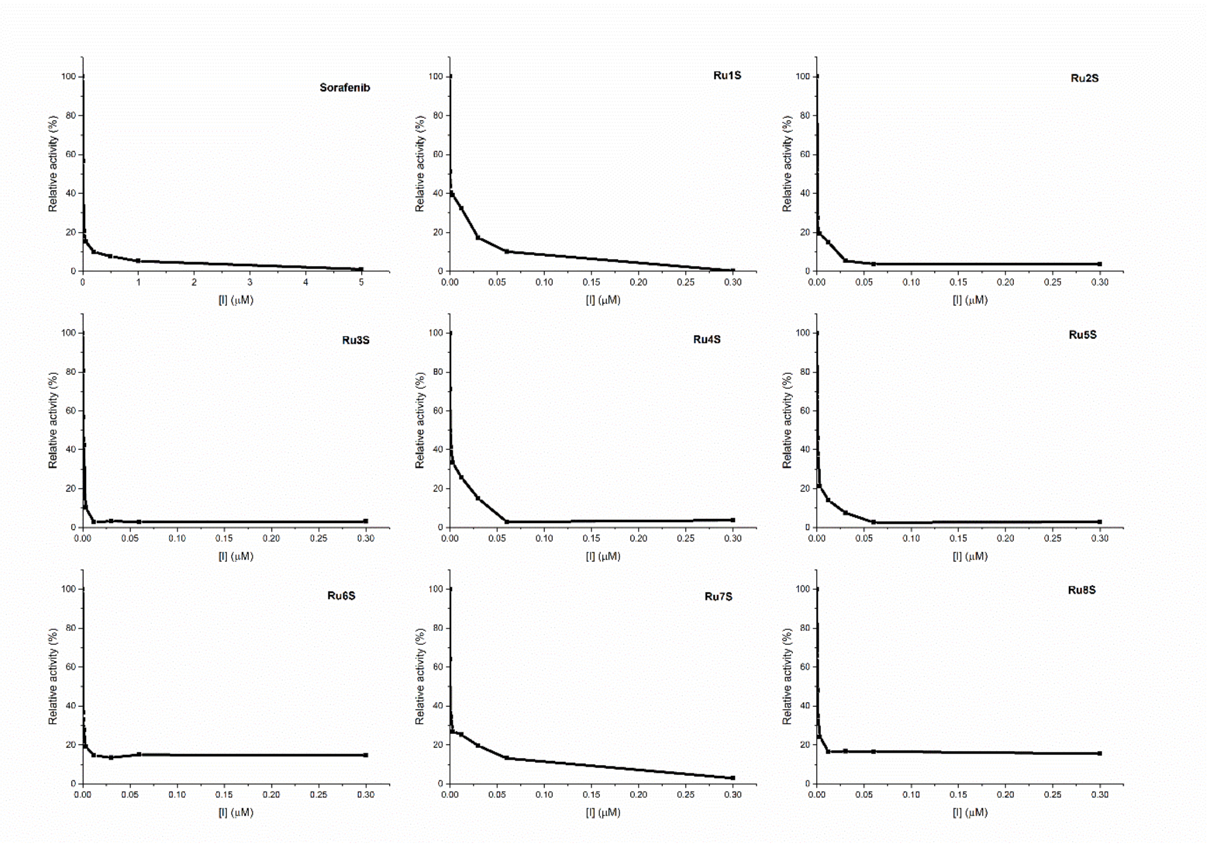

Figure S49. Enzyme inhibition graphics

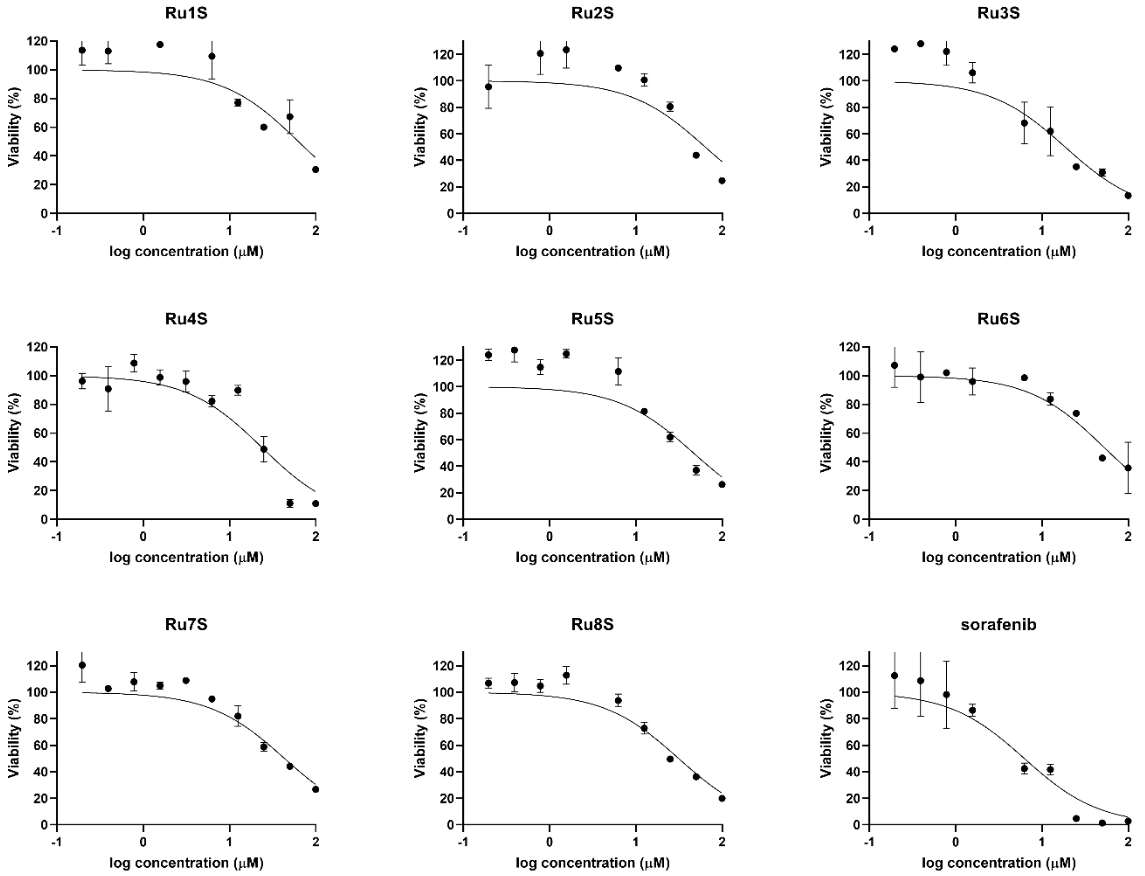

Figure S50. Concentration-%viability plot of complexes in HepG2 cell line

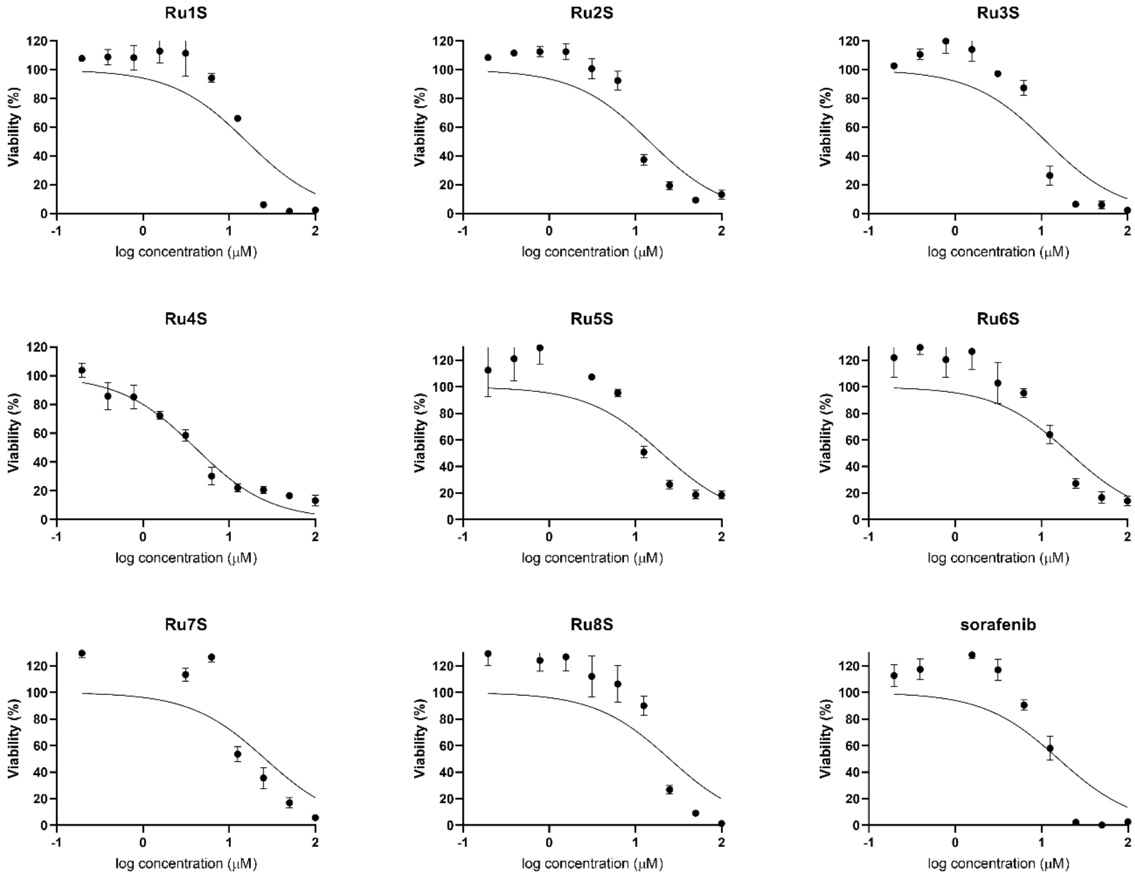

Figure S51. Concentration-%viability plot of complexes in Caco2 cell line

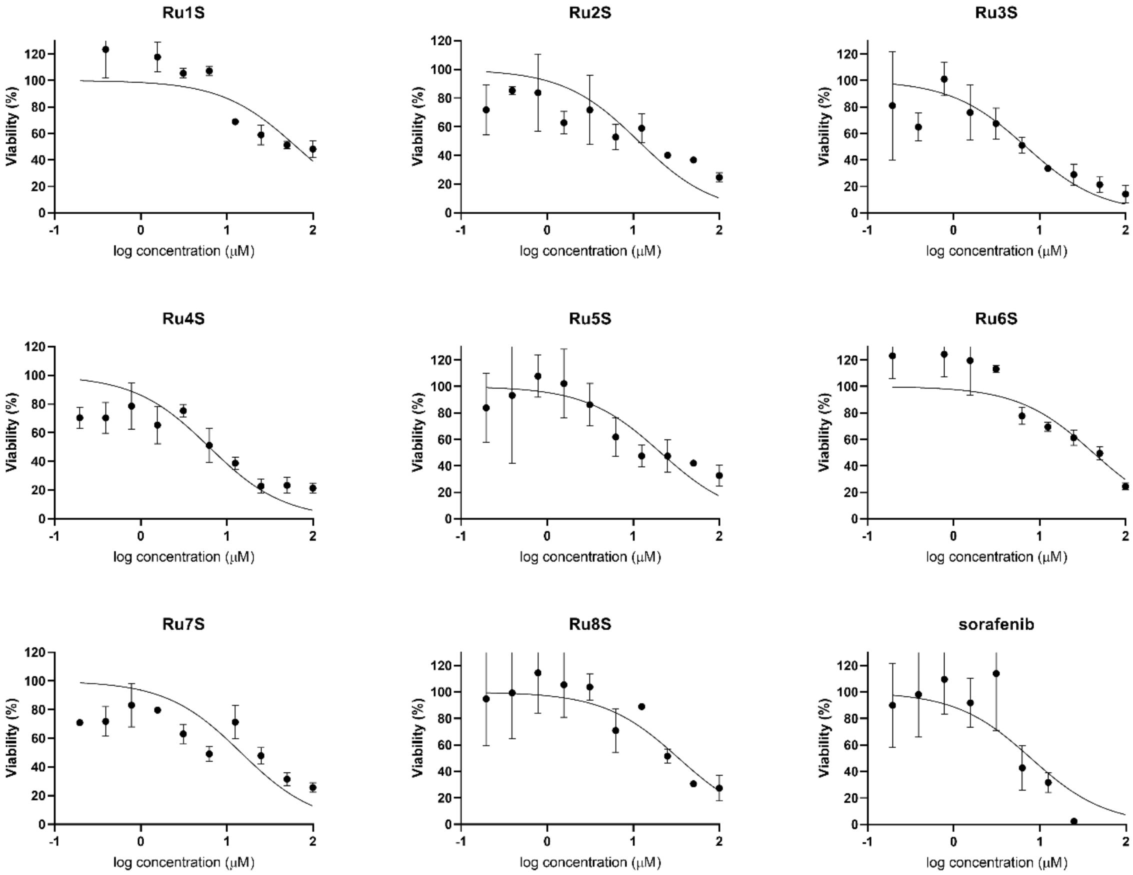

Figure S52. Concentration-%viability plot of complexes in HT-29 cell line

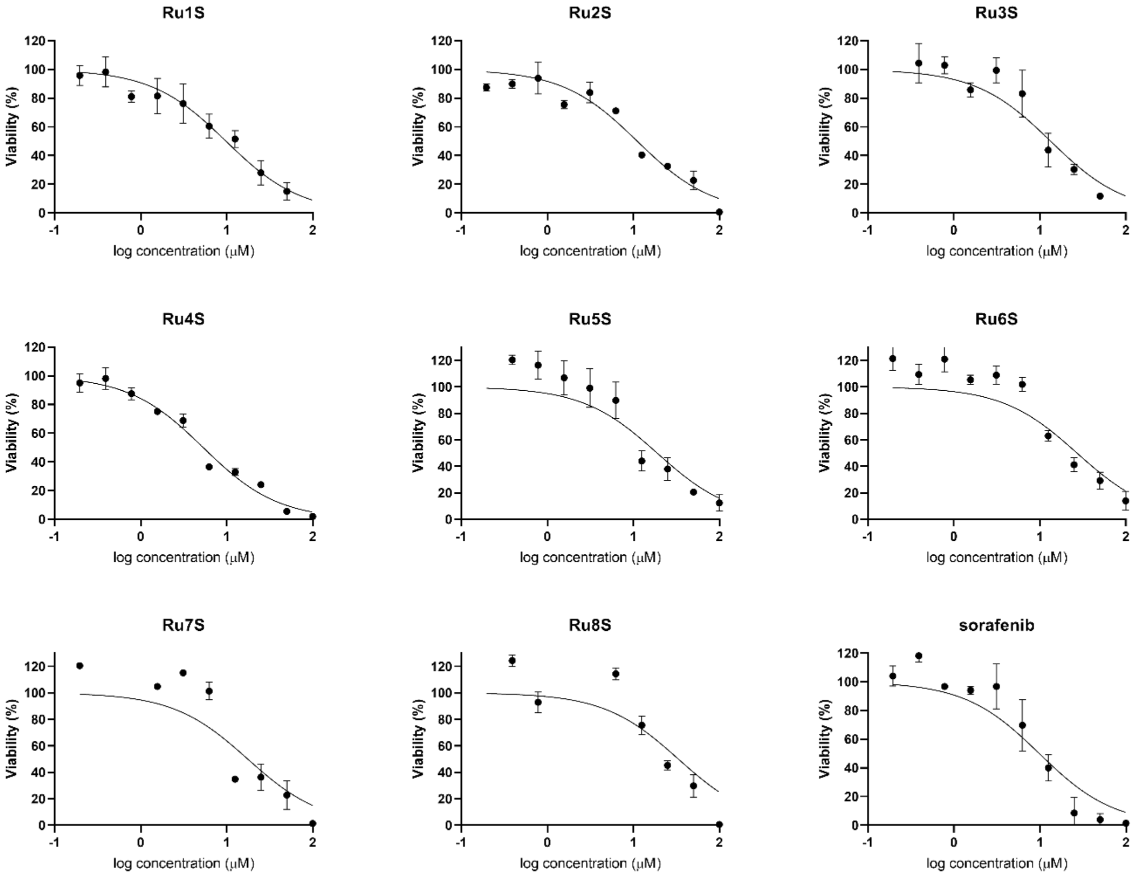

Figure S53. Concentration-%viability plot of complexes in MCF-7 cell line

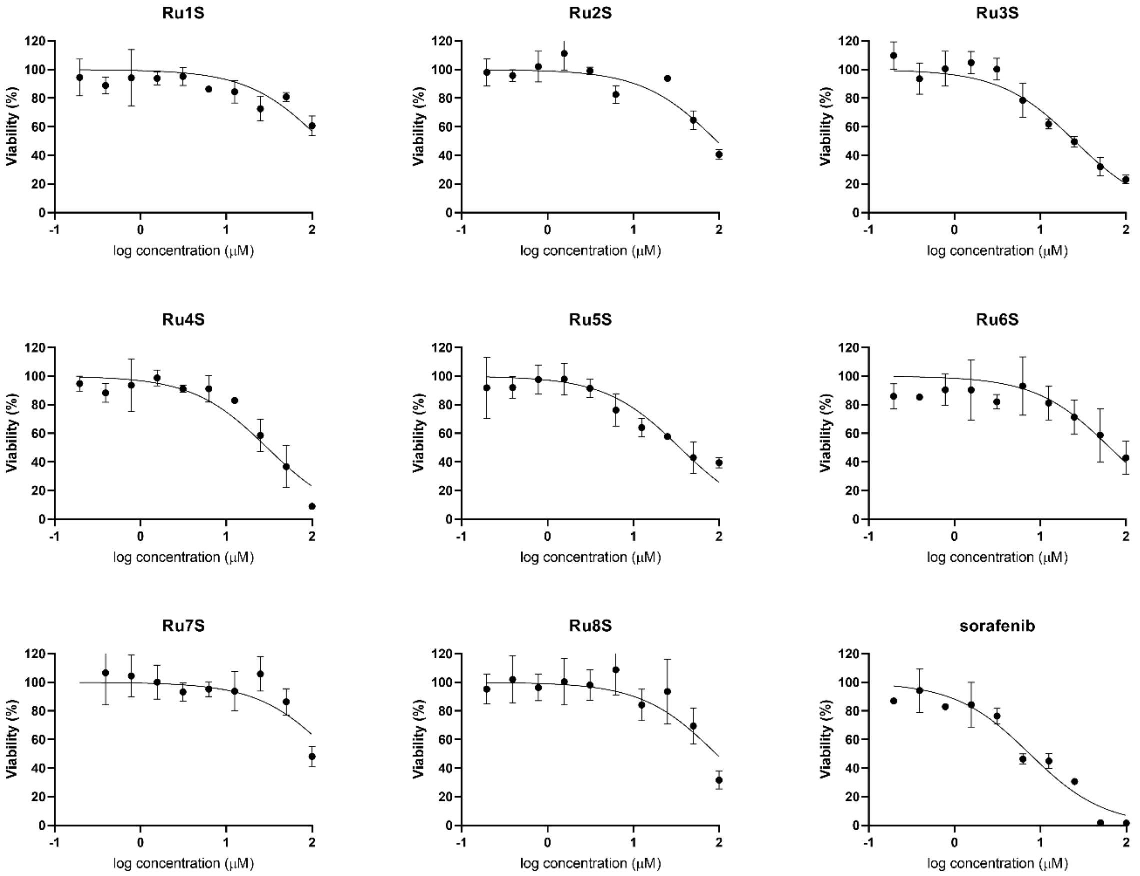

Figure S54. Concentration-%viability plot of complexes in A549 cell line

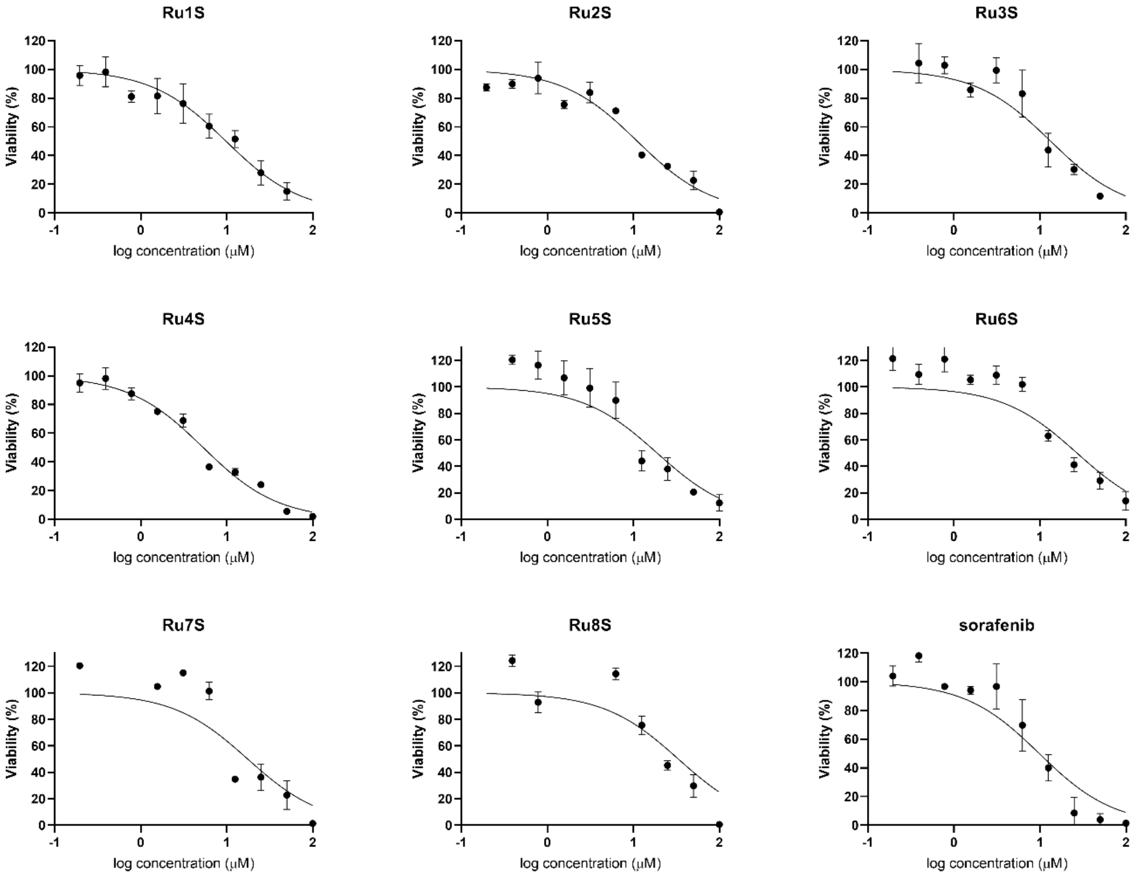

Figure S55. Concentration-%viability plot of complexes in HEK293T cell line

Control 1

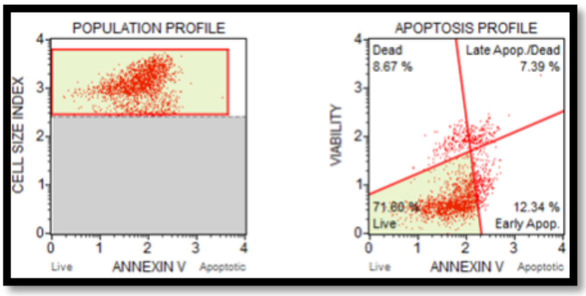

Control 2

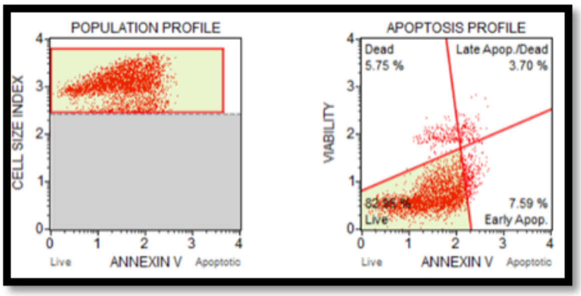

Ru3S_3.12µM_1

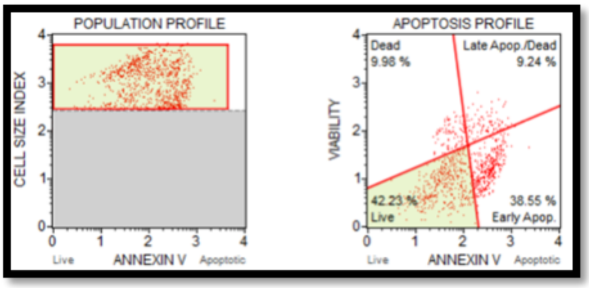

Ru3S_3.12µM_2

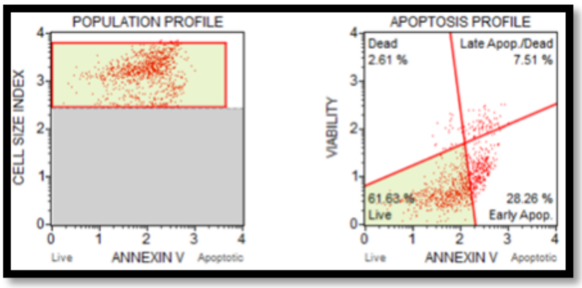

Ru3S_12.5µM_1

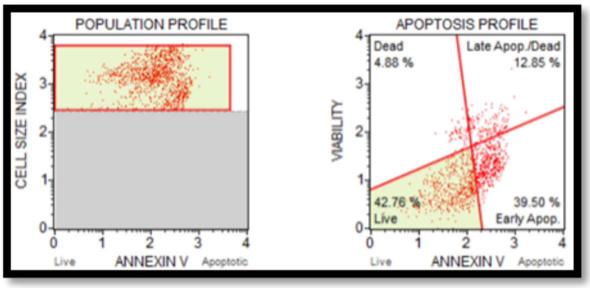

Ru3S _12.5µM_2

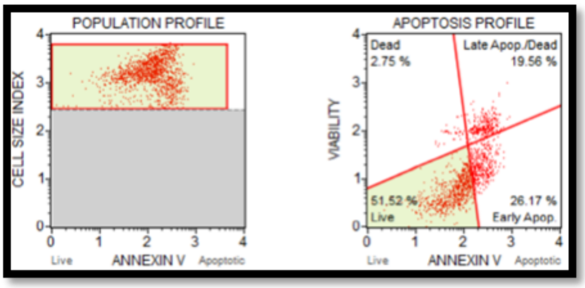

Ru3S_50µM_1

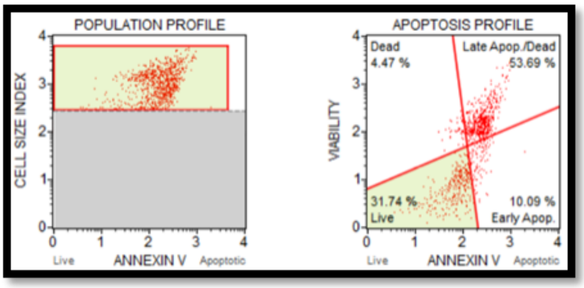

Ru3S_50µM_2

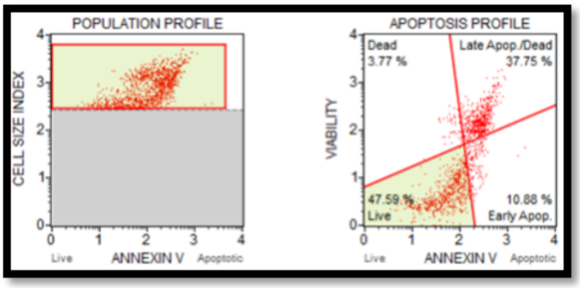

Figure S56. Apoptosis profiles of **Ru3S**, **Ru4S**, Sorafenib, **M1** and **M2**

Ru4S_0.78µM_1

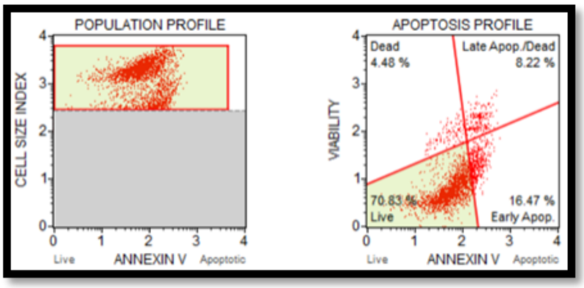

Ru4S _0.78µM_2

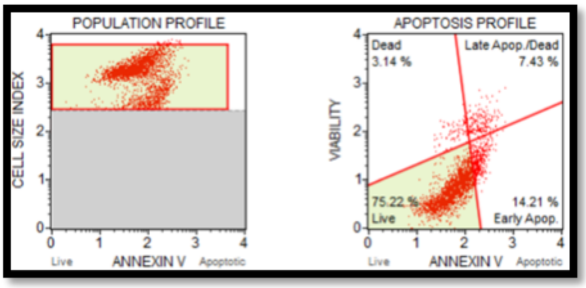

Ru4S_3.12µM_1

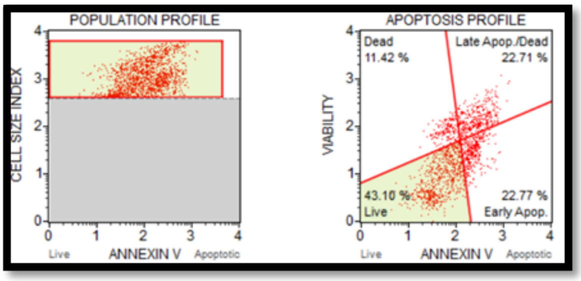

Ru4S_3.12µM_2

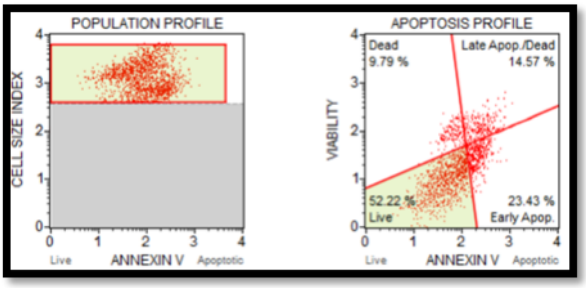

Ru4S_12.5µM_1

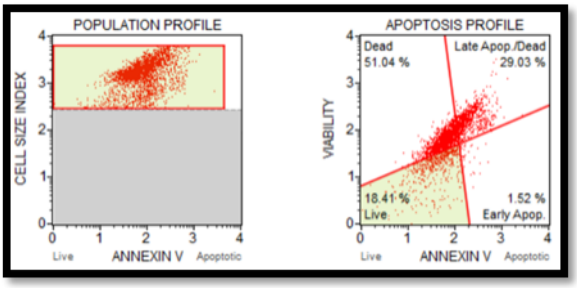

Ru4S_12.5µM_2

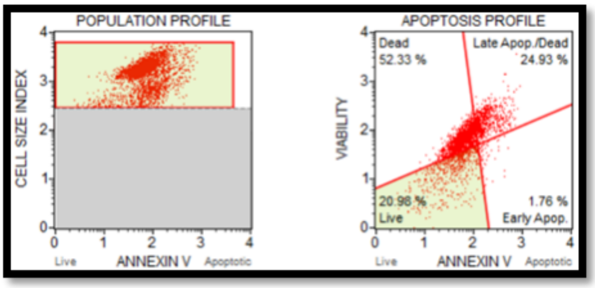

Ru4S_50µM_1

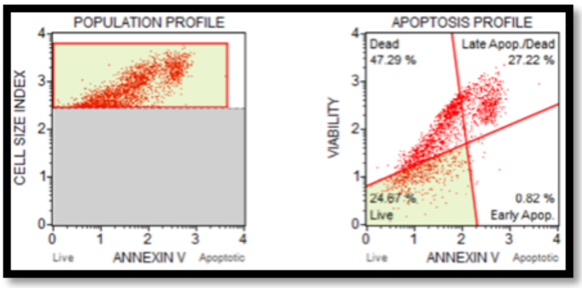

Ru4S_50µM_2

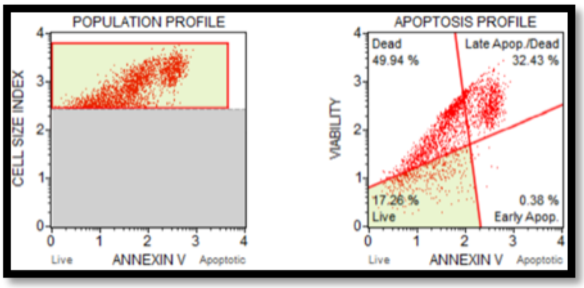

Figure S56. Apoptosis profiles of **Ru3S**, **Ru4S**, Sorafenib, **M1** and **M2** continue

Sorafenib_3.12µM_1

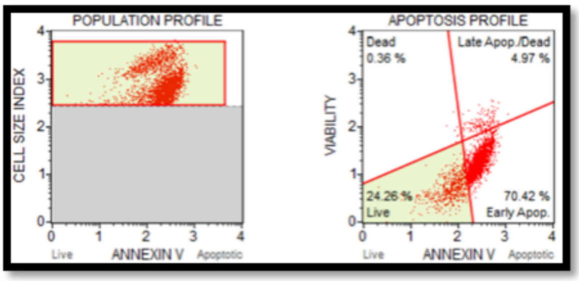

Sorafenib _3.12µM_2

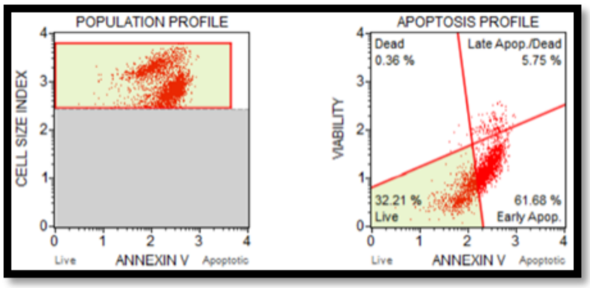

Sorafenib _12.5µM_1

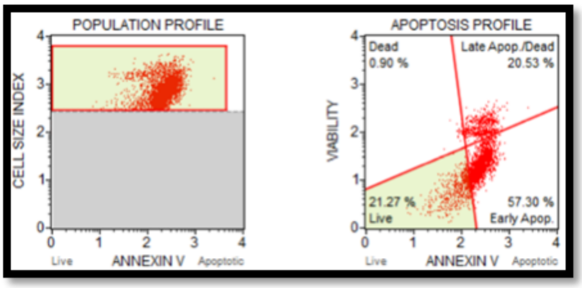

Sorafenib _12.5µM_2

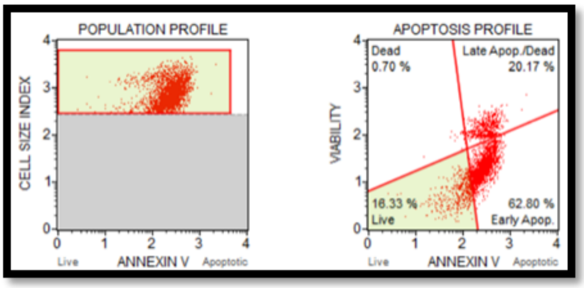

Sorafenib _50µM_1

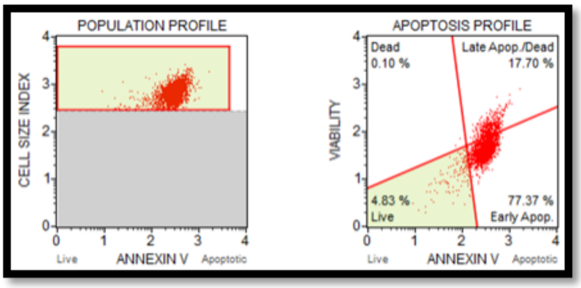

Sorafenib_50µM_2

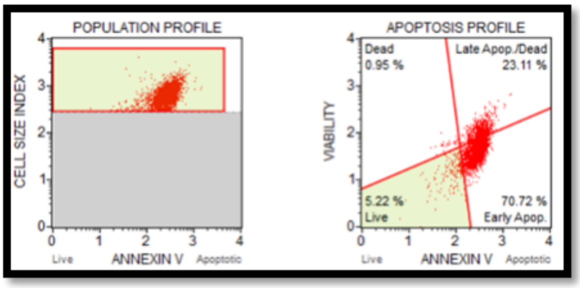

Figure S56. Apoptosis profiles of **Ru3S**, **Ru4S**, Sorafenib, **M1** and **M2** continue

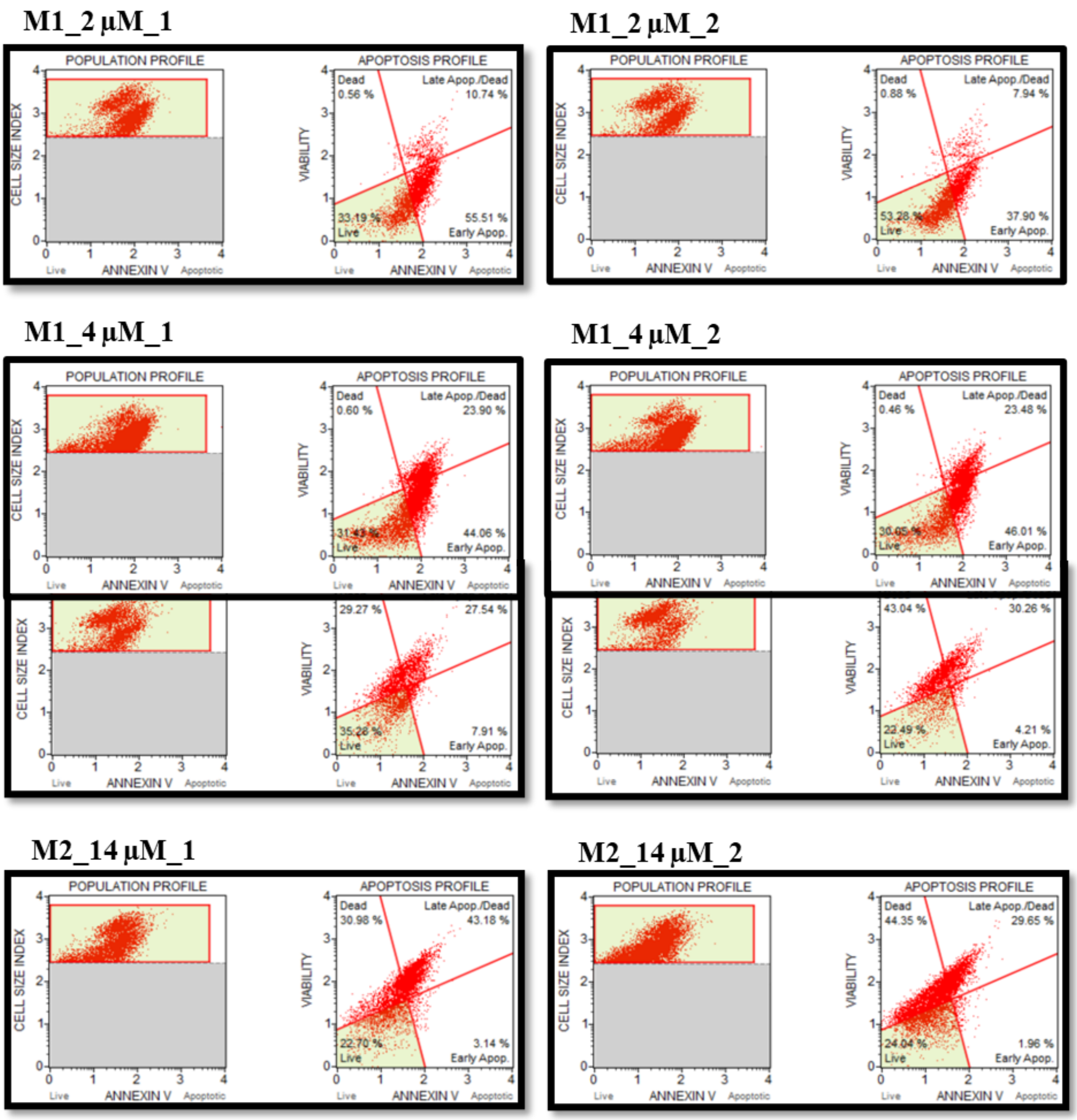

Figure S56. Apoptosis profiles of **Ru3S**, **Ru4S**, Sorafenib, **M1** and **M2** continue

Control 1

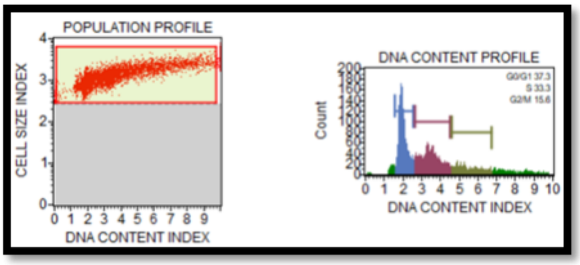

Control 2

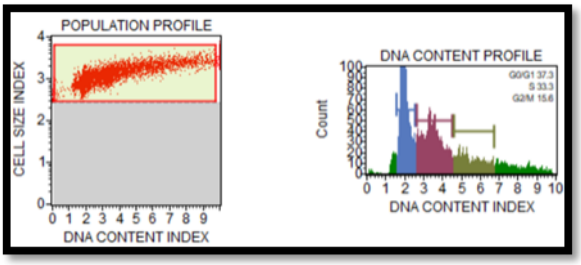

Ru3S_3.12µM_1

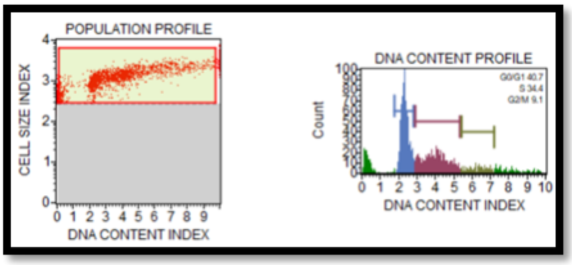

Ru3S_3.12µM_2

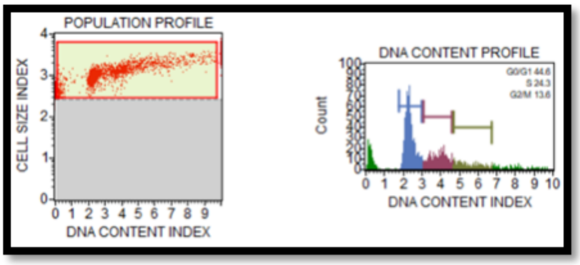

Ru3S_12.5µM_1

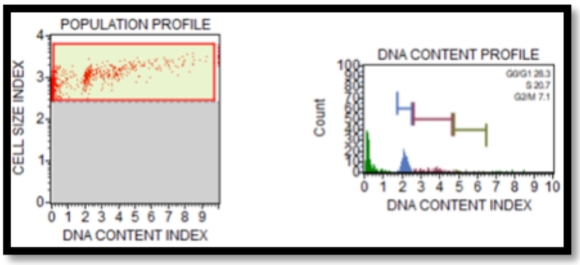

Ru3S _12.5µM_2

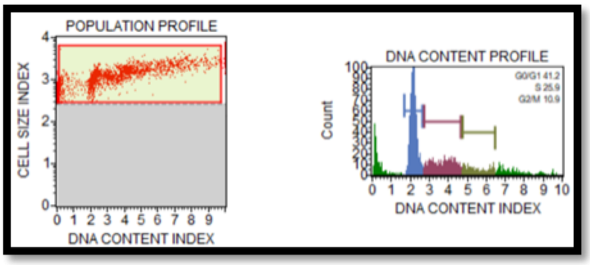

Ru3S_50µM_1

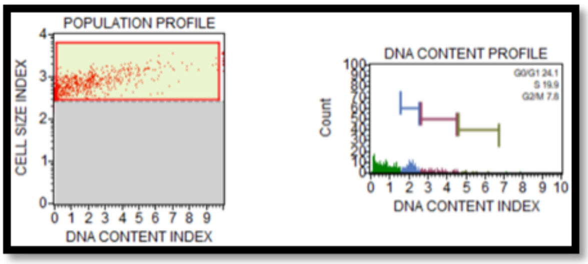

Ru3S_50µM_2

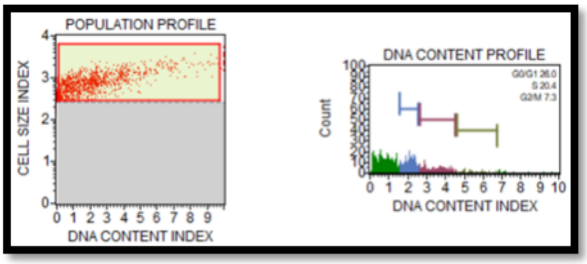

Figure S57. Cell cycle profiles of **Ru3S**, **Ru4S** and Sorafenib

Ru4S_0.78µM_1

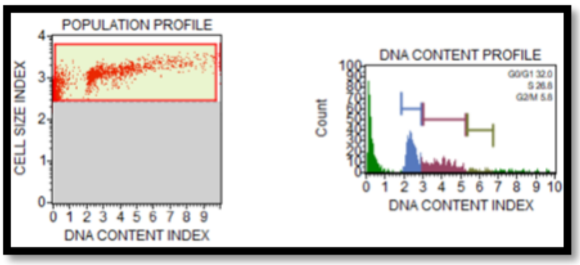

Ru4S _0.78µM_2

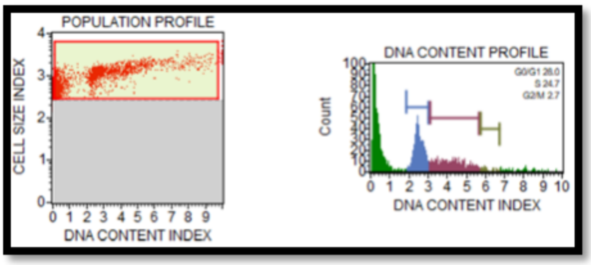

Ru4S_3.12µM_1

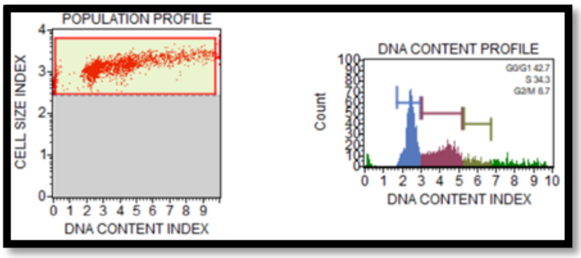

Ru4S_3.12µM_2

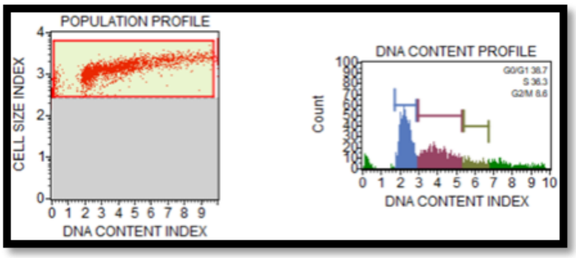

Ru4S_12.5µM_1

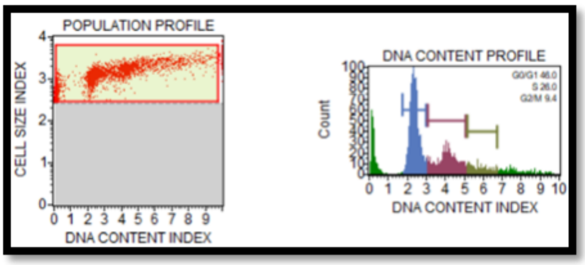

Ru4S_12.5µM_2

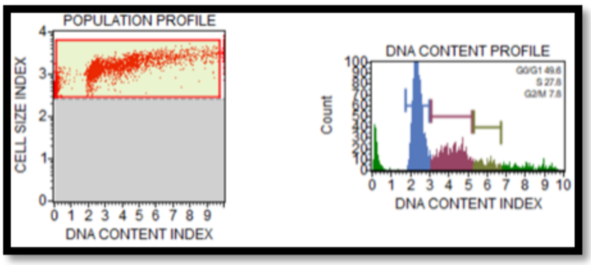

Ru4S_50µM_1

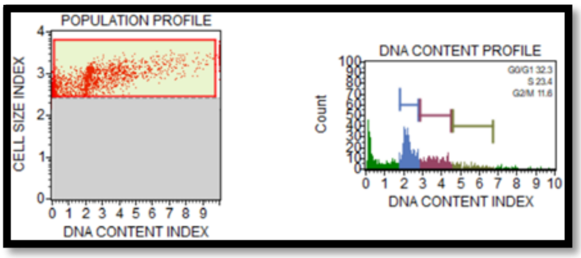

Ru4S_50µM_2

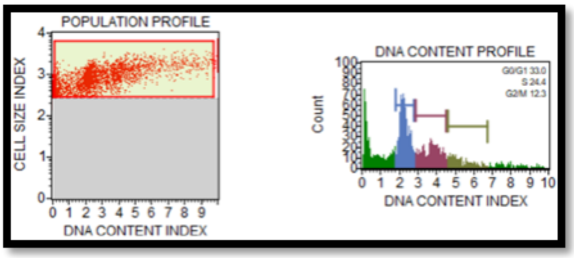

Figure S57. Cell cycle profiles of **Ru3S**, **Ru4S** and Sorafenib continue

Sorafenib_3.12µM_1

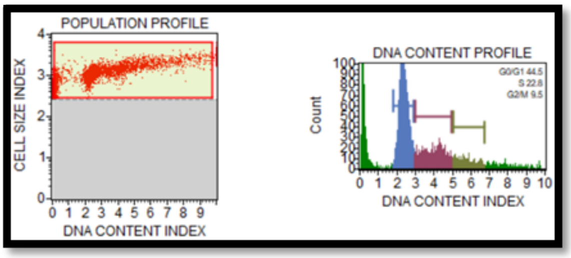

Sorafenib _3.12µM_2

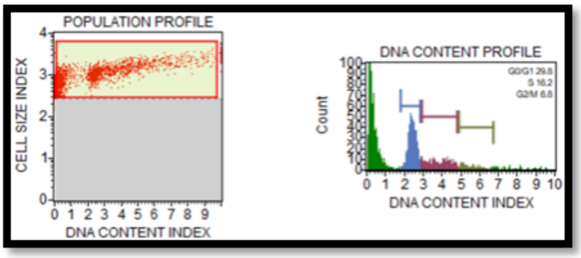

Sorafenib _12.5µM_1

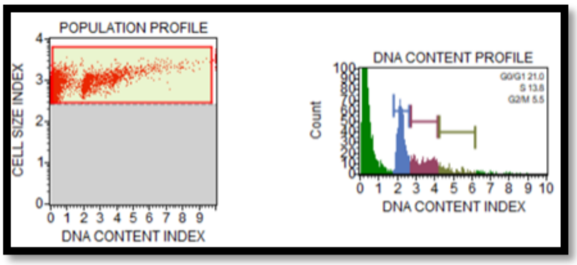

Sorafenib _12.5µM_2

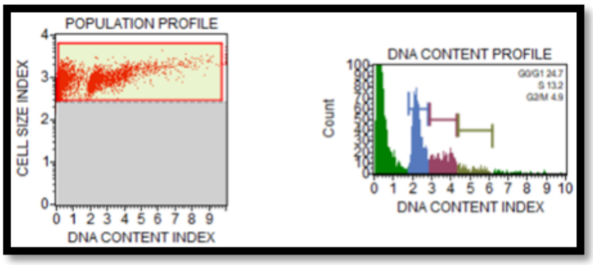

Sorafenib _50µM_1

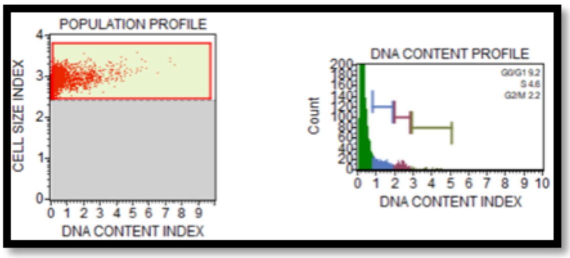

Sorafenib_50µM_2

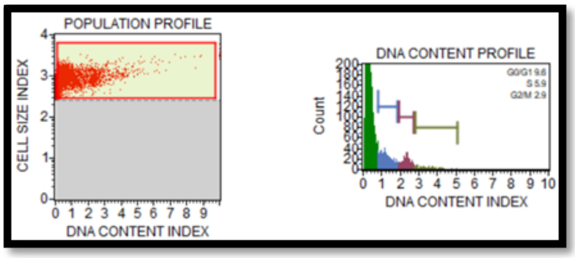

Figure S57. Cell cycle profiles of **Ru3S**, **Ru4S** and Sorafenib continue

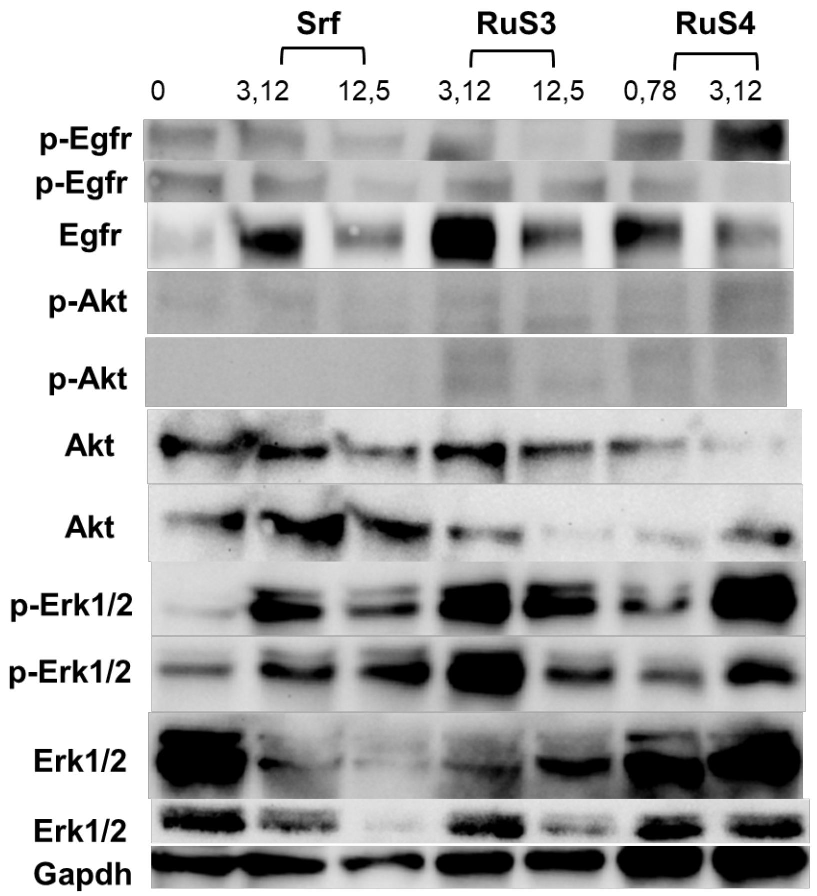

Figure S58: Repetitions of western blot experiment in HepG2 cell line

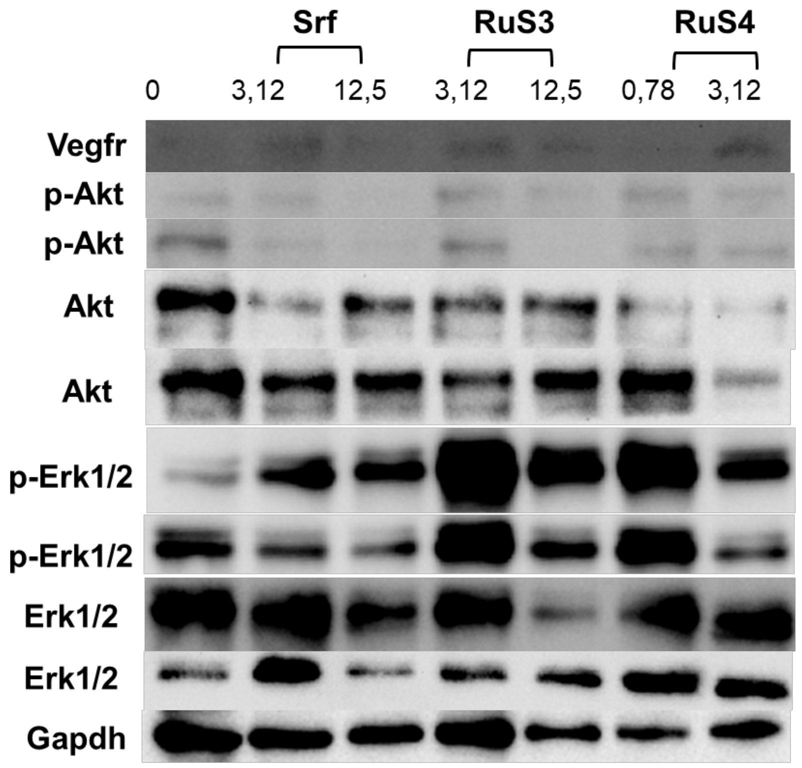

Figure S59: Repetitions of western blot experiment in HUVEC cell line

$$DLC\ (\text{wt\%}) = \frac{\textit{weight of drug in micelles} \times 100}{\text{weight of drug-loaded micelles}}$$

$$\%EE\ (\text{wt\%}) = \frac{\text{weight of drug in micelles} \times 100}{\text{weight of drug added initially}}$$

Figure S60. Entrapment efficiency (EE) equations

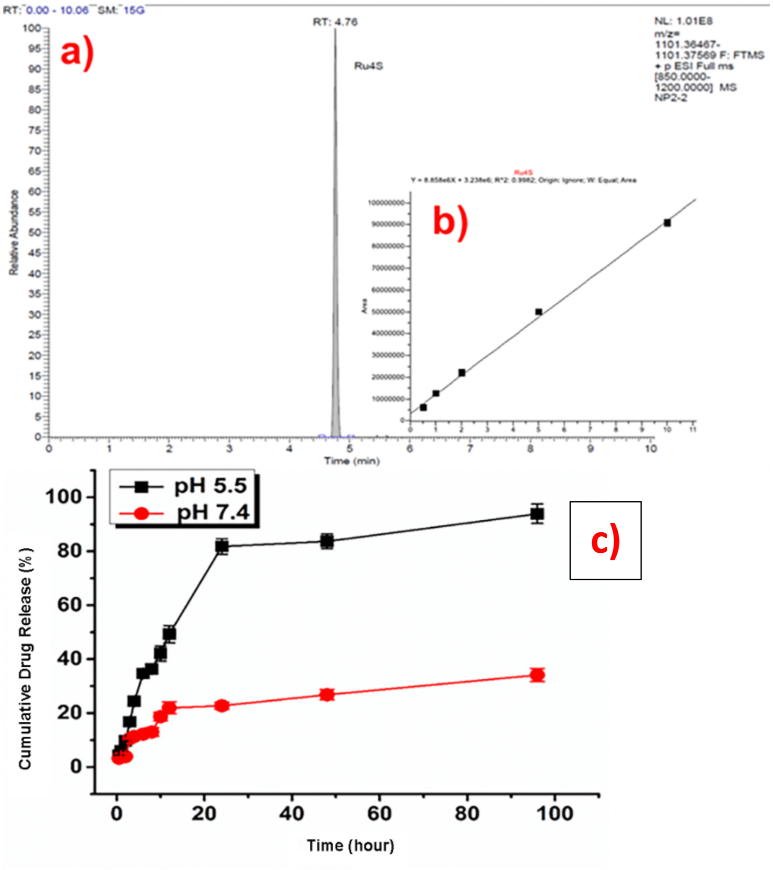

Figure S61. a) LC-HRMS chromatogram of **M2 b)** Calibration plot generated for **Ru4S c)** Drug release profile of **M2 d)** DLS result of **M1** and **M2** micelles

d)

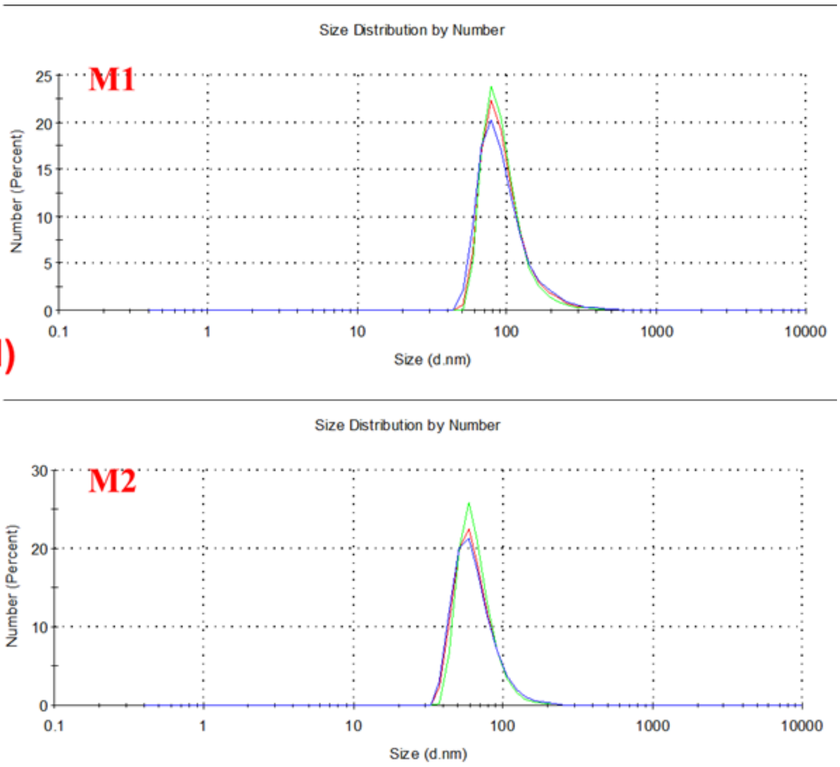

Figure S61. a) LC-HRMS chromatogram of **M2 b)** Calibration plot generated for **Ru4S c)** Drug release profile of **M2 d)** DLS result of **M1** and **M2** micelles

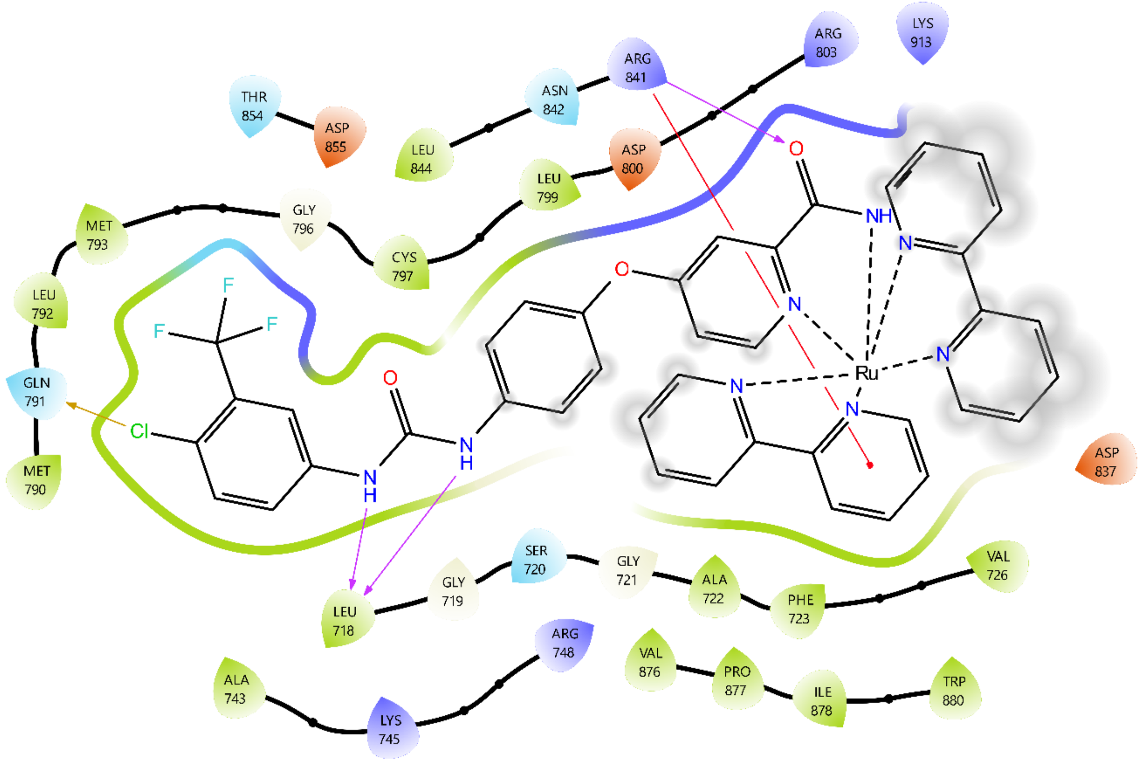

Figure S62. Molecular docking 2D ligand protein interactions between **Ru1S** and the active site of EGFR (PDB: 5X2A)

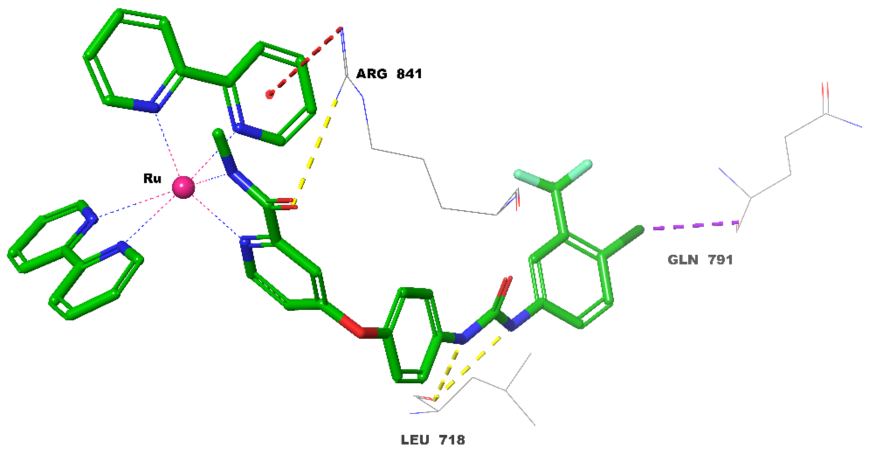

Figure S63. Molecular docking 3D ligand protein interactions between **Ru1S** and the active site of EGFR (PDB: 5X2A)

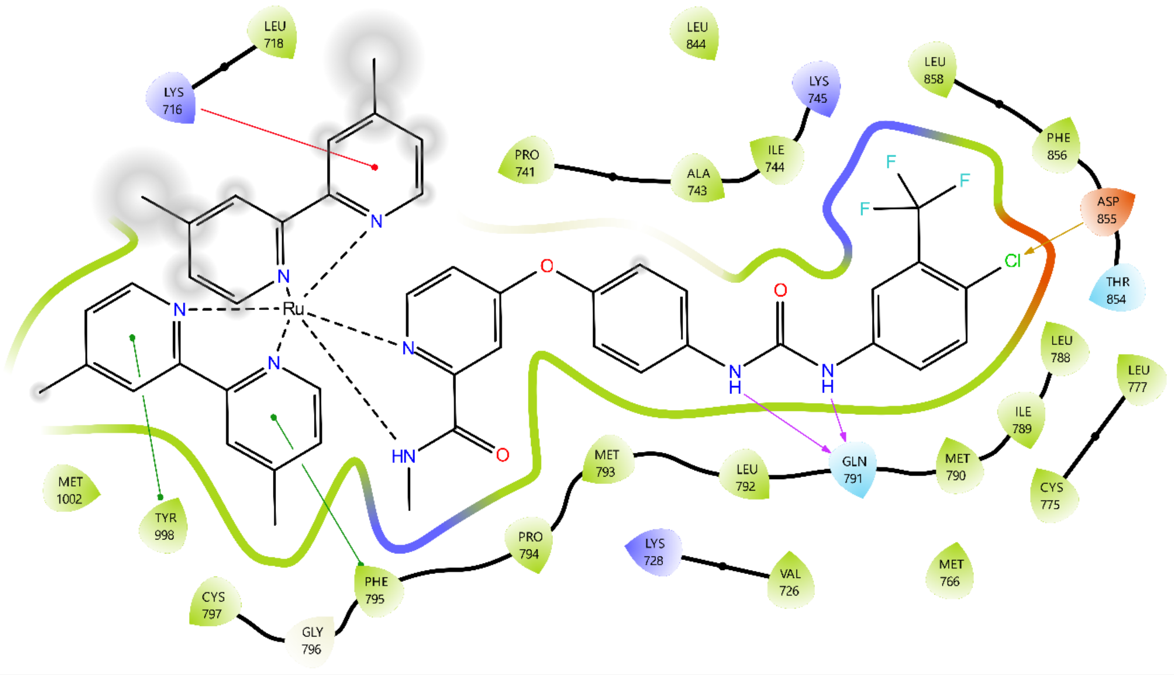

Figure S64. Molecular docking 2D ligand protein interactions between **Ru2S** and the active site of EGFR (PDB: 5X2A)

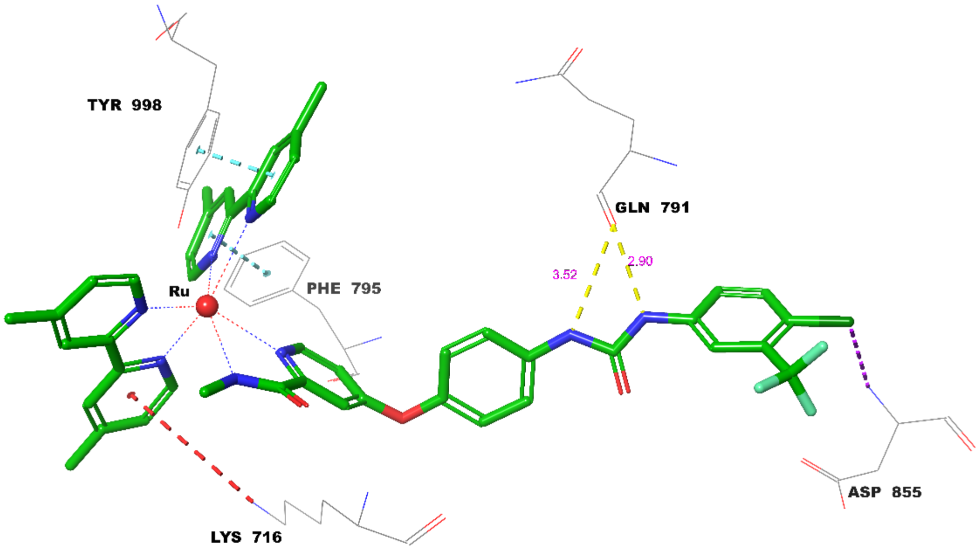

Figure S65. Molecular docking 3D ligand protein interactions between **Ru2S** and the active site of EGFR (PDB: 5X2A)

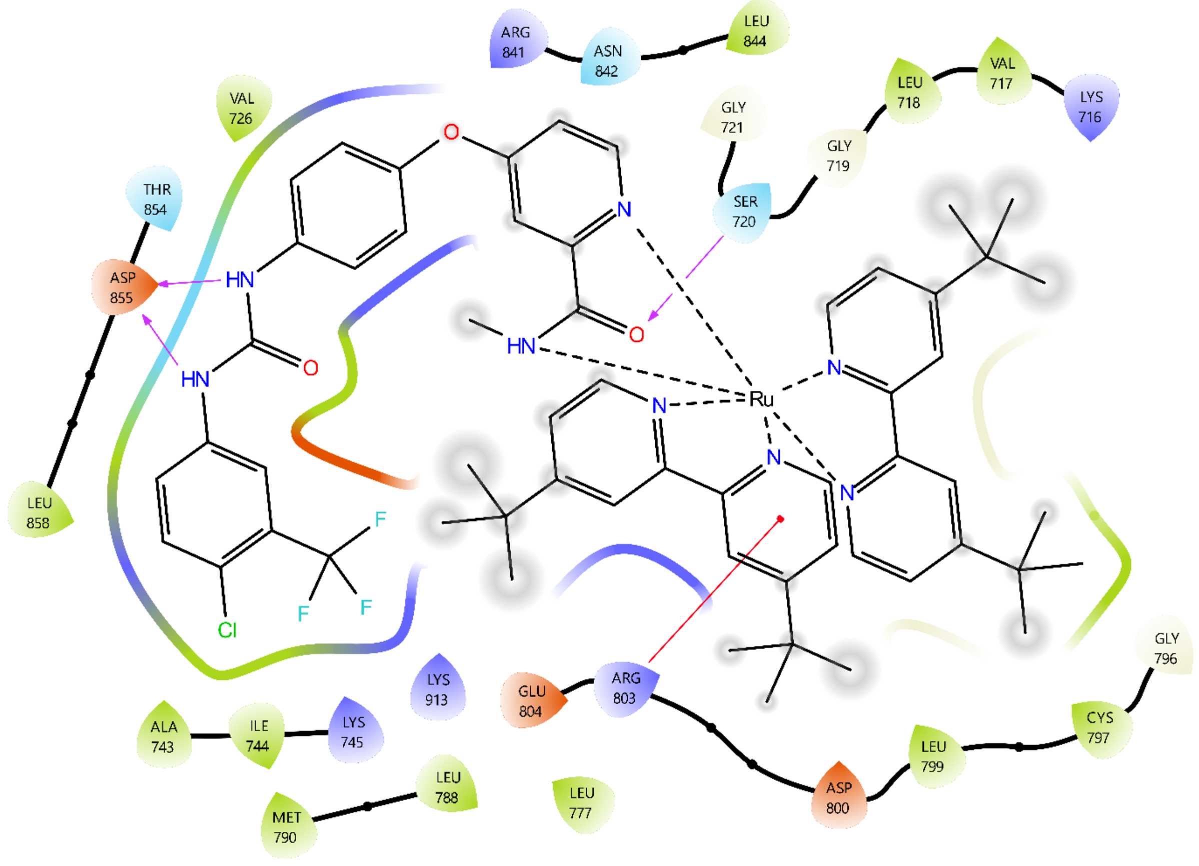

Figure S66. Molecular docking 2D ligand protein interactions between **Ru4S** and the active site of EGFR (PDB: 5X2A)

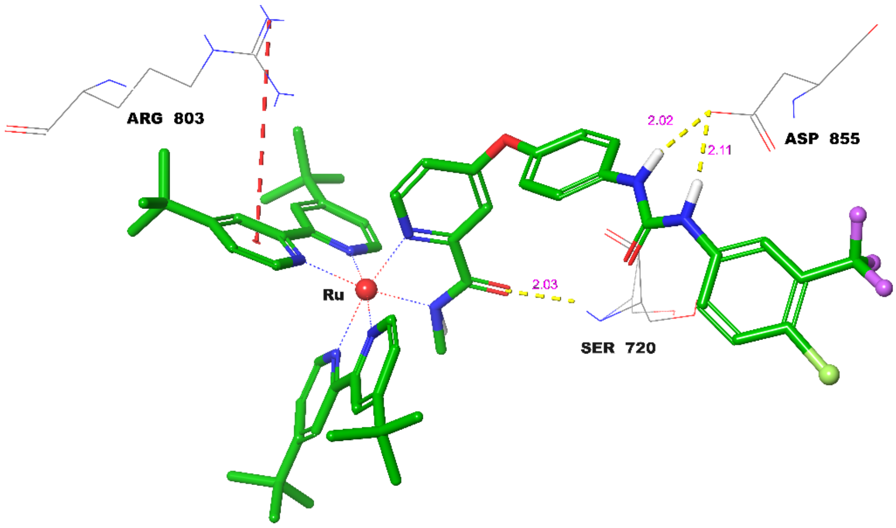

Figure S67. Molecular docking 3D ligand protein interactions between **Ru4S** and the active site of EGFR (PDB: 5X2A)

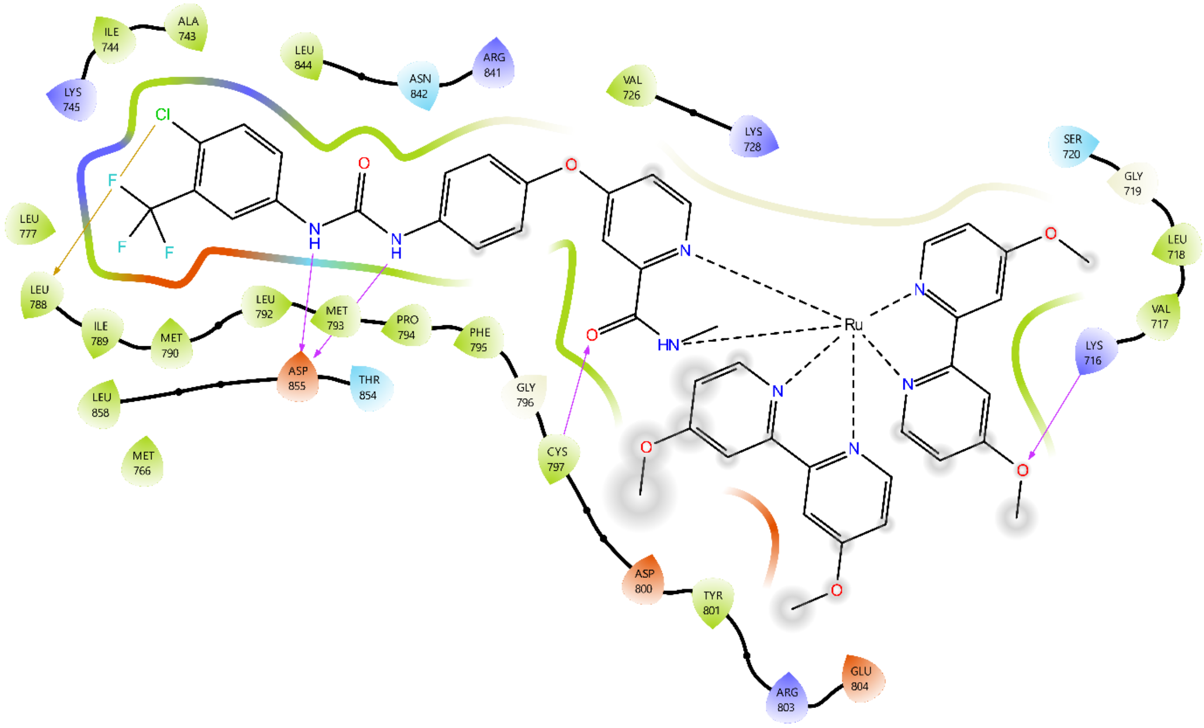

Figure S68. Molecular docking 2D ligand protein interactions between **Ru5S** and the active site of EGFR (PDB: 5X2A)

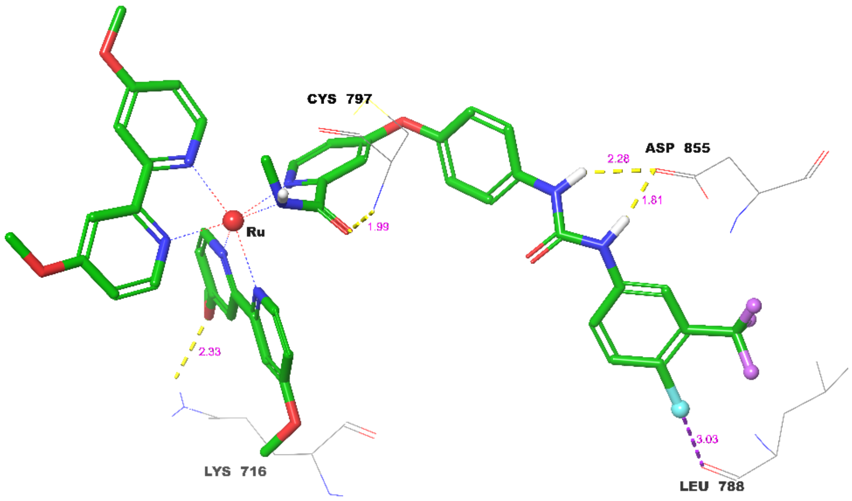

Figure S69. Molecular docking 3D ligand protein interactions between **Ru5S** and the active site of EGFR (PDB: 5X2A)

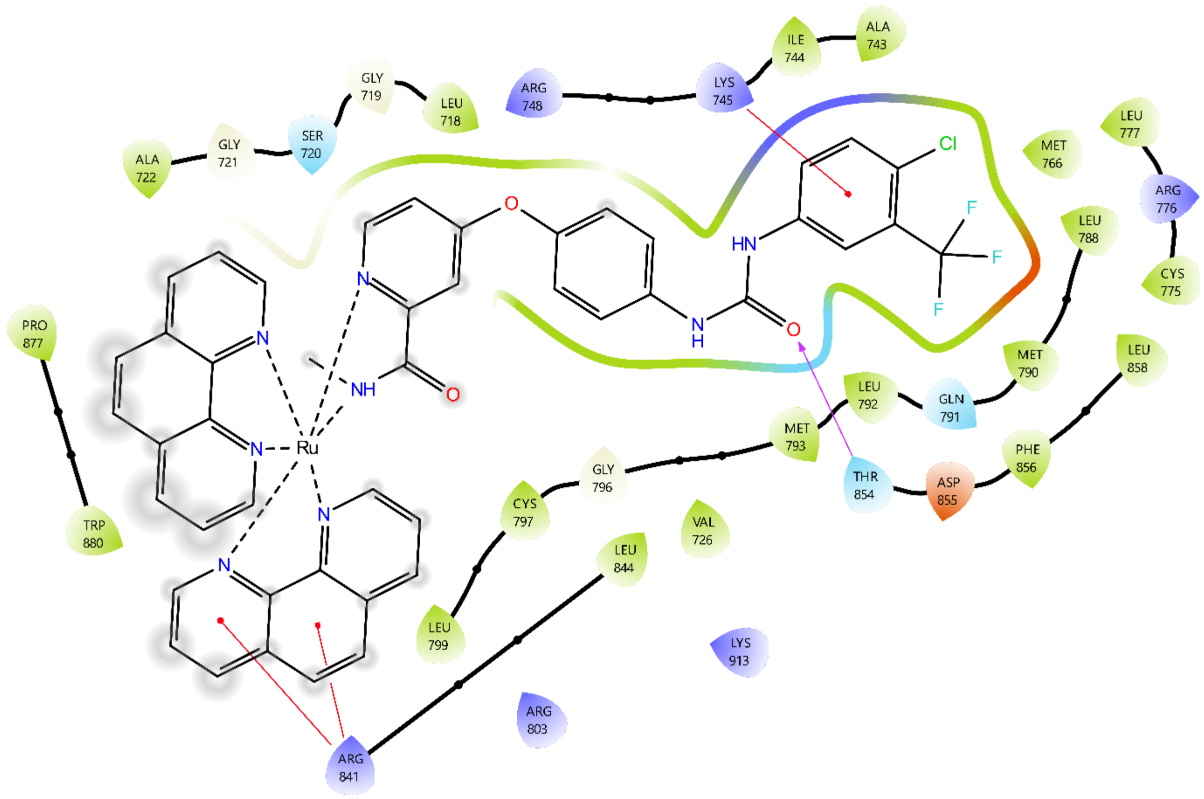

Figure S70. Molecular docking 2D ligand protein interactions between **Ru6S** and the active site of EGFR (PDB: 5X2A)

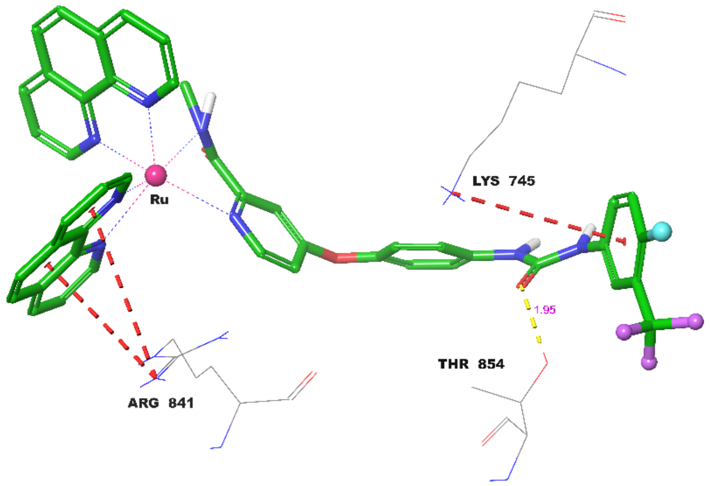

Figure S71. Molecular docking 3D ligand protein interactions between **Ru6S** and the active site of EGFR (PDB: 5X2A)

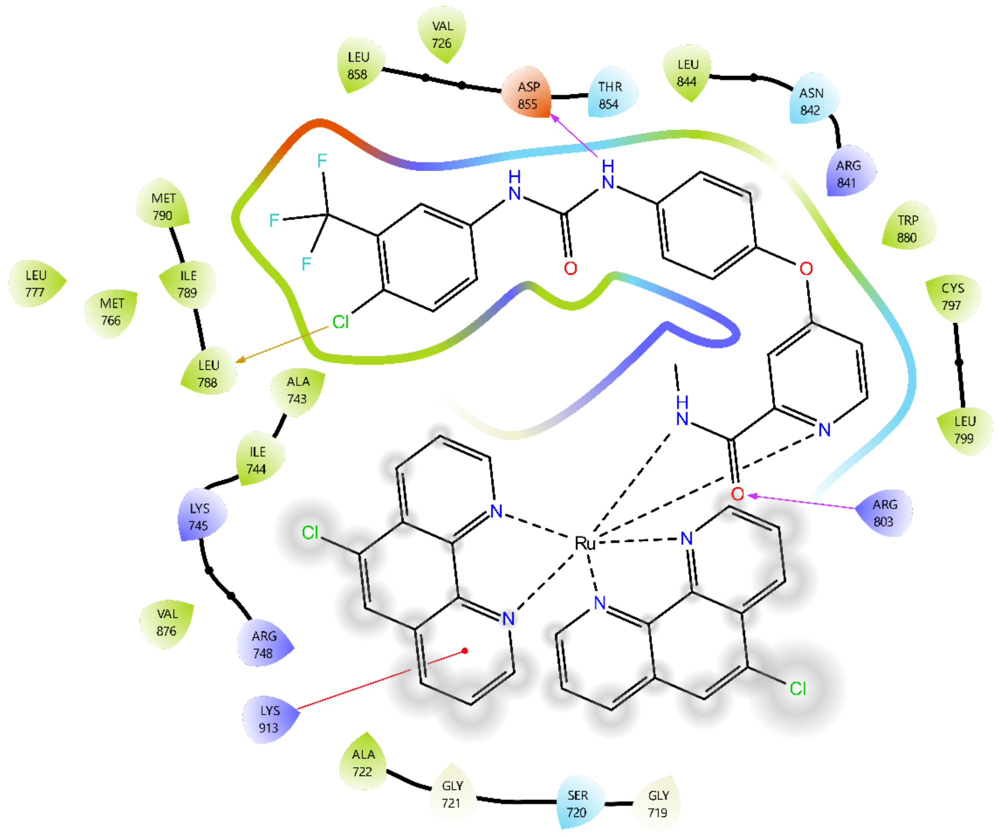

Figure S72. Molecular docking 2D ligand protein interactions between **Ru7S** and the active site of EGFR (PDB: 5X2A)

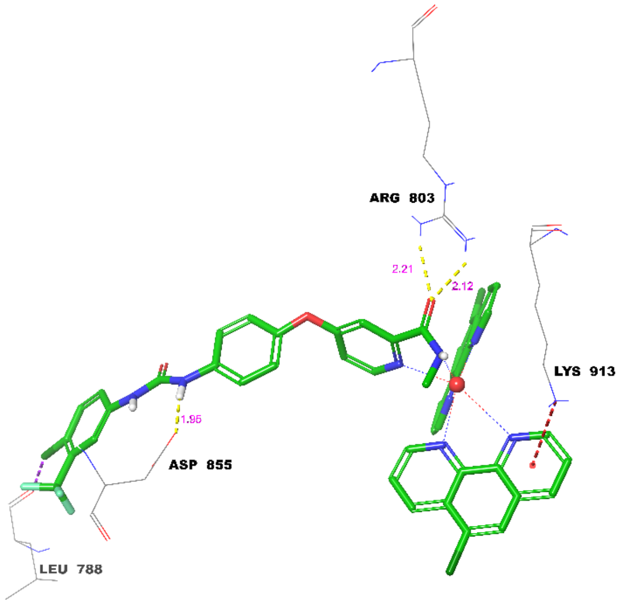

Figure S73. Molecular docking 3D ligand protein interactions between **Ru7S** and the active site of EGFR (PDB: 5X2A)

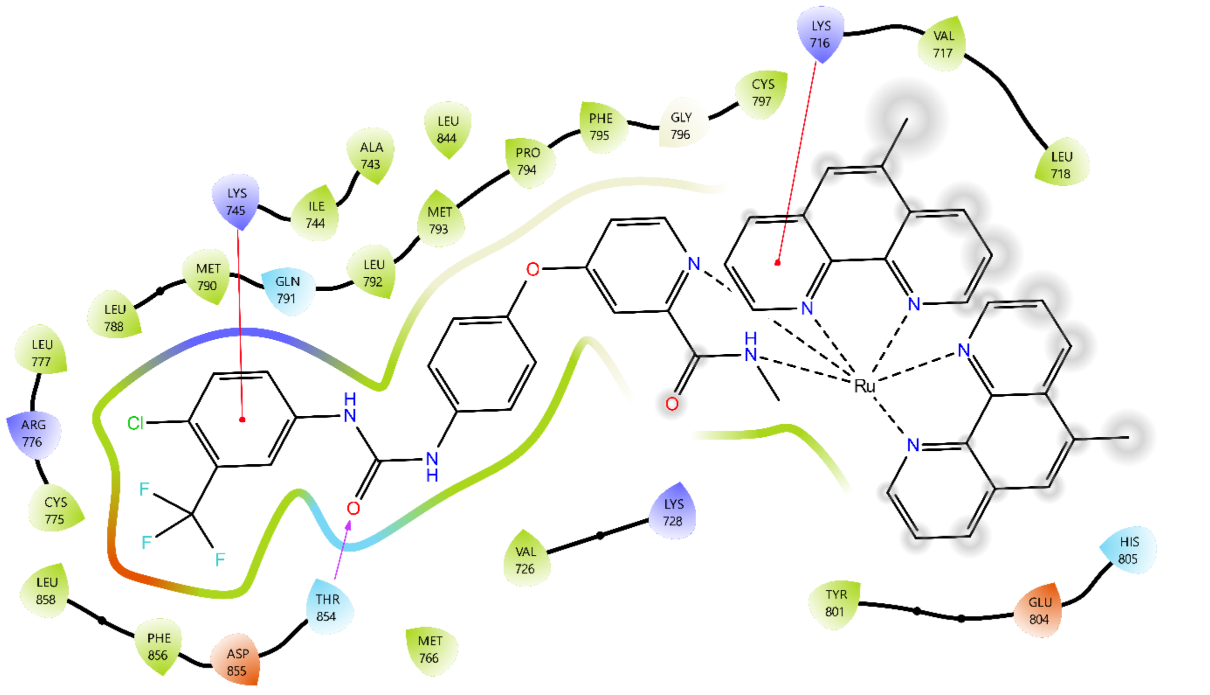

Figure S74. Molecular docking 2D ligand protein interactions between **Ru8S** and the active site of EGFR (PDB: 5X2A)

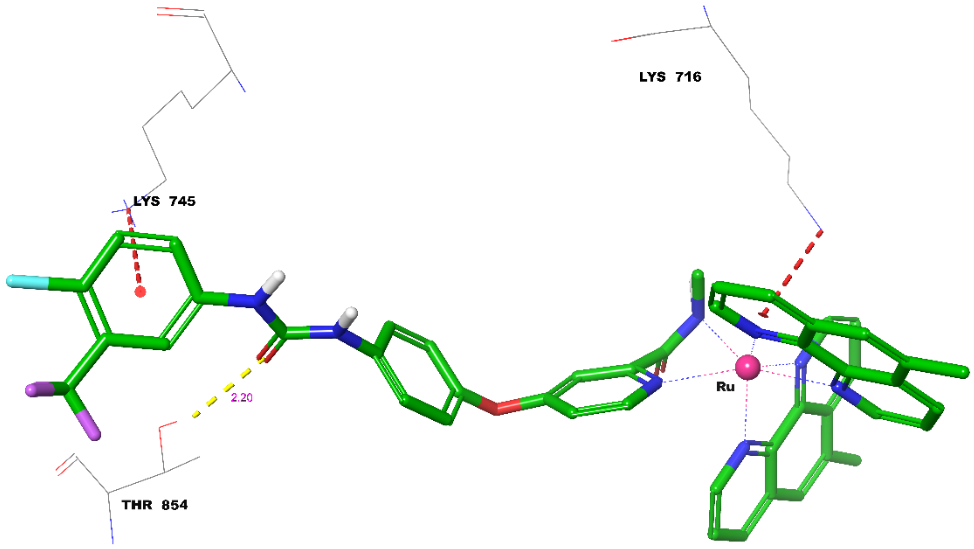

Figure S75. Molecular docking 3D ligand protein interactions between **Ru8S** and the active site of EGFR (PDB: 5X2A)

Molecular Docking Validations

The co-crystal ligand 7XO (N2-[4-(4-methylpiperazin-1-yl)phenyl]-N8-phenyl-9-propan-2-yl-purine-2,8-diamine), extracted from EGFR (PDB ID: 5X2A), underwent a validation process by re-docking at its original crystal positions without any alterations or conformational changes. This procedure aimed to validate the precision of molecular docking methods and protocols [1]. The superimposition of the original crystallographic conformation with the co-crystallized ligand's re-docked pose yielded a Root-Mean Square Deviation (RMSD) of 0.3842 Å, indicating a high level of accuracy in reproducing the binding pose **(Figure S76).**

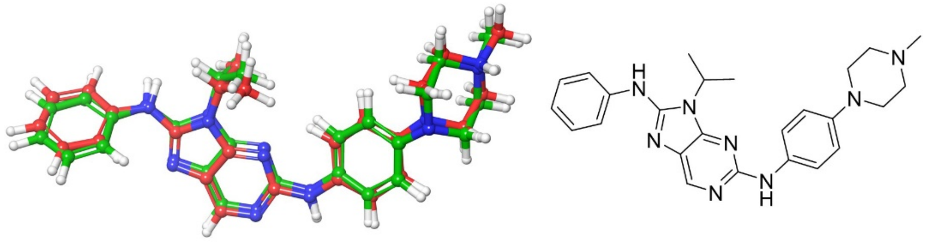

7XO (Co-crystal of **5X2A**), RMSD: 0.3842 Å

Figure S76. Molecular docking validation poses of the EGFR (PDB ID: 5X2A)

Figure S76 visually represents this validation, with the co-crystallized ligand shown in green and the re-docked ligand represented in red balls and sticks. Typically, RMSD values below 2 Å are considered indicative of correct predictions, while values in the 2-3 Å range are generally deemed acceptable for binding pose accuracy assessment [2].

Table S1. Elemental analysis results for the complexes

		**Calculated**			**Found**		
**Complex**	**Molecule Formula**	**% C**	**% H**	**% N**	**% C**	**% H**	**% N**
**Ru1S**	$C_{41}H_{32}ClF_3N_8O_3Ru$	56.07	3.67	12.76	55.87	3.42	12.98
**Ru2S**	$C_{45}H_{40}ClF_3N_8O_3Ru$	57.84	4.31	11.99	58.04	4.42	11.76
**Ru3S**	$C_{45}H_{40}ClF_3N_8O_3Ru$	57.84	4.31	11.99	57.45	4.28	11.75
**Ru4S**	$C_{57}H_{64}ClF_3N_8O_3Ru$	62.09	5.85	10.16	62.35	5.84	10.05
**Ru5S**	$C_{45}H_{40}ClF_3N_8O_7Ru$	54.14	4.04	11.22	54.75	4.25	11.87
**Ru6S**	$C_{45}H_{32}ClF_3N_8O_3Ru$	58.35	3.48	12.10	58.21	3.49	12.05
**Ru7S**	$C_{45}H_{30}Cl_3F_3N_8O_3Ru$	54.31	3.04	11.26	54.25	2.99	11.18
**Ru8S**	$C_{47}H_{36}ClF_3N_8O_3Ru$	59.15	3.80	11.74	59.31	3.76	11.56

(1) Şenol, H.; Ağgül, A. G.; Atasoy, S. Synthesis, characterization, molecular docking and in vitro biological studies of thiazolidin-4-one derivatives as anti-breast-cancer agents. *ChemistrySelect* **2023**, *8* (20), e202300481.
(2) Şenol, H.; Ağgül, A. G.; Atasoy, S.; Güzeldemirci, N. U. Synthesis, characterization, molecular docking and in vitro anti-cancer activity studies of new and highly selective 1,2,3-triazole substituted 4-hydroxybenzohyrdazide derivatives. *J. Mol. Struct.* **2023**, *1283*, 135247.
